# Acyl chain selection couples the consumption and synthesis of phosphoinositides

**DOI:** 10.15252/embj.2021110038

**Published:** 2022-06-30

**Authors:** David Barneda, Vishnu Janardan, Izabella Niewczas, Daniel M Collins, Sabina Cosulich, Jonathan Clark, Len R Stephens, Phillip T Hawkins

**Affiliations:** ^1^ Signalling Programme Babraham Institute Cambridge UK; ^2^ Projects, Oncology R&D AstraZeneca Cambridge UK; ^3^ Cellular Organization and Signalling National Centre for Biological Sciences Bangalore India

**Keywords:** CDP‐DG, CDS, DGK, H_2_^18^O, PI, Membranes & Trafficking

## Abstract

Phosphoinositides (PIPn) in mammalian tissues are enriched in the stearoyl/arachidonoyl acyl chain species (“C38:4”), but its functional significance is unclear. We have used metabolic tracers (isotopologues of inositol, glucose and water) to study PIPn synthesis in cell lines in which this enrichment is preserved to differing relative extents. We show that PIs synthesised from glucose are initially enriched in shorter/more saturated acyl chains, but then rapidly remodelled towards the C38:4 species. PIs are also synthesised by a distinct ‘re‐cycling pathway’, which utilises existing precursors and exhibits substantial selectivity for the synthesis of C38:4‐PA and ‐PI. This re‐cycling pathway is rapidly stimulated during receptor activation of phospholipase‐C, both allowing the retention of the C38:4 backbone and the close coupling of PIPn consumption to its resynthesis, thus maintaining pool sizes. These results suggest that one property of the specific acyl chain composition of PIPn is that of a molecular code, to facilitate ‘metabolic channelling’ from PIP2 to PI via pools of intermediates (DG, PA and CDP‐DG) common to other lipid metabolic pathways.

## Graphical abstract

Pathways of phosphatidylinositol synthesis are uncovered to reveal intricate functional adaptation.

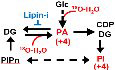

## Introduction

Phosphoinositides (PIPn) are a family of membrane phospholipids that are classified according to the structure of their phosphorylated inositol headgroups (PI, PI3P, PI4P, PI5P, PI(3,4)P_2_, PI(3,5)P_2_, PI(4,5)P_2_ and PI(3,4,5)P_3_). They are known to play a wide range of important regulatory functions in cells, including signal transduction, membrane identity and the sorting of proteins and lipids within intracellular compartments (Balla 2013; Dickson & Hille, 2019; Hammond & Burke, 2020). They are interconverted by specific lipid kinases and phosphatases which add or remove phosphates on their inositol headgroups and the main principle by which they act is via the specific recognition of these headgroups through conserved domains in effector proteins (e.g. PH, PX and FYVE domains) (Hammond & Balla, 2015).

Each headgroup‐defined class of phosphoinositide is also comprised of multiple molecular species that differ in the aliphatic chains that are linked via acyl or alkyl linkages to their glycerol backbone (Harayama & Riezman, 2018). This feature has received little recognition to date, partly because these lipids are thought to function primarily via electrostatic interactions with their head groups but also because the methods used to measure these lipids have not generally distinguished between different alkyl/acyl species. However, recent developments in the use of mass spectrometry approaches to measure phosphoinositides, particularly the more highly phosphorylated classes (Wenk *et al*, 2003; Pettitt *et al*, 2006; Wang *et al*, 2016; Clark *et al*, 2011; Traynor‐Kaplan *et al*, 2017; Bui *et al*, 2018), have highlighted an unusual feature of these lipids, first recognised in PI in the 1970s (Akino & Shimojo, 1970; Holub & Kukis, 1971), that phosphoinositides extracted from mammalian tissues and primary cells are surprisingly molecularly homogenous compared with most other phospholipid classes, with a strong enrichment (usually > 70% and often > 90%) in the stearoyl/arachidonoyl species (Lee *et al*, 2012; Anderson *et al*, 2013). However, this enrichment is often lost in cells grown for substantial periods of time in culture or in tumours, probably due to both genetic (e.g. cell transformation) and environmental (e.g. availability of acyl‐CoAs) factors (Rouzer *et al*, 2006; Kawashima *et al*, 2013; Goto *et al*, 2014; Naguib *et al*, 2015; Anderson *et al*, 2016; Traynor‐Kaplan *et al*, 2017). The potential biological significance of maintaining a distinct acyl chain profile in phosphoinositides is also emphasised by the finding that in Dictyostelium a totally distinct ether‐linked species is concentrated into their phosphoinositide pools (Clark *et al*, 2014).

A substantial body of work now indicates the acyl chain composition of most phospholipid classes is determined in large part by the remodelling of lipids made *de novo* by the “Lands cycle”; a combination of PLA1 and 2 phospholipases, that remove the acyl chains in the sn‐1 and sn‐2 positions, respectively, and acyl‐CoA transferases with varying specificity for the acyl‐CoA species and lysophospholipid acceptor (Shindou *et al*, 2009; Blunsom & Cockcroft, 2019). Indeed, acyl‐CoA transferases with selectivity for stearate at sn‐1 and arachidonate at sn‐2 of PI have been described, but knock‐out studies have suggested these two activities can only be part of the mechanism for achieving molecular homogeneity (Barneda *et al*, 2019). An alternative hypothesis has also been proposed, that there is selective conversion of stearoyl/arachidonoyl diacylglycerol (DG), via phosphatidic acid (PA), to cytidine diphosphate‐diacylglycerol (CDP‐DG) destined for PI synthesis, a pathway catalysed by the two enzymes DGKε and CDS2 (D'Souza & Epand, 2014), but any evidence for this mechanism *in vivo* is still lacking.

In addition to the lack of clarity for how the acyl chain homogeneity of phosphoinositides is created *in vivo*, the question arises as to how/if this is preserved during activation of the phospholipase C (PLC) signalling pathway. Receptor‐stimulated PLCs hydrolyse PI(4,5)P_2_ to form the second‐messengers inositol (1,4,5)‐trisphosphate (IP_3_) and diacylglycerol (DG) (Gallegos & Newton, 2008; Thillaiappan *et al*, 2018), thus potentially separating the information as to which headgroup was attached to the now liberated DG. This increased concentration of DG is then thought to stimulate re‐synthesis of PI, via PA and CDP‐DG, to replenish the pools of PI, PI4P and PI(4,5)P_2_, thus completing a “PI cycle” (Michell 1975). Indeed, the PLC pathway was first discovered through the hormone‐stimulated incorporation of radioactive tracers into PI, although the extent to which the PI cycle is “closed,” with little mixing of the common biosynthetic intermediates (DG, PA and CDP‐DG) with other lipid pools, is unclear and has been disputed (Cockcroft & Allan, 1984). Furthermore, the potential molecular basis for this organisation, if indeed it occurs, is also unclear.

An assumption‐free approach to tracking the metabolism of different molecular species of phosphoinositides would be to use different types of molecular tracers. In the past, radioactive glycerol, inositol and phosphate have been used to follow phosphoinositide synthesis in basal and hormone‐stimulated conditions, but the vast majority of these studies did not distinguish acyl chain variants and, the few that did, employed heterogenous tissues, broken cell systems and/or relatively crude analytical techniques, making interpretation difficult (Holub & Kukis, 1971; Luthra & Sheltawy, 1976; Nakagawa *et al*, 1989). We set out to explore the use of modern mass spectrometry techniques coupled with the use of both existing and newly synthesised isotopologue precursors to track the synthesis and fate of phosphoinositides in cell lines with varying degrees of stearoyl/arachidonoyl enrichment. The results suggest an important property of the stearoyl/arachidonoyl backbone is to allow efficient channelling of PLC‐derived DG towards re‐synthesis of PI and PIP2.

## Results

### Phosphoinositides are enriched in C38:4 species compared with their biosynthetic precursors

**Figure 1:**
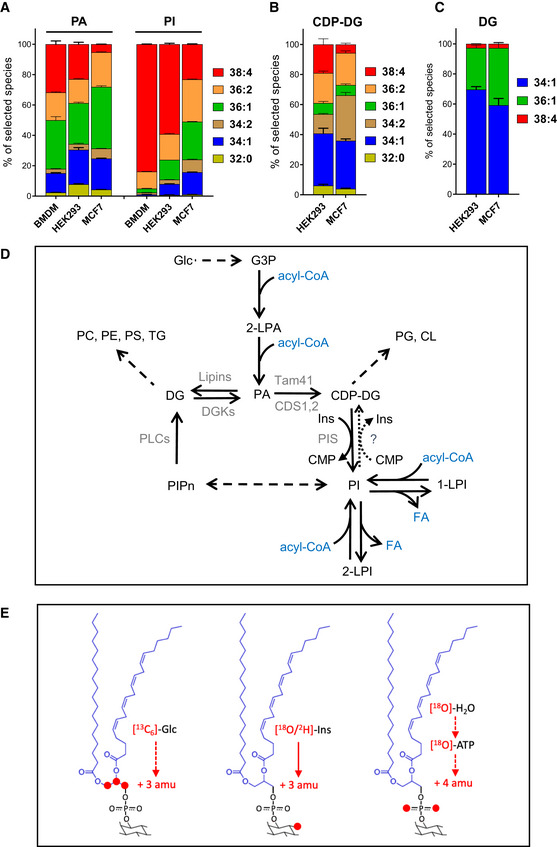
Phosphoinositides are enriched in C38:4 species compared with their biosynthetic precursors AThe proportions of the major acyl chain species of PA and PI in BMDMs, HEK293 and MCF7; values were calibrated using the data presented in Fig EV1A and B. Data are represented as mean ± SD (*n* = 3) from a single experiment, typical of at least three performed.BThe proportions of different acyl chain species of CDP‐DG in HEK293 and MCF7 (uncalibrated). Data are represented as mean ± SEM of biological replicates (*n* = 4).CThe proportions of different acyl chain species of DG in HEK293 and MCF7; values were calibrated using the data presented in Fig EV1C. Data are represented as mean ± SEM of biological replicates (*n* = 3).DBiosynthetic pathway for synthesis of PIPn.ESchematic diagram showing the three isotopologue labelling strategies used in this study. Data information: See also Fig EV1.

**Figure EV1:**
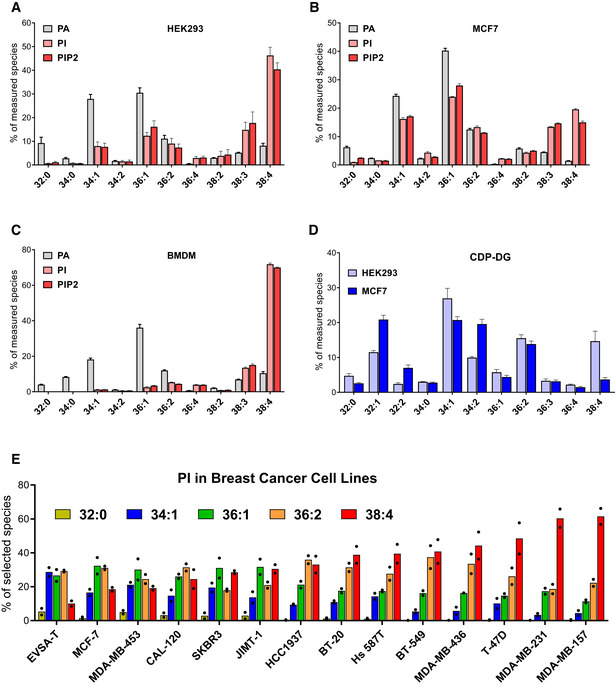
Acyl chain composition of phosphoinositides in different cell lines A–CThe proportions of all the targeted molecular species of PA, PI and PIP2 measured in BMDMs, HEK293 and MCF7 before applying the correction factors derived from the calibration curves shown in Appendix Fig S1. Data are represented as mean ± SD (*n* = 3) from a single experiment, typical of at least three performed.DComparison of all CDP‐DG species measured in HEK293 and MCF7. Data are represented as mean ± SEM of 4 biological replicates.EDistribution of PI species in a panel of breast cancer cell lines cultured in equivalent conditions before lipid extraction (200,000 cells/well seeded in 6‐well plates and incubated 24 h in RPMI with 10% FBS and Pen/Strep). Data are represented as individual points with bisecting bars from 2 independent experiments.

We used LC–MS to quantify the major acyl chain variants of phosphoinositides and their metabolic precursors in HEK293 cells, MCF7 cells and primary bone‐marrow‐derived macrophages (BMDM), see Fig 1A and C. The final steps in the pathway for *de novo* PI synthesis in mammals involve the conversion of PA to CDP‐DG, then the conversion of CDP‐DG to PI (Fig 1D; Blunsom & Cockcroft, 2019). In each of the cell types analysed, there is a clear enrichment of some acyl chain species in PI compared with CDP‐DG and PA (Figs 1A and B, and EV1A–D). This is most clearly the case with the stearoyl/arachidonoyl species (“C38:4”; our mass spectrometry measurements report the combined total number of carbons:double bonds in both acyl chains), although the final proportion of C38:4‐PI was substantially greater in some cell types than others; BMDM (80%) > HEK293 (60%) > MCF7 (20%) (note MCF7 cells possess lower C38:4 enrichment compared with several other breast‐derived cell lines grown under identical culture conditions, see Fig EV1E). Furthermore, the proportion of the C38:4 species was very similar within each cell type between PI and PIP2 (Fig EV1A–C), consistent with their rapid interconversion via kinases and phosphomonoesterases (Balla 2013). In Fig 1A and C, we only present the data for the major acyl chain species of PI, PA and DG for which we have conducted calibration curves with synthetic standards to correct for differences in the efficiency of detection by LC–MS (see Appendix Fig S1, but uncalibrated data for the proportions of all acyl chain species of PI and PA measured are shown in Fig EV1A–D).

**Figure 2:**
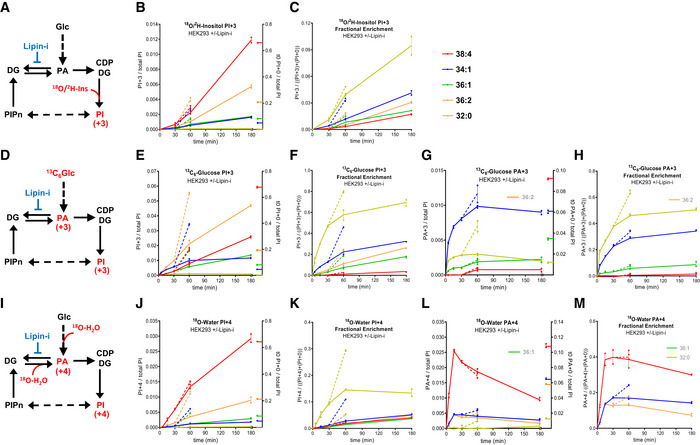
HEK293 cells enrich for the C38:4 species of PI via multiple pathways ASchematic diagram illustrating the incorporation of ^18^O/^2^H‐inositol into PI (‘PI+3’).BThe formation of “PI+3” in cells incubated with ^18^O/^2^H‐inositol.CThe fractional enrichment of “PI+3” in cells incubated with ^18^O/^2^H‐inositol.DSchematic diagram illustrating the incorporation of ^13^C_6_‐glucose into PA and PI (“PA+3”; “PI+3”).EThe formation of “PI+3” in cells incubated with ^13^C_6_‐glucose.FThe fractional enrichment of “PI+3” in cells incubated with ^13^C_6_‐glucose.GThe formation of “PA+3” in cells incubated with ^13^C_6_‐glucose.HThe fractional enrichment of “PA+3” in cells incubated with ^13^C_6_‐glucose.ISchematic diagram illustrating the incorporation of ^18^O‐water into PA and PI (“PI+4”; “PA+4”).JThe formation of “PI+4” in cells incubated with ^18^O‐water.KThe fractional enrichment of “PI+4” in cells incubated with ^18^O‐water.LThe formation of “PA+4” in cells incubated with ^18^O‐water.MThe fractional enrichment of “PA+4” in cells incubated with ^18^O‐water. Data information: B, E, G, J and L: data are the values for the indicated isotopologues normalised to the total level of all PI species (i.e. all acyl chain species combined), to correct for differences in cell mass (for comparison, the steady‐date levels of the unlabelled species at *t* = 0 are shown on the right axes). Where indicated, 200 μM propranolol (Lipin‐i) was added at 30 min and incubations continued for a further 30 min (broken lines). C, F, H, K and M: data are values for the “fractional enrichment” of the indicated isotopologue (the ratio between the labelled isotopologue and the sum of labelled and unlabelled isotopologues). Where indicated, 200 μM propranolol (Lipin‐i) was added at 30 min and incubations continued for a further 30 min (broken lines). Data are represented as individual points with bisecting lines (*n* = 2 wells/condition) from a single experiment in which all three labelling strategies were performed in parallel, in equivalent media. The results are representative of three similar experiments where each of the labelling strategies were performed in slightly different media, two of which included the addition of propranolol. Where the measurement of an individual species has been omitted for technical reasons, it is ‘greyed out’ in the legend. See also Fig EV2.

Phosphatidic acid sits at a major intersection of metabolic pathways in lipid synthesis; it can be synthesised *de novo* from glycerol phosphate, via phospholipase D (PLD)‐catalysed cleavage of PC, or via DG kinase (DGK)‐catalysed phosphorylation of DG; it also represents a branch point in the *de novo* synthesis of phospholipids, it can be dephosphorylated to DG (providing the substrate for PC, PE and PS) or converted to CDP‐DG (providing the substrate for PI, PG and CL; Fig 1D). Furthermore, recent evidence suggests CDP‐DG synthesised in the ER by CDS1/2 is selectively converted to PI, but CDP‐DG synthesised in the mitochondria by TAMM41 is directed to PGP, PG and CL (Blunsom & Cockcroft, 2020). Given this complexity, and the difficulty in physically separating potential precursor‐product pools within membrane or sub‐membrane compartments, we turned to an isotopologue tracing strategy to investigate the kinetics of acyl chain enrichment in PI pools. We used ^18^O/^2^H‐inositol (see Appendix Fig S3) to measure PIs with a new inositol head‐group; ^13^C_6_‐glucose to measure PIs with a new glycerol backbone and H_2_^18^O (^18^O‐water) to measure PIs with a new diester‐phosphate (see Fig 1E for the isotopologues measured and Figs 2A, D and I for their pathways of incorporation).

### HEK293 enrich for the C38:4 species of PI via multiple pathways

**Figure EV2:**
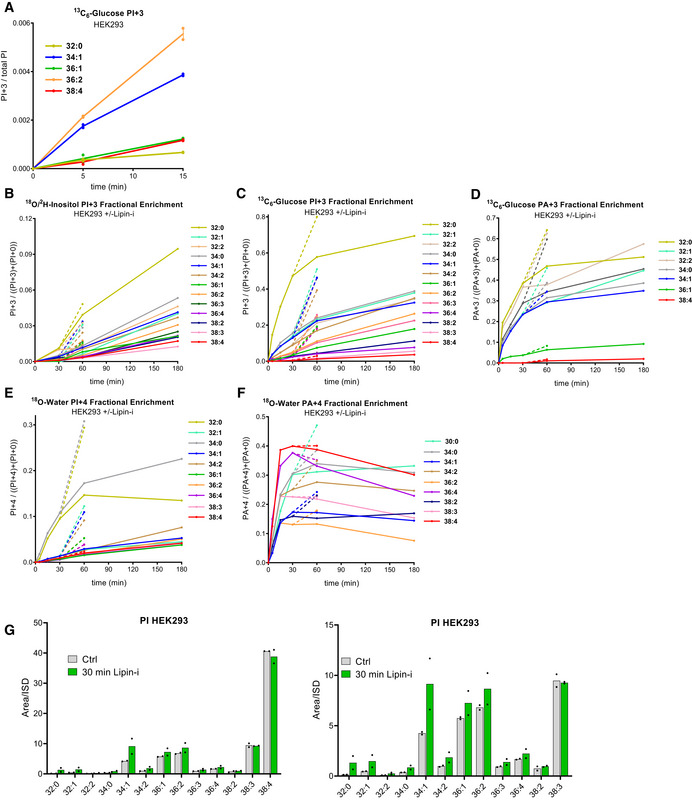
PI and PA synthesis in HEK293 cells ARe‐scaled graph of the early time points only from main Fig 2E, showing the early incorporation of ^13^C_6_‐glucose into the glycerol backbone of PI, highlighting the lagged accumulation of labelled C38:4‐PI.B–FThe fractional enrichment of the indicated isotopologues of PA and PI during the incubation of HEK293 cells with ^18^O/^2^H‐inositol (“PI+3”), ^13^C_6_‐glucose (“PI+3”; “PA+3”) and ^18^O‐water (“PI+4”; “PA+4”), calculated for each species as the ratio between the labelled isotopologue and the sum of labelled and unlabelled isotopologues. All the targeted molecular species with an adequate signal/background are shown, as this parameter is not affected by differences in the detection efficacy between molecular species. Where indicated, 200 μM propranolol (Lipin‐i) was added at 30 min and incubations continued for a further 30 min (broken lines).GChanges in the levels of unlabelled PI species after 30‐min treatment with 200 μM propranolol (Lipin‐i). Data represent the response ratio vs. the PI ISD of all targeted species before calibration. Data information: Data in panels A‐F are represented as the means (*n* = 2 wells/condition) from a single experiment in which all three labelling strategies were performed in parallel, in equivalent media. The results are representative of three similar experiments where each of the labelling strategies were performed in slightly different media, two of which included the addition of propranolol. Data in G are represented as individual points with bisecting bars from 2 independent experiments.

The initial rates of incorporation of +3 amu from ^18^O/^2^H‐inositol (“PI+3”), +3 amu from ^13^C_6_‐glucose (“PI+3”; “PA+3”) and +4 amu from ^18^O‐water (“PI+4”; “PA+4”) into the different acyl chain species of PI and PA in HEK293 cells are shown in Fig 2. We present both calibrated values for the amount of isotopologue measured and also its “fractional enrichment” (a measure of the proportion of the pool labelled). Fractional enrichments are independent of cross‐species calibration and additional fractional enrichment data for all species measured is shown in Fig EV2.

After a significant lag of approximately 30 min (due to the relatively slow equilibration of inositol across the plasma membrane), the relative rates of ^18^O/^2^H‐inositol incorporation were in the order C38:4‐PI > C36:2‐PI > C36:1 = C34:1‐PI > C32:0‐PI (Fig 2B). The fractional enrichments achieved under these conditions were relatively low (due to the low starting enrichment, the slow equilibration of inositol across the plasma membrane and the significant pool of intracellular inositol), but within the error of these measurements the smaller pool of C32:0‐PI appeared to turn over at a greater rate than the other species (Figs 2C and EV2B).

The incorporation of ^13^C_3_‐glycerol units (Fig 2D) showed a very different pattern, characterised by a markedly lower and distinctly lagged accumulation of labelled C38:4‐PI (Figs 2E and EV2A). This lag in the labelling of C38:4‐PI was in contrast to the labelling of the other species analysed, which exhibited no obvious lag and presented apparently simpler saturation kinetics (Figs 2E and EV2C). The C38:4‐PI pool also labelled at a much lower fractional rate (Fig 2F). Furthermore, a close examination of the fractional enrichment curves for all species measured (Fig EV2C) suggests a trend for shorter chain, more saturated PIs (e.g. C32:0, C34:0, C32:1, C34:1) to turn over faster with respect to their glycerol backbone than the longer chain, more unsaturated species (eg C38:2, C38:3, C38:4, C36:4). The labelling of these PI pools will be dictated, in part, by the labelling kinetics of their precursors; unfortunately, our methods were not sensitive enough to obtain a comprehensive analysis of the labelling of different CDP‐DG species, but we were able to obtain useful data for the labelling of different PAs (Fig 2G and H; ^13^C‐labelled C36:2‐PA could not be measured accurately in HEK293 due to mass contamination with other molecules). This data confirmed that the shorter chain, more saturated PAs incorporate new glycerol units more quickly than the other acyl chain variants, with very low levels of [+3]‐glycerol incorporation into C38:4‐PA. Overall, these differences in kinetics suggest a precursor‐product relationship may exist between C32:0‐PI and C38:4‐PI. This data also indicate that the majority of the ^18^O/^2^H‐inositol incorporated into C38:4‐PI shown in Fig 2A does not represent synthesis from PAs derived *de novo* (otherwise the labelling with glucose and inositol would be more similar). Additional potential routes for ^18^O/^2^H‐inositol incorporation would include PI synthesis from pre‐existing PA or, via the so‐called “exchange reaction,” where PIS is able exchange inositol head groups in PI with free inositol without release of CMP/CDP‐DG ie with no nett PI synthesis. This exchange reaction has been demonstrated *in vitro* but not in cells (Fischl *et al*, 1986).

To distinguish between these possibilities and to obtain further insights into the pathways of PI synthesis, we developed an isotopologue tracing strategy based on labelling with ^18^O‐water (Figs 1E and 2I). We found that ^18^O‐nuclei from water equilibrate rapidly with the cell's nucleotide pools (Versaw & Metzenberg, 1996) and can be used to trace phosphate incorporation into both PA and PI. The incorporation of ^18^O‐phosphate into different PI species followed a similar rank order to that seen with ^18^O/^2^H‐inositol (Fig 2J vs. 2B), suggesting both tracers reported synthesis of new PIs. Furthermore, the faster entry of ^18^O‐water revealed that C38:4‐PI was the most highly labelled species, even at the very earliest times, extending the contrast with the lagged labelling of this species with ^13^C_3_‐glycerol units (Figs 2J vs. EV2A). Fractional enrichment measurements again supported the conclusion that the smaller pool of C32:0‐PI was turned over at a fast rate (Figs 2K and EV2E). The incorporation of label into PA also revealed that C38:4‐PA was synthesised from DG at a much faster rate than the other species (Fig 2L), with a t_1/2_ < 2–3 min (Fig 2M). Furthermore, this rapidly labelled sub‐pool of C38:4‐PA appeared to be a much higher fraction of the total pool for this species than for the rapidly labelled sub‐pools of some of the other PAs (Fig 2M).

The above analysis suggests that PIs made from PAs derived directly via glycolysis make up a relatively small proportion of all PIs made. To test this idea, we attempted to selectively perturb the pool sizes of those PAs acting primarily in *de novo* phospholipid synthesis by using the lipin inhibitor propranolol (Reue & Wang, 2019). Propranolol was added at 30 min and labelling followed for a further 30 min (Fig 2; broken lines). Addition of propranolol dramatically enhanced ^13^C_3_‐glycerol incorporation into PIs and PAs (Fig 2E and G), with the biggest effects on those species with the highest fractional turnover (C32:0, C34:0, C32:1, C34:1), consistent with the relative effects of propranolol on the total levels of these species (Fig EV2G) and thus identifying these species as the ones made preferentially *de novo*. In contrast, propranolol had much smaller effects on both ^18^O/^2^H‐inositol (Fig 2B) and ^18^O‐phosphate incorporation (Fig 2J), with negligible effects on ^18^O/^2^H‐inositol and ^18^O‐phosphate incorporation into the C38:4‐PI species (Fig 2B and J). This selective stimulation of the *de novo* route allows a cross‐calibration of the different labelling strategies and confirms that the bulk of C38:4‐PI synthesis in HEK293 occurs via C38:4‐PA derived from C38:4‐DG by DGK, and not via PA made by *de novo* synthesis. We estimate that the maximum contribution that the pathway reported by ^13^C_3_‐glycerol incorporation could make to total C38:4‐PI synthesis is 15–20% [this estimate is based on using the average change in labelling induced by propranolol amongst the five species measured to convert units of inositol (×13.1 ± 1.4) and water (×4.8 ± 0.9) incorporation into equivalent units of glycerol incorporation]. In contrast, this estimate is in the range 60–100% for the other acyl chain species.

### Investigating a role for DGK and CDS isoforms in acyl chain selective synthesis of PI

**Figure 3:**
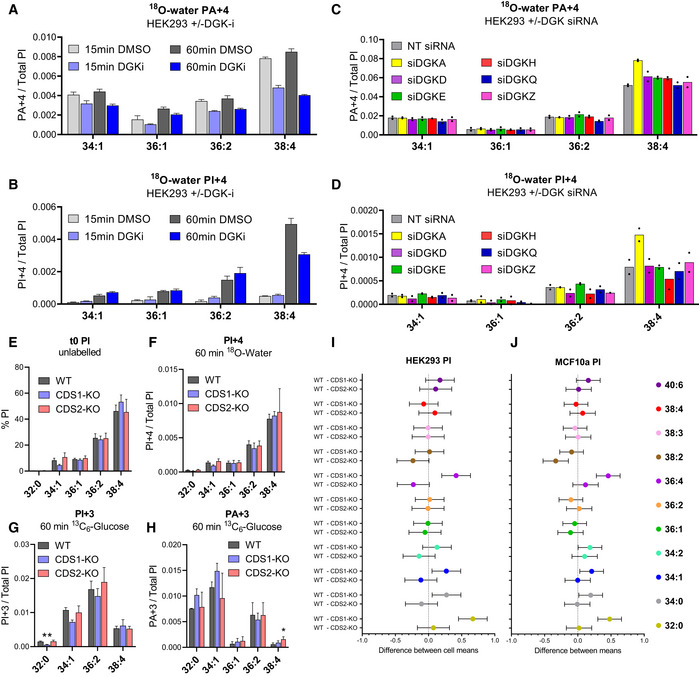
Investigating a role for DGK and CDS isoforms in acyl chain selective synthesis of PI A, BValues for the indicated PI and PA isotopologues in HEK293 cells treated with DGK inhibitor R59949 (30 μM) or vehicle (DMSO) for 5 min and then incubated with ^18^O‐water for a further 15 or 60 min, in the continued presence of the inhibitor or vehicle. Data labels are as described in the legend to Fig 2. Data are represented as mean ± SEM (*n* = 3 biological replicates).C, DValues for the indicated PI and PA isotopologues in HEK293 cells labelled for 15 min with ^18^O‐Water, performed 48 h after transfection with SMARTpool siRNAs directed against the indicated targets. Data labels are as described in the legend to Fig 2. Data are represented as individual points with bisecting bars (*n* = 2 independent experiments).E–HValues for the indicated isotopologues of PI and PA in WT HEK293 clones, or clones in which the genes encoding CDS1 or CDS2 had been deleted (CDS1‐KO; CDS2‐KO). The proportions of the individual PI (E) acyl chain species at steady‐state are shown (t0 PI), together with the formation of the indicated isotopologues after incubation with ^13^C_6_‐glucose (G and H) or ^18^O‐water (F) for 60 min. Data are represented as mean ± SEM (*n* = 3 separately derived clones). Log‐transformed data were analysed with a 2‐way ANOVA followed by Dunnett's multiple comparisons tests (*P ≤ 0.05 vs. WT, **P ≤ 0.01 vs. WT).I, JChanges in the distribution of molecular species induced by deletion of CDS1 or CDS2 in HEK293 and MCF10a cells. The % of PI species in each genotype (*n* = 3 independently derived clones), calculated from the uncalibrated response ratios, were log‐transformed prior to the analysis to meet the assumptions for a parametric approach. Differences between genotypes for each molecular species were tested with a two‐way repeated measures ANOVA followed by Dunnett's multiple comparisons test. Data are shown as a multiple comparisons plot representing the differences between WT and KO with a 95% confidence interval. Data information: See also Fig EV3.

We sought confirmation that the majority of C38:4‐PI synthesis in HEK293 cells is via DGK‐catalysed formation of PA by measuring the effects of DGK inhibition. Short‐term incubation with a potent DGK inhibitor caused a rapid reduction in the rate of ^18^O‐water labelling of PA and PI, with much larger proportional effects on the C38:4 species (Fig 3A and B). This confirms that the majority of C38:4‐PA+4 and PI+4 measured in these water‐labelling studies is derived via a DGK‐catalysed step.

A comparison of the relative rates of initial PA+4 formation (Fig 2L) with the steady‐state levels of DG in these cells (Fig 1C) suggests DGK activity in these cells must be highly selective for the C38:4 species. There are 10 different DGKs expressed in mammalian cells (Cai *et al*, 2009), and DGKε has previously been shown to be selective for C38:4‐DG *in vitro* (D'Souza & Epand, 2012). We used siRNAs to knock‐down the expression of the six most highly expressed isoforms of DGK in HEK293 cells (Appendix Fig S3A) and measured their impact on ^18^O‐water labelling of PA and PI species (Fig 3C and D). Surprisingly, no individual knock‐down caused a significant reduction in the synthesis of PA+4 or PI+4 species, although knock‐down of DGK⍺ appeared to increase levels of C38:4‐PA+4 and ‐PI+4 (Fig 3C and D). This suggests that either the reduction in expression of the other isoforms in these experiments was insufficient to create an obvious phenotype or that C38:4‐selectivity is a property of multiple DGKs.

**Figure EV3:**
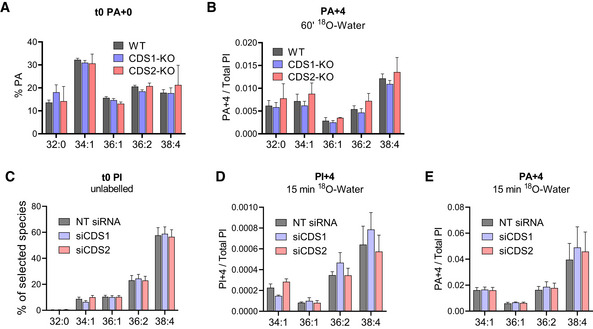
The role of CDS isoforms in acyl chain selective synthesis of PI A, BValues for the indicated isotopologues of PA in HEK293 clones in which the genes encoding CDS1 or CDS2 had been deleted (WT; CDS1‐KO; CDS2‐KO). The proportions of the individual PA acyl chain species at steady‐state are shown (t0 PA+0), together with the formation of the PA+4 isotopologue (PA+4) after incubation with ^18^O‐water for 60 min (normalised to the total level of all PI species to correct for differences in cell mass between clones). Data are represented as mean ± SEM (*n* = 3 separately derived clones).C–EValues for the indicated isotopologues of PI and PA in HEK293 cells labelled during 15 min with ^18^O‐water performed 48 h after transfection with siRNA SMARTpools against CDS1, CDS2 or NT controls. Silencing of the intended target was assessed by mRNA analysis (see Appendix Fig S4A). Data labels are as described in the legend to Fig 3. Data are represented as mean ± SEM of independent experiments (*n* = 3).

It has also been shown that CDS1 and CDS2 have different selectivity for CDP‐DG substrates *in vitro* and that CDS2 prefers the C38:4 species (D'Souza *et al*, 2014). We disrupted the genes encoding either CDS1 or CDS2 in HEK293 (see Appendix Fig S4B–D) and measured their effects on PA and PI metabolism. Loss of either CDS1 or CDS2 had remarkably little impact on either the steady‐state levels of the major species of PI (Fig 3E) or PA (Fig EV3A), or their labelling with ^13^C_6_‐glucose (Fig 3G and H) or ^18^O‐water (Figs 3F and EV3B), suggesting neither plays a major, non‐redundant role in C38:4‐PI enrichment in these cells under basal conditions. We did, however, note that loss of CDS1 did reduce the levels and labelling of some minor species of PI that feature prominently in the *de novo* synthesis pathway (e.g. C32:0, C34:1 and C34:0), or that would be predicted to be derived from them by simple acyl chain remodelling (e.g. C36:4, via sn‐2 remodelling of C32:0 with C20:4; Fig 3E, G and I; note there was also a corresponding increase in the labelling of equivalent species of PA, Fig 3H). This same pattern was also seen in analogous experiments where we deleted these two genes in MCF10 cells (Fig 3I vs. J).

It remained possible that a major role for CDS isoforms in acyl chain selection was obscured by long‐term adaptation to their loss, so we also analysed the effects of short‐term knock‐down in their expression by siRNA. However, we saw very similar, minor effects to those seen in the knock‐outs (Fig EV3C–E).

### PIs synthesised *de novo* undergo rapid LPIAT1‐dependent acyl chain remodelling

**Figure 4:**
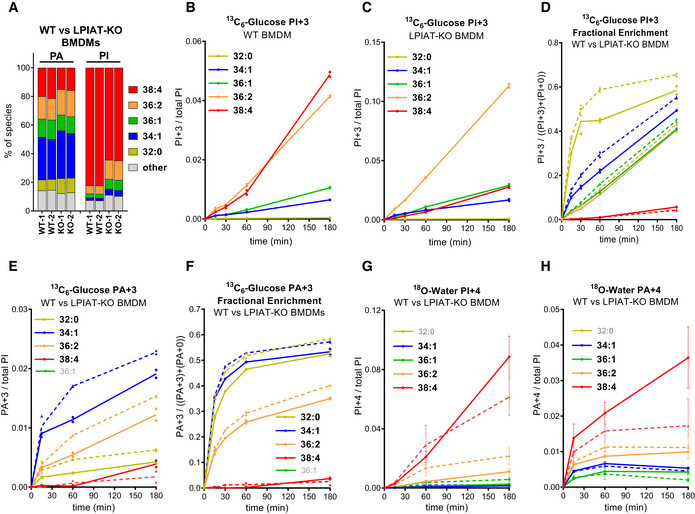
PIs synthesised *de novo* undergo rapid LPIAT1‐dependent acyl chain remodelling AThe proportions of individual acyl chain species of PA and PI at steady‐state in WT and LPIAT1‐KO BMDMs. These values have not been corrected using the calibration curves shown in Fig EV1E and F to include a wider range of species. Data are from two individual mice per genotype.B–FLabelling of PI and PA in WT or LPIAT‐KO BMDMs incubated with ^13^C_6_‐glucose. Data labels are as described in the legend to Fig 2. In panels D, E and F, data for WT BMDM are shown as solid lines and data for KO BMDM as broken lines. Where the measurement of an individual species has been omitted for technical reasons, it is “greyed out” in the legend. Data are represented as points from individual mice (*n* = 2) with bisecting lines. We obtained very similar data in a slightly different ^13^C‐labelling experiment using BMDMs derived from one further WT and KO mouse.G, HLabelling of PI and PA in WT or LPIAT‐KO BMDMs incubated with ^18^O‐water. Data labels are as described in the legend to Fig 2. Data for WT BMDM are shown as solid lines and data for KO BMDM as broken lines. Data are represented as mean ± SEM (*n* = 3 mice).

Bone‐marrow‐derived macrophages accumulate very high proportions of the C38:4 species of PI (Fig 4A). We followed the incorporation of ^13^C_3_‐glycerol units into the major PA and PI species in BMDM in analogous experiments to those described above for HEK293 cells (Fig 4B–F). The main difference we observed in BMDM compared with HEK293 was the much greater level, and less pronounced lag, in the ^13^C‐labelling of C38:4‐PI relative to other species (Figs 4B vs. 2E). Given the similarly low level of ^13^C‐labelling of C38:4‐PA in both cell types (Figs 4E and 2G), this suggested to us that BMDM may possess an enhanced capacity for acyl chain remodelling towards C38:4‐PI. To test this, we performed parallel ^13^C‐labelling experiments with BMDM prepared from WT and LPIAT1‐KO mice. BMDM lacking LPIAT1 possessed reduced proportions of C38:4‐PA and ‐PI at steady state (Fig 4A), enhanced ^13^C‐labelling of several PA species (Fig 4E, broken vs. solid lines), and dramatically reduced ^13^C‐labelling of C38:4‐PI relative to other species (Fig 4B vs. C). This is direct evidence that LPIAT1 is involved in acyl chain remodelling of PIs made from PAs synthesised *de novo*. LPIAT1‐KO macrophages also exhibited increased rates of ^13^C‐labelling of non‐C38:4 species of PI (note the differences in scale of the Y‐axes in Fig 4B vs. C), consistent with enhanced ^13^C‐labelling of their corresponding PA species, suggesting a compensatory effect of LPIAT1‐deletion on the *de novo* synthesis of non‐C38:4‐PI species. A very similar effect has been described recently in mouse liver lacking LPIAT1 (Tanaka *et al*, 2021).

In addition, we compared the rate of incorporation of ^18^O‐phosphate groups into both PA and PI in WT vs. LPIAT1‐KO BMDM. We observed similarly large and selective rates of formation of 38:4‐PI+4 (Fig 4G) in both genotypes, indicating LPIAT1‐KO macrophages can support substantial levels of C38:4‐PI synthesis from pre‐existing precursor pools. This provides a simple explanation for why there is only a small drop in the proportion of C38:4‐PI in the LIPIAT1‐KO (Fig 4A), despite a profound defect in acyl chain remodelling. We do note, however, that both the accumulation of C38:4‐PA+4 and the steady‐state level of C38:4‐PA are reduced in LIPIAT1‐KO macrophages (Fig 4A and H), suggesting incorporation of C20:4 chains into PI does make a contribution to the total cellular pool of C38:4‐PA in these cells.

### MCF7 cells are relatively poor at enriching for the C38:4 species of PI

**Figure 5:**
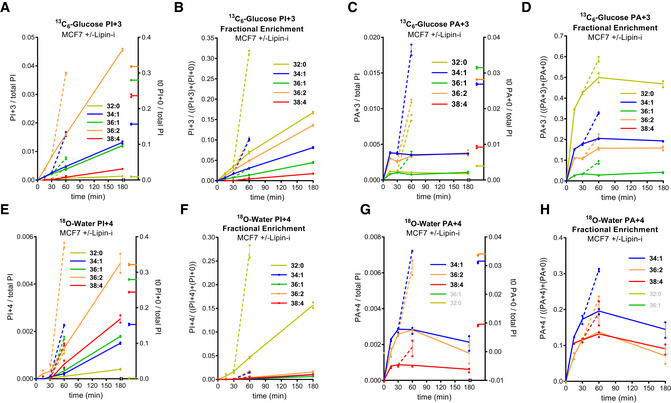
MCF7 cells are relatively poor at enriching for the C38:4 species of PI A–HLabelling of PI and PA in MCF7 cells incubated with ^13^C_6_‐glucose (A–D) or ^18^O‐water (E–H). Data labels are as described in the legend to Fig 2. Where indicated, 200 μM propranolol (Lipin‐i) was added at 30 min and incubations continued for a further 30 min (broken lines). Where the measurement of an individual species has been omitted for technical reasons, it is “greyed out” in the legend. The C38:4‐PA+3 signal was below the limit for quantification. Data are represented as individual points (*n* = 2 wells) with bisecting lines from a single experiment in which all three labelling strategies were performed in parallel, in equivalent media. The results are representative of three similar experiments where each of the labelling strategies were performed in slightly different media, two of which included the addition of propranolol.

We followed ^13^C_6_‐glucose and ^18^O‐water labelling of PA and PI species in MCF7 cells (Fig 5), which have much lower proportions of the C38:4 species at steady‐state (Fig 1A). Several clear differences were observed between MCF7 and HEK293. There was less ^13^C‐incorporated into C38:4‐PI (Figs 5A vs. 2E) and a much lower fractional enrichment of ^13^C in the faster turning over species (Figs 5B vs. 2F), despite more similar labelling (Figs 5C vs. 2G) and fractional enrichment (Figs 5D vs. 2H) in PA. Furthermore, there was slower and much lower incorporation of ^18^O‐phosphate into C38:4‐PA and PI (Figs 5E, F and G, H vs. 2J, K and L, M). There was also a much more pronounced effect of propranolol on ^18^O‐phosphate incorporation in the Mcf7 cells, including a clear stimulation of ^18^O‐phosphate incorporation into both C38:4 PA and PI (broken lines in Figs 5E and G vs. 2J and L), suggesting the pathway for PI synthesis reported by ^13^C_3_‐glycerol incorporation is a much bigger proportion of total PI synthesis in these cells. Overall, this suggests MCF7 cells have a lower capacity to generate C38:4‐PA and PI from pre‐existing DG pools and also a lower capacity for acyl chain remodelling of PIs made from PAs derived *de novo*.

### GPCRs stimulate PI synthesis from PAs derived via PLC, but exclude those derived from PLD

**Figure 6:**
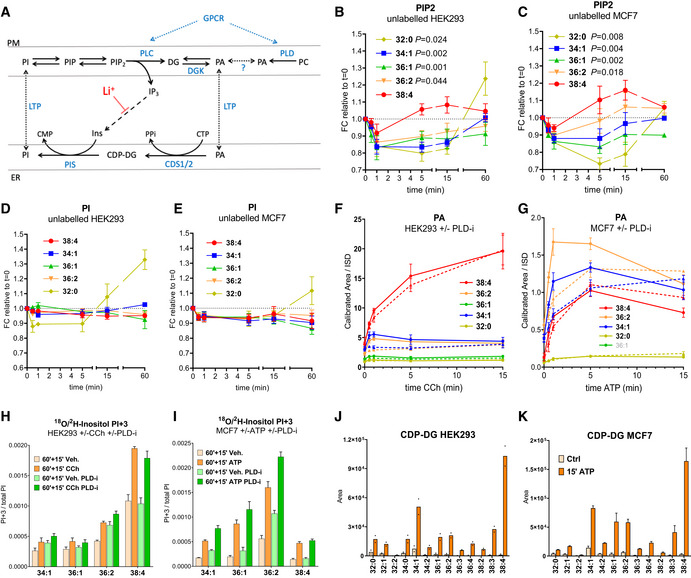
GPCRs stimulate PI synthesis from PAs derived via PLC, but exclude those derived from PLD AA schematic diagram illustrating the stimulated “PI cycle.”B–EThe fold changes in the levels of different acyl chain variants of PIP2 (B, C) and PI (D, E) in HEK293 cells stimulated with 100 μM carbachol (B and D) or MCF7 cells stimulated with 25 μM ATP (C and E). HEK293 data are represented as mean ± SEM from five independent experiments for PIP2 and three for PI. MCF7 data are represented as mean ± SEM from four independent experiments. For PIP2, the areas under the curve (AUC) from time 0 to 15 min were analysed with a one‐way ANOVA with the Geisser–Greenhouse correction followed by Dunnett's multiple comparisons test (adjusted *p*‐values vs. C38:4 are indicated next to the species' name).F, GThe formation of the indicated species of PA in HEK293 cells stimulated with with 100 μM carbachol (F), or MCF7 cells stimulated with 25 μM ATP (G), in the presence (broken lines) or absence (solid lines) of 5 μM ML299 (PLD‐i), added 15 min before stimulation. Where the measurement of an individual species has been omitted for technical reasons, it is “greyed out” in the legend.H, IThe formation of the PI+3 isotopologue in ^18^O/^2^H‐inositol‐labelled HEK293 cells incubated with 100 μM carbachol (CCh) or vehicle (Veh.) (H) or MCF7 cells incubated with 25 μM ATP or vehicle (Veh.) (I). Cells were pre‐labelled with ^18^O/^2^H‐inositol for 60 min before stimulation with CCh or ATP. Where indicated, ML299 or DMSO were added 15 min before stimulation with agonists. Data are normalised to the total level of all PI species to correct for differences in cell mass between biological replicates.J, KThe formation of CDP‐DG species in HEK293 incubated with 100 μM carbachol (CCh) or vehicle (Ctrl) (J), or MCF7 cells incubated with 25 μM ATP or vehicle (Ctrl) (K) for 15 min. Cells were incubated for 16 h under conditions of reduced inositol and in the presence of 10 mM LiCl for 30 min before the addition of agonist (see the Materials and Methods section). Data are for uncalibrated CDP‐DG species, represented as arbitrary units. Data information: Data in panels F–I are means ± SD (*n* ≥ 3) from single experiments, representative of at least 2 similar experiments; data in J are individual points with bisecting bars (*n* = 2 wells) from a single experiment representative of 2 similar experiments; data in K are means ± SEM of three independent experiments with duplicated treatments. See also Fig EV4.

**Figure EV4:**
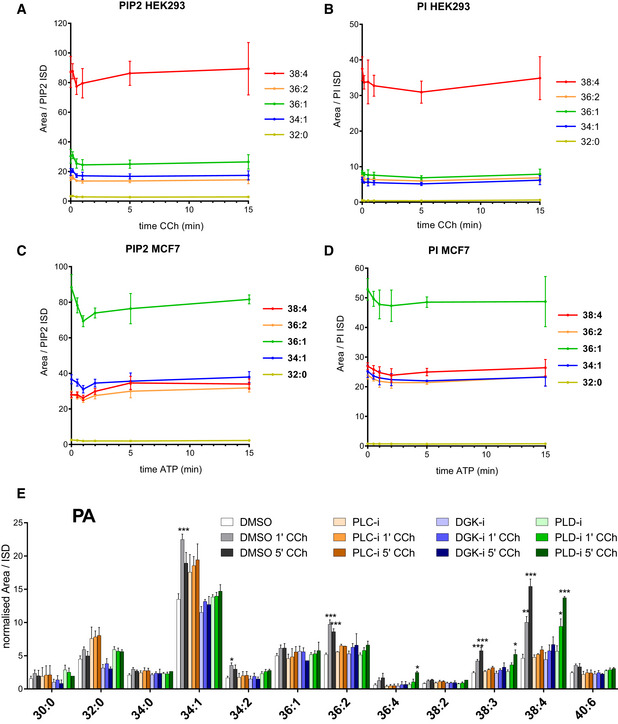
GPCR‐stimulated changes in phosphoinositide and PA levels A–DExample of a time‐course experiment to follow the changes in the selected species of PIP2 and PI after stimulation of HEK293 cells with 100 μM CCh or MCF7 cells with 25 μM ATP. Data are presented as mean ± SD (*n* = 3 wells/condition) of the uncalibrated response ratios vs. their ISD from a single experiment representative of 2 similar experiments.EAccumulation of PA species in HEK293 cells stimulated with 100 μM CCh. Prior to stimulation, cells were preincubated for 15 min with vehicle (DMSO) or the indicated inhibitors: 10 μM U73122 (PLC‐i), 30 μM R59022 (DGKi), 5 μM ML299 (PLD‐i). Data are represented as mean ± SEM, and was generated by pooling seven independent experiments, with each inhibitor tested in at least 3 of them. To compensate for differences between experiments in the response to CCh, the Area/ISD data (uncalibrated) was normalised relative to the total PA levels after 1 min CCh in the DMSO control. Log‐transformed data were analysed with a 2‐way ANOVA with Geisser–Greenhouse correction, followed by Dunnett's multiple comparisons tests (*P ≤ 0.05, **P ≤ 0.01, ***P ≤ 0.001 vs. the unstimulated DMSO control samples).

Several GPCRs are established to signal via both PLC‐catalysed hydrolysis of PI(4,5)P_2_ and PLD‐catalysed hydrolysis of PLD (Fig 6A) (Balla 2013; Scott *et al*, 2013; Brandenburg *et al*, 2014). We used our methods to measure the PA species generated via these two routes and also the extent to which they served as substrates for PI synthesis in muscarinic receptor‐stimulated HEK293 cells and purinergic receptor‐stimulated MCF7 cells. The stimulation of these receptors resulted in a small, transient drop in the total level of PIP2, with little effect on the total level of the much larger PI pool (Fig EV4A–D); this is a common pattern seen in cell lines stimulated by endogenous levels of receptors and reflects efficient replenishment of PIP2 during PLC stimulation (Stephens *et al*, 1993). There was, however, a clear change in the acyl chain composition of PIP2 and PI during stimulation; the C38:4 species of PIP2 recovered much faster than other species in carbachol‐stimulated HEK293 cells (Fig 6B) and PIP2 species recovered in the rank order C38:4 > C36:2 > C34:1 = C36:1 > C32:0 in ATP‐stimulated MCF7 cells (Fig 6C). Furthermore, there was an increase in the proportion of the C32:0 species of PI at later times in both cell types (Fig 6D and E).

Carbachol stimulation of HEK293 cells lead to the rapid accumulation of several PA species (Figs 6F and EV4E). The accumulation of some of these species was relatively transient and highly sensitive to inhibition of PLD (e.g. C34:1, C36:2; Figs 6F and EV4E). In contrast, the accumulation of C38:4‐PA was more sustained, sensitive to inhibition of PLC and DGK (Fig EV4E), but insensitive to inhibition of PLD (Figs 6F and EV4E). This suggests that most of the C38:4‐PA in carbachol‐stimulated cells is generated indirectly via PLC and DGK, and most of the C34:1‐PA is generated directly via PLD. This is consistent with the relatively high proportion of the C38:4 species of PIPn in these cells.

ATP‐stimulation of MCF7 cells also generated substantial increases in several species of PA, with some similarities and differences to the response measured in HEK293 cells (Fig 6G). The early phase of accumulation of C36:2‐PA and C34:1‐PA was partially sensitive to inhibition of PLD, but the accumulation of C38:4‐PA, and the accumulation of other PA species at later times, were insensitive to PLD inhibition (Fig 6G). This is consistent with the wider variety of acyl chain species of PIP2 available for PLC hydrolysis in MCF7 cells.

We also measured the effect of GPCR stimulation on ^18^O/^2^H‐inositol incorporation into PI. Carbachol stimulated rapid and substantial labelling of PI in HEK293 cells (Fig 6H). This incorporation was both selective for the C38:4 species of PI and insensitive to inhibition of PLD (Fig 6H), suggesting it was driven primarily by the activation of PLC. In MCF7, ATP also stimulated ^18^O/^2^H‐inositol incorporation into several PI species, and in each case that stimulated incorporation was not reduced by inhibition of PLD (Fig 6I). This data indicate that in both cell types activation of GPCRs stimulates PI synthesis from PAs derived via PLC, but PAs generated directly via PLD are excluded.

We attempted to confirm GPCR‐stimulated PI synthesis using the alternative approach of trapping the CDP‐DG intermediate through inositol depletion in the presence of Li^+^ (Godfrey 1989; Kim *et al*, 2015). Under these conditions, both carbachol and ATP stimulated very substantial accumulations of CDP‐DG species (Fig 6J and K). We noticed that there was a selective and large accumulation of the C38:4 species in both cell lines (Fig 6J and K). As MCF7 contained a much lower proportion of C38:4 in basal PIP2 (Fig EV4C) and stimulated PA (Fig 6G), this similar distribution of accumulated CDP‐DG species strongly suggested acyl chain selective recycling of PLC‐derived metabolites. However, the accumulation of CDP‐DG species did not align closely with the simulated incorporation of ^18^O/^2^H‐inositol (Fig 6I vs. K). There could be several potential explanations for this difference, including the confounding effects of the PIS exchange reaction, and so we chose to further investigate acyl chain selectivity in stimulated PI synthesis using both ^18^O‐water and ^13^C_6_‐glucose labelling.

### GPCRs stimulate PI synthesis via both an acyl chain‐selective recycling pathway and *de novo* synthesis

**Figure 7:**
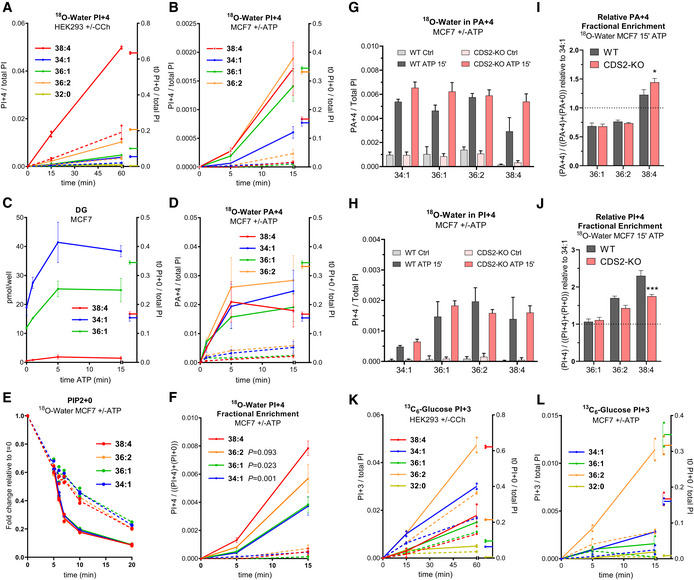
GPCRs stimulate PI synthesis via both an acyl chain‐selective recycling pathway and *de novo* synthesis AThe formation of the PI+4 isotopologue during the incubation of HEK293 cells with ^18^O‐water in the presence of 100 μM carbachol (solid lines) or vehicle (broken lines) added at *t* = 0. Data are represented as individual points with bisecting lines (*n* = 2) from a single experiment, representative of three similar experiments.BThe formation of the PI+4 isotopologue during the incubation of MCF7 cells with ^18^O‐water in the presence of 25 μM ATP (solid lines) or vehicle (broken lines) added at *t* = 0. Data are represented as mean ± SEM of four independent experiments.CThe formation of the indicated DG species in MCF7 cells stimulated with 25 μM ATP added at *t* = 0. For comparison, the steady‐date levels of the unlabelled PI species at *t* = 0 are shown on the right axis. Data are represented as mean ± SEM of three independent experiments.DThe formation of the PA+4 isotopologue during the incubation of MCF7 cells with ^18^O‐water in the presence of 25 μM ATP (solid lines) or vehicle (broken lines) added at *t* = 0. Data are represented as mean ± SEM of four independent experiments.EFold changes in the indicated PIP2+0 isotopologues during the incubation of MCF7 cells with ^18^O‐water in the presence (solid lines) or absence (broken lines) of 25 μM ATP added at *t* = 5 min. Data are represented as individual points with bisecting lines (*n* = 2) from a single experiment, representative of three similar experiments.FThe fractional enrichment of the PI+4 isotopologue during the incubation of MCF7 cells with ^18^O‐water in the presence of 25 μM ATP (solid lines) or vehicle (broken lines) added at *t* = 0. Data are represented as mean ± SEM from four independent experiments. The AUCs from time 0 to 15 min were analysed with a one‐way ANOVA with the Geisser–Greenhouse correction, followed by Dunnett's multiple comparisons test (adjusted *P*‐values vs. C38:4 are indicated next to the species' name).G, HFormation of the indicated PA+4 and PI+4 isotopologues in WT or CDS2‐KO MCF7 clones incubated with ^18^O‐water in the presence or absence of 25 μM ATP for 15 min. Data are means ± SEM from a typical experiment using independently derived clones (WT *n* = 3, CDS2‐KO *n* = 5).I, JThe fractional enrichments for the indicated PA+4 and PI+4 isotopologues normalised to that of C34:1; this was done to minimise clonal variation and to visualise the enrichment in the C38:4 species vs. other species. Data were pooled from independent experiments (*n* = 3) using multiple clones (WT *n* = 4, CDS2‐KO *n* = 5). A two‐way ANOVA followed by Sidak's multiple comparisons correction was used to test the differences between genotypes (*P ≤ 0.05 vs. WT, ***P ≤ 0.001 vs. WT).KThe formation of the indicated PI+3 isotopologues during the incubation of HEK293 cells with ^13^C_6_‐glucose in the presence of 100 μM carbachol (solid lines) or vehicle (broken lines) added at *t* = 0. Data are represented as individual points with bisecting lines (*n* = 2) from a single experiment, representative of 3 similar experiments.LThe formation of the indicated PI+3 isotopologues during the incubation of MCF7 cells with ^13^C_6_‐glucose in the presence of 25 μM ATP (solid lines) or vehicle (broken lines) added at *t* = 0. The C38:4‐PI+3 signal was below the limit for quantification. Data are represented as individual points with bisecting lines (*n* = 2) from a single experiment, representative of 2 similar experiments. Data information: See also Fig EV5.

**Figure EV5:**
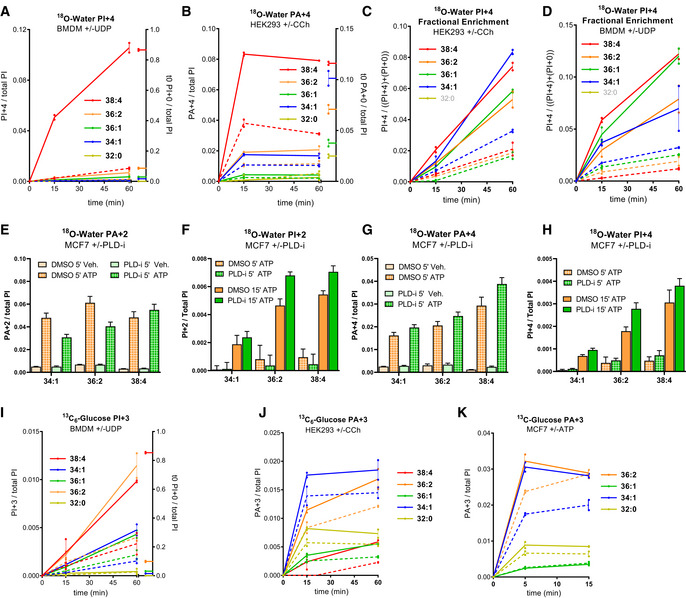
GPCRs stimulate acyl chain selective synthesis of PI A–DIncorporation ^18^O‐water in the indicated PI+4 or PA+4 isotopologues in BMDMs or HEK293 in the presence (solid lines) or absence (broken lines) of 100 μM UDP (BMDMs) or 100 μM carbachol (HEK293). Data labels are as described in the legend to Fig 2. Data are represented as individual points and bisecting lines (*n* = 2) from a single experiment, representative of three similar experiments.E–HIncorporation ^18^O‐water in the indicated isotopologues of PA and PI in stimulated MCF7 cells with or without a PLD inhibitor. Cells were pretreated with 5 μM ML299 (PLDi) or vehicle (DMSO) for 15 min before the incubation with ^18^O‐water. After a 5 min pre‐labelling, cells were treated with 25 μM ATP or vehicle and incubated for a further 5 min or 15 min before quenching with 1 M HCl. Data are represented as mean ± SD (5 min *n* = 4, 15 min *n* = 3) from a single experiment, representative of three similar experiments.I–KThe formation of the indicated PI and PA isotopologues during the incubation of BMDMs (I), HEK293 (J) and MCF7 (K) cells with ^13^C_6_‐glucose in the presence (solid lines) or absence (broken lines) of 100 μM UDP (BMDMs), 100 μM carbachol (HEK293) or 25 μM ATP (MCF7) added at *t* = 0. Values of individual isotopologues are normalised to the total level of all PI species, to correct for differences in cell mass (for comparison, in panel (I) the steady‐date levels of the unlabelled PI species at *t* = 0 are shown on the right axis). Data are represented as individual points and bisecting lines (*n* = 2) from a single experiment, representative of 3 similar experiments.

GPCRs stimulated very large fold increases in the accumulation of the +4 isotopologue of PI in HEK293 cells (Fig 7A), MCF7 cells (Fig 7B) and BMDMs (Fig EV5A) when these cells were incubated simultaneously with agonist and ^18^O‐water, indicating this is a very sensitive method for detecting the initial, stimulated rates of formation of these molecules. In both HEK293 and BMDMs, there was substantial selectivity for the formation of C38:4‐PI+4, indicating that the high enrichment for this species in the starting PIPn pool in these cells is retained during GPCR‐stimulated synthesis of new PIs; this is confirmed by the similar increases in fractional enrichment of the different PI+4 acyl chain species (Fig EV5C and D). This suggests PA species derived via PLC activation in these cells are effectively channelled into new PI synthesis, although the extent to which this is simply driven by a mass action effect of these PAs dominating the cellular PA pool (see for example Figs 6F and EV5B) is difficult to discern.

MCF7 cells possess a wider variety of acyl chain species of PIPn and the decline in the starting levels of the PIP2+0 isotopologue in these ^18^O‐water labelling experiments gives an estimate of their rate of consumption. In unstimulated cells, PIP2+0 species declined with a t_1/2_ of a few minutes (Fig 7E, broken lines), consistent with previous work indicating that the phosphomonoester phosphates of PI(4,5)P_2_ are turned over rapidly in cells (King *et al*, 1989). Stimulation with ATP accelerated this decline, presumably via the activation of PLC (Fig 7E, solid lines). Each of the PIP2+0 acyl chain species showed a remarkably equivalent rate of loss, under both basal and stimulated conditions, indicating there is no acyl chain selectivity in the major phosphomonoesterases or PLCs acting on these pools (Fig 7E). Therefore, MCF7 cells afforded an opportunity to interrogate whether the variety of PA species produced via activation of PLC are equivalently converted into new PIs.

We measured the ATP‐stimulated formation of the major isotopologues of PA in MCF7 cells incubated with ^18^O‐water. Initially, ^18^O‐nuclei derived from ^18^O‐water would be expected to incorporate randomly into the nucleotide‐derived phosphate groups of PA and PI, leading to the generation of +2/+4/+6 or +2/+4 isotopologues, respectively. However, PLD‐catalysed cleavage of PC would be expected to generate PA with only a single ^18^O‐nucleus (through nucleophilic attack of a single water molecule). ATP stimulated the formation of several isotopologues of PA (Figs 7D and EV5E and G), in agreement with the formation of several species of PA in the analogous unlabelled experiments (Fig 6G). The formation of C34:1‐ and C36:2‐PA+2 was selectively and partially reduced by inclusion of a PLD inhibitor (Fig EV5E), consistent with our previous conclusion that these species are derived, in part, via PLD. In contrast, C38:4‐PA+2 and all PA+4 species were insensitive to PLD inhibition (Fig EV5E and G), indicating they were derived via PLC/DGK.

These results suggest that synthesis of PA+4 and PI+4 in these studies is a good measure of the initial rates of synthesis of PA and PI derived via the stimulation of PLC. To help further define any potential acyl chain selectivity in PA synthesis, we also measured the production of selected species of DG in response to ATP (Fig 7C). A comparison between these measures should pinpoint steps which are acyl chain selective. This analysis is complicated, however, by an incomplete assessment of all the relevant acyl chain species in each of the different assays (because of our technical limitations), the precursor‐product relationship of PA in this pathway (which means acyl chain selectivity in the pathways for either its formation and/or consumption could lead to differences in its composition) and the potential for PA to be synthesised by different pathways (significant synthesis by PLD has been ruled out, but *de novo* synthesis could still contribute to measurement of PA+4, see Fig 2I). Nevertheless, a simple comparison between the starting distributions of acyl chain species in PIP2 with the formation of selected species of DG identifies the C38:4 species as clearly under‐represented (Fig 7C). Given that all species of DG should be generated at an equivalent proportional rate on PLC‐stimulation (Fig 7E), this indicates the C38:4 species of DG is selectively phosphorylated to PA by DGKs (this is illustrated by comparing the very similar starting proportions of C34:1 and C38:4 species in PIPn with the huge difference in the accumulation of DGs derived from them). This conclusion is supported by the over‐representation of C38:4‐PA+4 synthesis on stimulation (Fig 7D), although this measure may under‐represent the true level of selectivity in PA synthesis due to further acyl chain selection in its conversion to CDP‐DG (supported by our CDP‐DG accumulation data—see Fig 6K). However, notwithstanding the difficulties in apportioning C38:4 selectivity in the steps leading to the synthesis and consumption of PA, overall acyl chain selectivity in the pathway for stimulated PI synthesis is clearly demonstrated by the fractional accumulation of label in PI itself (Fig 7F), which is a direct reflection of the relative rates of formation of the different PI species compared to their starting proportions in the PIPn pool. This data indicate there is substantial acyl chain selectivity in new PI synthesis in MCF7 cells stimulated with ATP in the rank order C38:4 ≥ C36:2 > C36:1 = C34:1 (Fig 7F).

To further investigate the possibility that there is acyl chain selection in the conversion of PA to CDP‐DG in stimulated MCF7 cells, we also performed parallel ^18^O‐water‐labelling experiments in independent clones of WT and CDS2‐KO cells. CDS2‐KO cells were able to support substantial ATP‐simulated accumulations of PA+4 (Fig 7G) and PI+4 (Fig 7H), but we did observe a significant, selective increase in the fractional enrichment of C38:4‐PA+4 (Fig 7I) and a decrease in the fractional enrichment of C38:4‐PI+4 (Fig 7J), compared with the WT. This suggests CDS2 also makes a contribution to the selective recycling of C38:4 backbones during stimulation with ATP.

The ^18^O‐water‐labelling experiments discussed above measure PI synthesis from all PAs with newly incorporated phosphate groups, although there will be differences in the relative specific activities of PAs made via DGK vs. those derived directly via glycolysis. To directly measure the effect of GPCR stimulation on PI synthesis from PAs made *de novo,* we turned to our ^13^C_6_‐glucose labelling strategy. GPCRs stimulated the accumulation of +3 isotopologues of PI in HEK293 cells (Fig 7K), MCF7 cells (Fig 7L) and BMDMs (Fig EV5I) when they were incubated simultaneously with agonist and ^13^C_6_‐glucose. The fold increases on stimulation were substantial, greatest for the MCF7 cells, but less than those seen for the analogous ^18^O‐water‐labelling measurements made in parallel. The relative rates of accumulation of the different acyl chain versions of the PI+3 isotopologues were, for each cell, in good agreement with our previous observations for the labelling of these species in basal cells (see sections above). A cross comparison between the ^13^C_6_‐glucose and ^18^O‐water‐labelling experiments indicates the pathway measured by ^13^C_6_‐glucose labelling makes a minor contribution to total, stimulated PI synthesis in HEK293 cells and BMDMs, but a much larger contribution in the MCF7 cells. Furthermore, in MCF7 cells, the variety of species generated by this pathway must partially obscure the C38:4‐selectivity of the pathway for PI synthesis that utilises PAs derived from PLC. This would also explain the wider variety of PI species detected in the ^18^O/^2^H‐inositol labelling experiments in ATP‐stimulated MCF7 cells (see section above). The stimulation of the *de novo* pathway for PI synthesis would also explain the increases in species characteristic of this pathway at longer times of stimulation (e.g. C32:0, Fig 6D and E). The point(s) at which GPCRs stimulate *de novo* PI synthesis is not revealed by these experiments, but the greater fold stimulation of ^13^C_6_‐glucose incorporation into PI vs. PA (Figs 7K vs. EV5J and Figs 7L vs. EV5K) suggests an effect at the CDS or PIS steps.

## Discussion

We present improved and extended LC–MS methods for measuring different acyl chain variants of PI, PA and CDP‐DG. We used these methods together with an isotopologue tracing strategy to measure the initial rates of formation of key molecules in the PI synthesis pathway; we used an established approach with ^13^C_6_‐glucose to trace the incorporation of glycerol units into PA and PIPn; we synthesised an isotopologue of inositol (^18^O/^2^H‐inositol) and used this to measure the incorporation of inositol into PI; and we developed a new approach using ^18^O‐water (H_2_^18^O) to trace the incorporation of phosphate groups into PA and PIPn. ^18^O‐water is readily available and equilibrates very rapidly across cell membranes and into cellular nucleotide pools, indicating it should be a useful approach for tracing the incorporation of phosphate into many other small molecules and post‐translational modifications eg protein kinase‐mediated phosphorylation of proteins.

**Figure 8:**
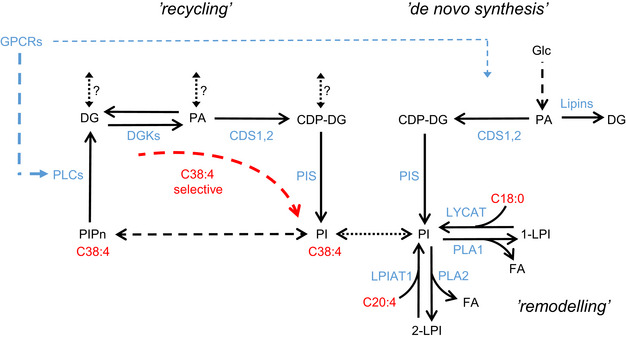
The organisation of PIPn synthesis in mammalian cells

These approaches proved highly complementary and revealed distinct pathways for PI synthesis. One pathway, which we term the “*de novo synthesis‐remodelling pathway*” utilises PAs derived via glycolysis to generate PIs which then undergo rapid acyl chain remodelling towards C38:4‐PI. A separate pathway, which we term the “*recycling pathway*” utilises pre‐existing precursors to selectively synthesise C38:4‐PA and ‐PI (see Fig 8).

Our studies do not reveal the order or kinetics of the processes by which the initial PIs generated by the *de novo* synthesis pathway undergo subsequent acyl chain remodelling, but LPIAT1 is clearly a major player in remodelling towards C38:4‐PI in BMDM. Fully resolving these pathways will be a difficult task, likely requiring the use of several acyl chain tracers and systematic deletion of acyl‐CoA transferases (Shindou *et al*, 2009). Our gene deletion studies indicated both CDS1 and CDS2 can utilise PAs synthesised *de novo*, but CDS1 appears to be more important for the synthesis of some species of PI (e.g. C32:0‐PI), consistent with previous reports that this isoform may have a wider substrate preference (D'Souza *et al*, 2014). Our data also indicate that the PAs and PIs created by the *de novo* synthesis pathway are highly sensitive to inhibition of the PA‐phosphatase activity of lipins, consistent with the role of these enzymes as “gate keepers” in directing the flux between the DG and CDP‐DG arms of *de novo* lipid synthesis (Reue & Wang, 2019).

The *recycling pathway* is able to selectively generate C38:4‐PA and PI, even under basal conditions. Furthermore, the lack of any lag in our ^18^O‐water ‐labelling studies, together with the lack of effect of deleting LPIAT1, suggest strongly that acyl chain remodelling is not a major factor in the recycling pathway. The precise organisation and location of the *de novo* synthesis, remodelling and recycling pathways are currently unknown, but it seems likely the *de novo* synthesis and remodelling pathways are closely coupled and separated in some way from the recycling pathway. In this regard, it is interesting that LYCAT and PIS have been found to closely co‐localise in an ER‐derived vesicular compartment (Bone *et al*, 2017). It is also clear that the activities of the *de novo synthesis‐remodelling* and *recycling* pathways vary greatly between the three cell models we chose to study. Our water labelling studies show HEK293 and BMDMs cells have a very active *recycling pathway*, whereas this pathway is much less active in MCF7 cells. Furthermore, our glucose labelling studies indicate MCF7 cells are much less active in acyl chain remodelling within their *de novo synthesis‐remodelling* pathway. It is tempting to speculate that the inability of MCF7 cells to enrich for C38:4‐PI may partly explain why they have a less active, C38:4‐dependent recycling pathway, but further work is needed to discover the molecular basis of their diminished capacity to accumulate and/or use C20:4‐ or C18:0‐CoAs to remodel their PIs. In this regard, current evidence points to the likely involvement of both environmental and genetic factors in determining the acyl chain composition of PIPn in different cell types. We and others have noted previously that some cell lines appear to be limited by the levels of C20:4 (arachidonate) in their media (Rouzer *et al*, 2006; Anderson *et al*, 2016), and we have also noticed that the proportions of some species (e.g. C36:1‐PI in BMDM) can vary significantly depending upon the batch of FBS used in their culture. However, the clear difference in PI composition amongst similar cell types grown under identical conditions (Fig EV1E) indicates genetic/epigenetic factors must also play a big part.

We show that acute stimulation of PLC by GPCRs leads to a substantial increase in PI synthesis. At shorter times, our ^18^O‐water labelling studies indicate this increase in PI synthesis is driven through some version of the *recycling pathway*, although how this relates to the recycling pathway that operates under basal conditions is unclear (our “basal” cells were grown in serum, which could simply reflect reduced levels of “stimulation”). This stimulated recycling pathway is equivalent to the “PI cycle” invoked over 45 years ago to explain stimulated incorporation of radioactive tracers into PI (Michell 1975). The simplest explanation for the stimulation of PI synthesis by GPCRs is that large fold increases in the generation of PLC‐derived DGs drive subsequent increases in PA, CDP‐DG and PI through mass action. Furthermore, the recent discovery of specialised ER‐PM contact sites, that form on stimulation and which allow lipid‐transfer‐proteins to counter‐exchange DG/PA and PI across these two membranes, offers a natural organisation for this pathway, its potential segregation from *de novo* synthesis, and the replenishment of plasma membrane PI and PIP2 pools without a global increase in cellular PI (Chang & Liou, 2015; Kim *et al*, 2015; Cockcroft & Raghu, 2018).

Somewhat surprisingly, our ^13^C_6_‐glucose labelling studies indicate *de novo* PI synthesis can also be stimulated by GPCRs, but the relative contributions the *recycling versus de novo synthesis‐remodelling* pathways make to new PI synthesis under these conditions is, again, very different between our cell models, with the *de novo* pathway playing a much bigger relative role in the ATP‐stimulated MCF7 cells. This is perhaps unsurprising given the relatively weak “basal” *recycling pathway* in these cells.

The GPCR‐stimulated *recycling pathway* can clearly discriminate between PAs generated by PLC and PLD, ignoring the later. Previous work has shown stimulated PLC and PLD produce different species of DG, with only the former acting as signalling molecules (Pettitt *et al*, 1997). In carbachol‐stimulated HEK293 cells, PLC and PLD generate significantly different acyl chain complements of PA (because of substantial differences in their PIP2 and PC compositions), but in ATP‐stimulated MCF7 cells, there is much more of an overlap, suggesting acyl chain discrimination alone cannot account for the very clear exclusion of PLD‐derived PAs from PI synthesis. It is tempting to speculate that there is also membrane sub‐domain segregation between PLC‐containing ER‐PM contact sites and PLD‐containing endocytic structures (Brandenburg *et al*, 2014; Bruntz *et al*, 2014), but there is no clear evidence for this or for how diffusion barriers would prevent mixing of the relevant lipids and enzymes.

The faster recovery of the C38:4 species of PIP2 after stimulation of PLC suggests this acyl chain configuration is favoured in a stimulated PI cycle. Indeed, two complementary approaches, ^18^O‐water labelling and CDP‐DG accumulation in the presence of lithium, indicate substantial acyl chain selectivity occurs in the GPCR‐stimulated recycling pathway, with a preference for the C38:4 species. Such preference is also observed in basal ^18^O‐water labelling, suggesting a mechanism to preserve or indeed enhance the C38:4 enrichment achieved by acyl chain remodelling of *de novo* PI's. The molecular basis for this selectivity is not yet clear, but several pieces of evidence point to a complex picture. The remarkably selective synthesis of C38:4‐PA in HEK293 and BMDM cultured under steady‐state conditions implicates the selective presentation or phosphorylation of different DGs by DGKs (through, for example, acyl chain selectivity in lipid transport, basal PLC activity or the DGKs themselves). This was supported by the effects of broad‐range DGK‐inhibitors, which substantially reduced water‐labelling of C38:4‐PA and ‐PI, with much smaller effects on other acyl chain species. Ten different isoforms of DGK are expressed in mammals (Cai *et al*, 2009; Ware *et al*, 2020), and recent evidence suggests acyl chains may be relevant to their function (de Turco *et al*, 2001; Sakane *et al*, 2018; Ware *et al*, 2020). We therefore attempted to implicate a specific isoform of DGK in the C38:4‐selective phosphorylation of DAG in HEK293 cells by using siRNA‐mediated knockdown, but did not obtain a conclusive answer; we did not observe a specific decrease in water‐labelling of C38:4‐PA or ‐PI with any specific siRNA, with reductions in mRNA levels ranging between 60 and 95%. The lack of effect in knocking‐down the expression of the DGKε isoform was particularly surprising given its clear selectivity for C38:4‐DG *in vitro* (Tang *et al*, 1996; Lung *et al*, 2009; Ware *et al*, 2020), but consistent with the minor effects of overexpressing this isoform on endogenous levels of C38:4‐DG in HEK293 cells (Ware *et al*, 2020). This data suggest C38:4‐selectivity may be a property of multiple DGK isoforms or that C38:4‐DG dominates the DG pool at a specific location, for example the plasma membrane.

Evidence that location is not the primary factor comes from our observations with GPCR‐stimulated MCF7 cells. ATP‐stimulation of PLC produces different acyl chain species of DG at precisely the same time and place and yet these are clearly channelled at different rates towards PI. Furthermore, our measurements of stimulated DG formation and water‐labelling of PA confirm substantial C38:4‐selectivity occurs at the DGK step under these conditions. In addition, we also observed a partial decrease in C38:4‐selective PI synthesis in CDS2‐KO cells, which suggests some acyl chain selectivity also occurs in the conversion of PA to CDP‐DG. Thus, overall, our data support the idea that several enzymes in the “PI cycle” must act cooperatively to enrich the C38:4 species PIPn, an idea originally proposed by Epand and colleagues based on *in vitro* enzyme assays (D'Souza & Epand, 2014). In this regard, it is interesting to note that CDS1/2 has recently been shown to bind to AGPAT2 (an acyl transferase acting on LPA) to facilitate metabolic channelling between PA and PI in the *de novo* synthesis pathway (Mak *et al*, 2021), and thus, it seems highly plausible that an analogous “scaffolding” arrangement may operate between multiple enzymes in the stimulated PI recycling pathway.

The organisation of PIPn synthesis in mammalian cells is necessarily complex, involving several membrane compartments and several shared metabolic intermediates. The presence of a *de novo synthesis‐remodelling pathway* that delivers the nett synthesis of a particular acyl chain complement of PI, alongside a separate acyl chain‐selective *recycling pathway,* allows this acyl chain complement to be retained without re‐engineering it every time the PI head‐group is separated from the DG backbone, that is via the actions of PLC or PLD. This is particularly important for the close coupling of PIP2 supply and demand under dramatically altered conditions (e.g. activation of PLC by receptors), without which both PIP2 function and that of the entire PIPn pool would be severely compromised. Furthermore, acyl chain selectivity in the resynthesis pathway allows increased opportunities to discriminate between different pools of DG and PA beyond spatial segregation alone, allowing these pools to function separately as metabolic intermediates (e.g. DG and PA destined for lipid synthesis, including PI) or signalling molecules (e.g. DG in the regulation of PKC or, PA in the regulation of endocytosis) (Nadler *et al*, 2013; Kamiya *et al*, 2016; Sakane *et al*, 2018; Schuhmacher *et al*, 2020). The phenotypes of genetic polymorphisms and knock‐outs in LPIAT1 (Lee *et al*, 2012; Luukkonen *et al*, 2016; Mancina *et al*, 2016; Thabet *et al*, 2016; Tanaka *et al*, 2021) suggest PIPn acyl chain composition is physiologically important, but do not identify the underlying molecular mechanisms at play. Furthermore, the evolutionary pressures leading to the enrichment of the particular saturated and unsaturated acyl chains that are enriched in the sn‐1 and sn‐2 positions, respectively, are not known and several alternative theories have been proposed for the selective properties it may convey, including its influence on the recognition of PIPn by enzymes and effectors (Epand 2017; Antonescu *et al*, 2019; Barneda *et al*, 2019; Bryant *et al*, 2021). Our data suggest the contribution of the acyl chain composition to an efficient or “closed” PI cycle should be added to this list. Furthermore, we would argue that the well‐documented deficiencies in acyl chain availability in certain culture media or tumour microenvironments, particularly of unsaturated fatty acids or their essential precursors, should be investigated for their potential to influence chronic PLC signalling. Moreover, it seems likely that the paradigm for acyl chain selection described here will also apply to metabolic channelling within other major lipid classes with characteristic acyl chain compositions.

## Materials and Methods

[^13^C_6_, 99%]‐Glucose and [^18^O, 97%]‐Water were from Cambridge Isotopes. All chemicals/solvents for lipid analysis were AR grade. M‐CSF was from PeproTech, PLD‐i ML299 and DGK‐I R59949 were from Cayman Chemical, DGK‐i R59022 and PLC‐i U73122 were from TOCRIS. Dimethyl sulfoxide (DMSO), Adenosine 5′‐triphosphate (ATP), Uridine 5′‐diphosphate (UDP), Carbamylcholine (Carbachol, CCh), Lipin‐i (±)‐Propranolol, myo‐inositol, D‐Glucose, HEPES and 2X DMEM were from Sigma. L‐Glutamine, heat‐inactivated foetal bovine serum, dialysed foetal bovine serum, Penicillin–Streptomycin, Trypsin–EDTA (0.05%), DMEM/F12, RPMI and DMEM without glucose were from Gibco. Inositol‐free DMEM/F12 was from BioConcept.

The following lipids were purchased from Avanti Polar Lipids Inc:

1‐stearoyl‐2‐arachidonoyl‐sn‐glycero‐3‐phosphate.
1‐palmitoyl‐2‐oleoyl‐sn‐glycero‐3‐phosphoinositol.
1‐palmitoyl‐2‐palmitoyl‐sn‐glycero‐3‐phosphoinositol.
1‐stearoyl‐2‐arachidonoyl‐sn‐glycero‐3‐phosphoinositol.
1,2‐dipalmitoyl‐sn‐glycero‐3‐(cytidine diphosphate).
1‐oleoyl‐2‐heptadecanoyl ‐sn‐glycero‐3‐(cytidine diphosphate).

Lipids which were not commercially available were synthesised by the Biological Chemistry Facility at the Babraham Institute using a range of published methods (Kubiak & Bruzik, 2003; Conway *et al*, 2010). All lipids, whether purchased or synthesised in house, were analysed for purity by HPLC‐MS and TLC. The lipids were then quantified by analysis of the fatty acid content by GC–MS/MS.

The synthesis of ^18^O/^2^H inositol was carried out using a Ferrier rearrangement according to (Bender & Budhu, 1991) in H_2_^18^O followed by a reduction with sodium triacetoxyborodeuteride (see Appendix Fig S3 for details). The methodology was optimised as far as possible to maximise the yield of the correct isomer and isotope incorporation. The inositol isomer was checked by comparison with unlabelled inositols by GC–MS/MS using established methods (Kersting *et al*, 2003) and the relative abundance of the +3 isomer (^18^O ^2^H) determined as 24.6%.

### Cell culture

All cell cultures were maintained at 37°C with 5% CO_2_. HEK293 and MCF7 cells were, respectively, cultured in DMEM/F12 and RPMI‐1640 media, in both cases supplemented with 10% foetal bovine serum, and 1% w/v penicillin/streptomycin. MCF10a cells were cultured in DMEM/F12 supplemented with 5% horse serum, 10 ng/ml EGF, 10 μg/ml insulin, 0.1 μg/ml cholera toxin, 0.5 μg/ml hydrocortisone, 1% w/v penicillin/streptomycin. 2 days before lipid extraction, horse serum was substituted for foetal bovine serum. Bone marrow‐derived macrophages (BMDM) were prepared from C57BL/6J mice of 8–16 weeks of age as previously described (Houslay *et al*, 2016), and cultured in DMEM/F12 supplemented with 10% foetal bovine serum, 20 ng/ml M‐CSF and 1% w/v penicillin/streptomycin. LPIAT‐KO and their matching WT BMDMs were derived from frozen stocks of a previously described mouse model (Anderson *et al*, 2013). Cells were routinely seeded in 24, 12 or 6‐well plates and cultured for 16–24 h to achieve a 50–80% confluence at the time of the experiment.

### siRNA

The indicated genes were silenced in HEK293 cells using the ON‐TARGETplus SMARTpool mixture of 4 siRNA per gene (Horizon). Cells were seeded at 50,000 cells/well in 24‐well plates and incubated overnight in DMEM/F12 with 10% FBS without antibiotics. After renewing the media, cells were transfected with 20 pmol/well of siRNA and 1 μl/well of Lipofectamine RNAiMAX (ThermoFisher) diluted in OptiMEM following the manufacturer's instructions. Cells were cultured for 48 h before performing the labelling experiments and collecting RNA from parallel samples to validate the knockdowns. Media was renewed every 24 h, and cells were 80–90% confluent at the time of the assay.

### Generation of CDS1 and CDS2 KO cell lines

CDS2 and CDS1 KO cell lines were generated by CRISPR‐Cas9 using sgRNAs designed with the WGE‐CRISPR design tool (https://www.sanger.ac.uk/tool/wge/) and cloned into all‐in‐one pSpCas9(BB)‐2A‐GFP plasmid (Ran *et al*, 2013). The exon 3 of CDS1 was targeted with sgRNA 5’‐GAGGATCCCATATAGATGATC, while 5′‐ GTATTTACTGAGAATCCGCAA was directed against exon 5 of CDS2. One day after transfection with lipofectamine3000, GFP‐positive cells were FACS sorted and seeded at single cell per well in 96‐well plates in a 1:1 mix of fresh and conditioned medium. Clones were expanded, and screened by PCR‐amplification of the target DNA. The CDS1 site was amplified with the primer pair FW 5’‐TATCTCCCAGTGTGTGAATGAATGC, RV 5’‐AAATATGCTTGGCACAATGTCAG, and screened by loss of the BclI restriction site, whereas amplification with FW 5′‐ AAGCAGGATGAGGCGTGTTT and RV 5′‐ GAGCATCAGCTCTGCTCCC, followed by AciI digestion was used to detect modifications in the CDS2 site. Frameshift indels in the selected clones were identified by Sanger sequencing followed by TIDE (https://tide.nki.nl/) and ICE (https://ice.synthego.com//#/) analysis. These were further confirmed by NGS (Amplicon‐EZ) by Genewiz using barcoded primers to differentiate the selected clones. FASTAQ files were analysed by CRISPressoV2 (https://crispresso.pinellolab.partners.org/submission). CDS1 KO could not be established in MCF7 cells, as only six viable clones could be expanded and all of them presented indels that maintained the reading frame. See Appendix Fig S4.

### Western blot

A specific Western blot protocol was designed for the detection of the highly hydrophobic CDS2 protein, which tended to form aggregates after boiling the lysate. Cells were lysed in 1× Laemmli buffer (62.5 mM Tris base pH 6.8, 2% SDS, 10% glycerol, 5% 2‐mercaptoethanol and 0.01% bromophenol blue) and immediately loaded into SDS‐PAGE gel without boiling. Gels were briefly washed in a modified transfer buffer (25 mM Tris base, 192 mM glycine, 10% methanol and 0.01% SDS) before wet transference to PVDF membranes. Membranes were blocked with TBS (40 mM Tris/HCl, pH 8.0, 22°C; 0.14 M, NaCl) containing 0.1% v/v Tween 20 (TBS‐T) and 5% w/v non‐fat dry milk for 1 h. The membranes were incubated overnight at 4°C with the hCDS2 monoclonal antibody 2B9 (Novus Biologicals H00008760‐M01) at 6 μg/ml in TBS‐T with milk, washed with TBS‐T and probed with HRP‐conjugated secondary antibodies before developing with the ECL system and visualisation on X‐ray films. A similar protocol with different CDS1 antibodies could detect the overexpressed CDS1 but not its endogenous forms in our cell models.

### Quantitative PCR

RNA from CRISPR clones or siRNA experiments was extracted using TRI reagent (Sigma) and reverse transcribed to cDNA using SuperScript IV VILO (Thermofisher). Real‐time PCR was performed on an ABI system using the SYBR green‐AmpliTaq Gold master mix (Thermofisher). Gene expression levels were normalised to HPRT mRNA. The following primer pairs were used: HPRT FW 5’‐AAGCTTGCTGGTGAAAAGGA and RV 5′‐ GAACTCTCATCTTAGGCTTT; CDS1 FW 5′‐ GTGTTTGGATTCATTGCTGCCT and RV 5’‐AGGGCTCACATTCTGTCACG; CDS2 FW 5’‐CAGTCAGTCATTGGCTGGAAAA and RV 5’ ATCCTCCAAAGGGGCCAATG; DGKA FW 5’‐AATACCTGGATTGGGATGTGTC and RV 5’‐GTCCGTCGTCCTTCAGAGTC; DGKD FW 5’‐TGAATCCAGTACCAAAAACGTCA and RV 5’‐TTATCAGCACACAAGATGAGCTT; DGKE FW 5’‐GCTTCCAGTGCAAGGAGATT and RV 5’‐GGCACCAAATGCACCTGTAAT; DGKH FW 5’‐GATGCACAACTGGTACGCCT and RV 5’‐GGCCAGGGTAGTCCATTTACA; DGKQ FW 5’ TGAGCCAGACGCGGTTCTA and RV 5’‐CAGGCAACGTCCAACACTAC; DGKZ FW 5’‐CCGCTTTCGGAATAAGATGTTC and RV 5’‐AACAACACACTGGGGTTTCAG.

### Metabolic labelling

Three stable isotope‐labelled metabolites were used to trace PI synthesis: ^18^O/^2^H‐inositol, ^13^C_6_‐glucose and ^18^O‐water (Fig 1E). For inositol labelling cells were incubated in inositol‐free DMEM/F12 supplemented with 20 mM HEPES, 10% dialysed FBS and 50 μg/ml ^18^O/^2^H‐Inositol. Glucose labelling was performed with 10 mM ^13^C_6_‐Glucose in glucose‐free DMEM supplemented with 20 mM HEPES, and 10% dialysed FBS. For water labelling, 2xDMEM supplemented with 20% dialysed FBS and 40 mM HEPES was diluted 1:1 in ^18^O‐Water. To homogenise the conditions during parallel labellings with the three metabolites (Fig 2), labelling media were prepared as a 1:1 dilution of ^18^O‐Water or Milli‐Q water with 2× Ringer buffer supplemented with 20% dialysed FBS, 40 mM HEPES, 4 mM l‐glutamine and identical concentrations of labelled or unlabelled inositol (100 μg/ml) and d‐glucose (20 mM). In each experiment, incorporation of heavy isotopes was determined by subtracting a baseline signal determined with cells incubated with identical media with unlabelled metabolites. For GPCR‐stimulation experiments, cells were preincubated for 1 h with ^18^O/^2^H‐Inositol or for 5 min with ^13^C_6_‐glucose or ^18^O‐water before addition of the agonist or vehicle.

### PIPn and PA analysis

The analysis of PA and PI was carried out according to published methods (Clark *et al*, 2011). For ^18^O/^2^H‐inositol or ^18^O‐water labelling, the methods for isotope analysis were the same as for Clark *et al*, but with masses adjusted for the inclusion of the isotopes. For ^13^C‐glucose labelling, an alternative fragmentation was applied for C38:4 PI and PA isotopologues to improve the signal/baseline ratio of their labelled versions (see Appendix Table S2). All other parameters were kept the same.

Calibration curves for selected species were constructed from mixtures of PA and PI (see Appendix Fig S1A and B). These were spiked into water at the required concentration and then extracted and derivatised. While this approach has limitations, it allows a correction for different extraction efficiencies during sample preparation and different ionisation/fragmentation efficiencies during analysis by mass spectroscopy. The calibration curves were constructed using 1‐heptadecanoyl‐2‐hexadecanoyl‐sn‐glycero‐3‐phosphoinositol (C17/C16 PI) as the internal standard. Using C17/C16 PA as an ISD for the PA calibrations did not offer any advantage over C17/C16 PI ISD so C17/C16 PI ISD was used for both PA and PI analysis. Unless otherwise mentioned, all data presented for PI and PA were corrected using these calibration curves.

After the indicated treatments, medium was aspirated and cells were killed by addition of 1 ml per well of ice‐cold HCl 1 M. Cells were then scrapped, collected in 2 ml polypropylene Eppendorf tubes, pelleted in a microfuge (15,000 *g*, 10 min at 4°C) and frozen. Frozen pellets were resuspended in 920 μl of primary extraction solution [CHCl3/MeOH/1 M HCl (484/242/23.22)/H2O, 750:170 (v/v)] containing the internal standards C17:0/C16:0‐PIP3 (10 ng) and C17:0/C16:0‐PI (10 ng). Lipids were then extracted using an acidified Folch phase partition and derivatised with TMS‐diazomethane and determined by LC–MS/MS as previously described (Clark *et al*, 2011; Kielkowska *et al*, 2014). Response ratios were calculated for the endogenous species of PA, PI and PIP2 divided by their relevant C17:0/C16:0 internal standard (PI ISD for PI and PA and PIP3 ISD for PIP2). Where indicated, response ratios of selected molecular species of PI and PA were calibrated for differences in their efficiency of detection by LC–MS using a correction factor, calculated as the ratio between the slopes of their calibration curves.

In labelling experiments, we present the calibrated response ratio of defined isotopologues normalised to the total level of PI (i.e. all isotopologues and acyl chain species combined), to correct for differences in cell mass between biological replicates. We also present data for the “fractional enrichment” of individual isotopologues, defined as:

$$\;\left[\text{amount of specified isotopologue}\right]/\left[\text{amount of specified isotopologue}+\;\text{amount of unlabelled species}\right]$$

That is, a measure of the proportion of the pool of an individual acyl chain species that has been labelled. For the ^18^O/^2^H‐inositol or ^13^C_6_‐glucose labelling studies presented, there was good conservation of [PI+3 plus PI+0] isotopologues for each of the species presented, indicating minimal label was incorporated in un‐measured species over the time course studied (e.g. incorporation of label in acyl chains; an example of a species labelling with high fractional enrichment is shown in Appendix Fig S1D). The same was true for the analogous PA species. For the ^18^O‐water labelling studies, the analysis is complicated by the measurement of additional isotopologues for each species (+2/+4 for PI and +2/+4/+6 for PA) and the potential for overlap between species with different degrees of saturation (± 2 amu is equivalent to one saturated/unsaturated bond in an acyl chain; this could be resolved, however, by the different HPLC retention times for the acyl chain species analysed). The PI+2/+4 and PA+4/+6 isotopologues for each species behaved equivalently in our experiments (as would be expected based on the random incorporation of ^18^O‐nuclei into the gamma‐phosphate of ATP), but the +4 isotopologue gave the more robust signal/noise measurements and was our preferred choice for presentation. The relative synthesis of PA+2 vs. PA+4/+6 isotopologues is influenced by the relative activity of DGK vs. PLD in their synthesis, as discussed in the text.

### CDP‐DG analysis

For CDP‐DG analysis, cells were scrapped in ice‐cold PBS and pelleted by centrifugation in 2‐ml microtubes (1 min 5,000 *g*, at 4°C) before snap freezing in liquid nitrogen and storage at −80°C. Cell pellets were resuspended in 300 μl methanol followed by 1 ml tert‐Butyl methyl ether and 250 μl water. The sample was vortexed and then allowed to separate. The upper ether layer was discarded and to the aqueous phase, 400 μl chloroform:methanol (1:2) were added, followed by 100 μl water, 72 μl 2 M HCl and 720 μl chloroform. The sample was vortexed and centrifuged (30 s 5,000 *g* at RT) and the organic phase was transferred into a new microtube containing 700 μl of pre‐derivatization wash solution (fresh acidified aqueous phase from a chloroform:methanol:0.1 M HCl (8:4:3) mixture). The sample was vortexed and centrifuged as previously and the organic phase was transferred in a new microtube, where it was derivatised with TMS‐diazomethane and washed twice with neutral aqueous phase as previously described for PIPn determination (Clark *et al*, 2011; Kielkowska *et al*, 2014). 100 μl methanol:water (9:1) were added to the washed organic phase before evaporating its organic solvents under nitrogen. Finally, the sample was re‐dissolved in 50 μL methanol:water (8:2) and analysed by mass spectroscopy. The sample had to be injected onto the mass spectrometer immediately after preparation due to instability of the analyte. Samples were therefore prepared individually waiting for analysis of the previous sample before preparing the following one. The CDP‐DG mass transitions monitored are listed in the Appendix Table S3.

The methodology was developed using the commercially available CDP standards from Avanti Polar Lipids and verified in biological systems using ^15^N_3_‐Cytidine from Sigma Aldrich Chemical Co (see Appendix Fig S2).

### DG analysis

For DG analysis, cells were washed in ice‐cold PBS, and scrapped in 300 μl of ice‐cold methanol. After transferring the suspension into 2‐ml microtubes, 200 μl of water was added followed by 600 μl of chloroform. 10 μl of deuterated (d6) 18:0–20:4 DG as a 0.88 ng/uL solution in isopropanol was added as internal standard. Samples were vortexed and after phase separation the organic layer was transferred to a vial and a methanol:water mix (9:1; 100 μl) was added to it. The organic layer was then dried carefully under a gentle flow of nitrogen. The aqueous solution was re‐extracted with additional chloroform (300 μL) and combined with the previously extracted material. The sample was again dried gently under nitrogen and then resuspended in 100 μL methanol:water (4:1) for analysis by mass spectrometry (see Appendix Table S1).

### Statistics

Single representative experiments are shown where the interpretation of data was facilitated by internally comparing the behaviour of the different molecular species and isotope‐labelled lipids in the same cells under equivalent experimental conditions. For statistical tests, data from at least 3 biological replicates were pooled and represented as mean ± SEM. When data appeared to be log‐normal, they were log transformed prior to the analysis so that they meet the assumptions for a parametric approach. A two‐way ANOVA followed by Dunnett's multiple comparisons tests was used to analyse the changes in different molecular species. The relative specific activities in WT vs. CDS2‐KO MCF7 cells were analysed with a two‐way ANOVA followed by Sidak's multiple comparisons correction. For time‐course assays, areas under the curve from each molecular species were compared with C38:4 using a one‐way ANOVA, followed by Dunnett's multiple comparisons test.

## Author contributions

**David Barneda:** Conceptualization; Investigation; Methodology; Writing ‐ original draft; Writing ‐ review and editing. **Vishnu Janardan:** Investigation; Methodology. **Izabella Niewczas:** Investigation. **Daniel M Collins:** Investigation. **Sabina Cosulich:** Supervision; Funding acquisition. **Jonathan Clark:** Conceptualization; Investigation; Methodology; Writing ‐ review and editing. **Len R Stephens:** Conceptualization; Supervision; Funding acquisition; Writing ‐ review and editing. **Phillip T Hawkins:** Conceptualization; Supervision; Funding acquisition; Writing ‐ original draft; Writing ‐ review and editing.

## Disclosure and competing interests statement

L.R.S is an EMBO Member; this has no bearing on the editorial consideration of this article for publication. SC is an employee of AstraZeneca plc. The other co‐authors declare that they have no conflict of interest.

## Note added in proof

A parallel study investigating the mechanisms contributing to the unique fatty acid side‐chain composition of phosphoinositides using different approaches has been published in EMBO Reports (Kim *et al*, 2022).

## Supporting information




Appendix

Expanded View Figures PDF

Source Data for Appendix

PDF+

## Data Availability

This study includes no data deposited in external repositories.
