# Supplementary material for: Acyl chain selection couples the consumption and synthesis of phosphoinositides
Source: EMBO J. 2022 Jun 30;41(18):e110038. doi: 10.15252/embj.2021110038 (PMC9475507; doi:10.15252/embj.2021110038)
Supplement: Supplementary file 1 — Appendix [file EMBJ-41-e110038-s002.pdf]

# Appendix

## Table of Contents

Figure S1 Supporting data for measurement of phosphoinositides and DG by mass spectrometry
Figure S2 Validation of measurement of CDP-DG by LC-MS.
Figure S3 Synthesis of 18O 2H Inositol
Figure S4 Validation of CRISPR-mediated mutagenesis and siRNA knock-downs

Table S1 Analysis of DG species by mass spectrometry
Table S2 MRM table for $^{13}C$-labelling measurements
Table S3 Analysis of CDP-DG species by mass spectrometry

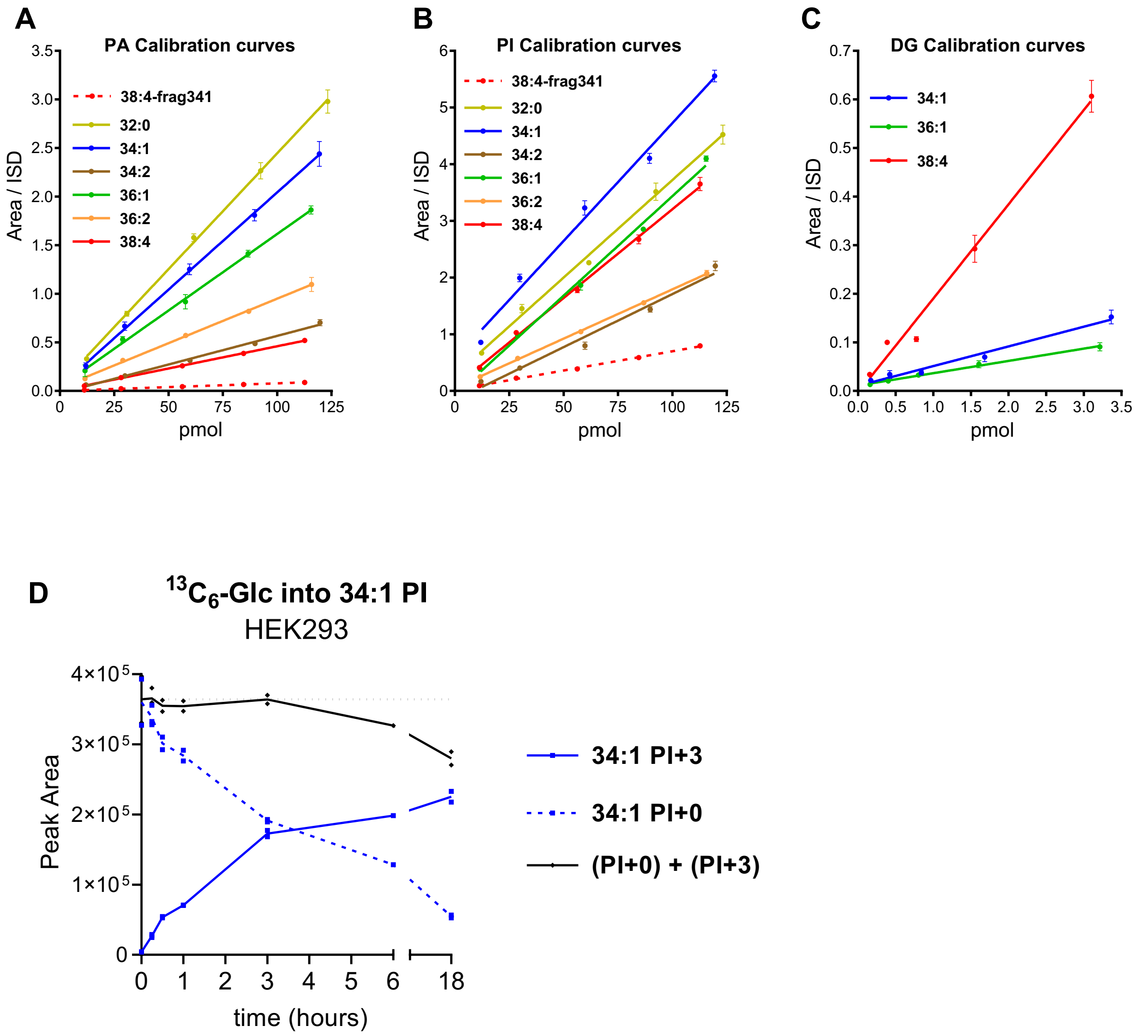


**Figure S1 Supporting data for measurement of phosphoinositides and DG by mass spectrometry**

**Panel A-C:** Examples of the calibration curves for different molecular species of PA, PI and DG. Frag-341 represents an alternative MS fragmentation of the C38:4 species of PA and PI, used in $^{13}C_6$-glucose labelling experiments to improve signal/background ratio of their +3 amu isotopologues. Data are represented as mean ± SD (n=3) of a experiment representative of at least two similar experiments.

**Panel D**: Example of $^{13}C_6$-glucose incorporation into a PI molecular species (34:1 PI) in HEK293 cells. During the first 3 hours of labelling the formation of the 34:1 PI+3 isotopologue matches the decrease in the unlabelled form (34:1 PI+0), indicating that $^{13}C_6$-glucose is incorporated in the glycerol backbone of PI via *de novo* PI synthesis. Data are represented as individual points (n=2 wells/condition) and bisecting lines from a experiment representative of more than 3 experiments.

- 2x10⁵ MCF10a cells
- Plated ± $^{15}N_3$ Cytidine
- Lipid extracted 20 h later
- LCMS to detect CDP-DG

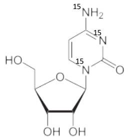

36:2 CDP-DG

36:2 CDP-DG (15N3)

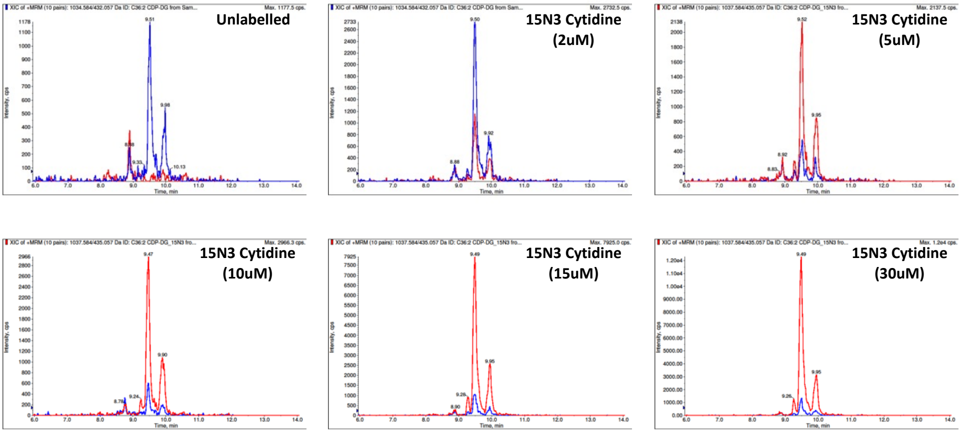


**High resolution fragmentation of C35:1 CDP-DG (standard)**

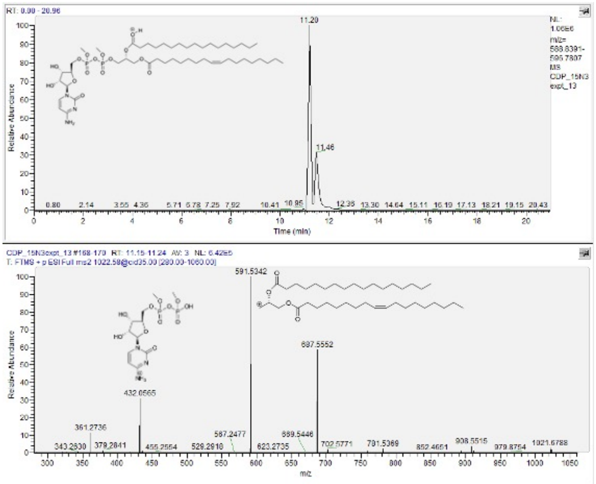


**High resolution fragmentation of C36:2 CDP-DG (MCF10a)**

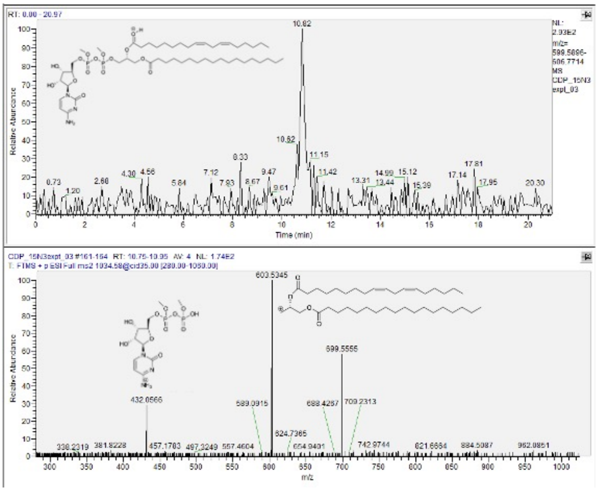


**High resolution fragmentation of C36:2 CDP-DG (MCF10a $^{15}N_3$-cytidine labelled)**

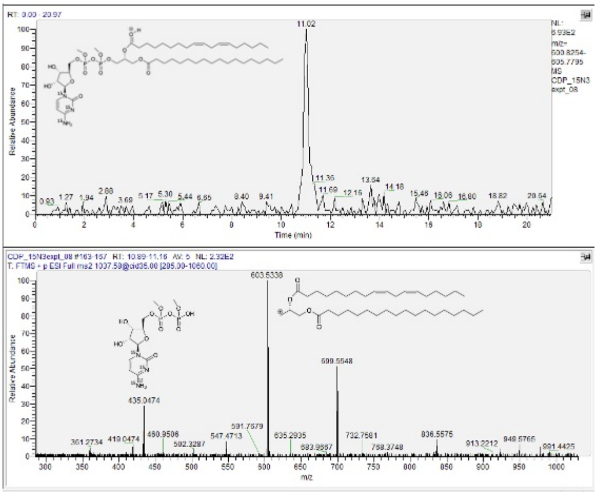


**Figure S2 Validation of measurement of CDP-DG by LC-MS.** MCF10a cells were labelled with $^{15}N_3$-Cytidine showing the expected +3 amu shift in the cytidine diphosphate head-group.

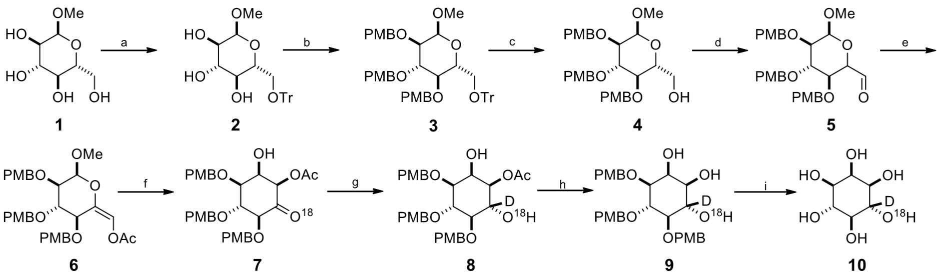


**Figure S3 Synthesis of $^{18}O$ $^{2}H$ Inositol**

Reagents and conditions: (a) Trityl chloride, DMAP, $Et_3N$, DMF, rt, 12 h; (b) NaH, PMB-CI, DMF, 12 h; (c) *p*-TsOH, $NaHCO_3$, MeOH: DCM 2: 1, rt, overnight (d) oxalyl chloride, DMSO, $Et_3N$, $CH_2CI_2$, -78°C to rt; (e) $Ac_2O$, $K_2CO_3$, MeCN, 80°C, overnight; (f) (i) acetone: $H_2O^{18}$ 4:1, $Hg(OAc)_2$, rt, 2h; (ii) NaCI, rt, 20 h; (g) $NaBD(OAc)_3$, MeCN, AcOH, rt, 60 min; (h) $LiOHxH_2O$, MeOH: water 2:1, overnight; (i) Pd/C, $H_2$, atm. pressure, MeOH: water 3 :1, rt, overnight

**2(R)-Acetoxy-3-(R)-hydroxy-4,5,6-(S,R,S)-tri-O-*p*-methoxybenzyl-$^{18}$O-cycIohexahone (7)**:
To the stirred solution of enol acetate **(6)** (0.68 g, 1.14 mmol, 1.0 eq) in dry acetone (3.4 ml) labelled water $H_2{}^{18}O$ (97%, 0.85 ml) was added followed by addition of dry mercuric acetate (0.540 g, 1.69 mmol, 1.48 eq). The resulting, yellow solution was stirred for 2 hr at rt then TLC (petrol: acetone 6: 4) confirmed completion of reaction. NaCl (0.71 g, 12.08 mmol, 10.06 eq) added causing the bright yellow color to disappear and solution became cloudy. The mixture was stirred for 20 h and then solvents removed, diluted with water and extracted with ethyl acetate. The organic layer was washed with brine, dried over $MgSO_4$ and concentrated. Crude material was purified using a chromatography system with RediSep column (12g). Pure white solid **(7)** (0.49 g, 74%) was eluted using petrol: ethyl acetate 3: 7.

**1-O-Acetoxy-6-(R)-deutero-3,4,5-tri-O-p-methoxybenzyl-*myo*-inositoI (8):** Labelled ketone **(7)** (0.45 g, 0.77 mmol, 1.0 eq) was dissolved in dry acetonitrile (41.3 ml). $NaBD(OAc)_3$ (98%, 1.64 g, 7.70 mmol, 10.0 eq) was added followed by addition of AcOH (3.63 ml) and resulting solution was stirred at rt under $N_2$ for 60 min. Progress of reaction was monitored by TLC (hexane: ethyl acetate 3: 7). Reaction quenched with $NaHSO_4$ (0.5 M, 100 ml), ethyl acetate (150 ml) added and phases separated. The organic was washed with $NaHSO_4$ (0.5 M, 2x100 ml), $Na_2HPO_4$ (2M, pH7, 2x200 ml), saturated NaCl (200 ml), dried over $MgSO_4$ and concentrated down. Crude was used in following step without purification.

**$^{18}$O labelled 6-(R)-deutero-3,4,5-tri-O-p-methoxybenzyl-*myo*-inositoI (9):**
Crude diol **(8)** (0.45 g, 0.77 mmol, 1.0 eq) was dissolved in MeOH (18 ml). Water (9 ml) added followed by LiOH (32.4 mg, 0.77 mmol, 1.0 eq) and reaction mixture stirred at rt overnight.
After reaction was complete methanol was removed, water added and extracted with ethyl acetate. Organic phase was combined, dried over $MgSO_4$ and concentrated. Crude reaction mixture was purified on a RediSep column (12g). Solvent system ((A) toluene: ethyl acetate 8: 2 (B) ethyl acetate, B: 0% to 90% over 25 min) to afford desired triol **(9)** (250 mg, 60% over 2 steps) as white solid.

**$^{18}$O labelled *myo*-inositoI (10):**
PMB protected labelled inositol **(9)** (150 mg, 0.28 mmol, 1.0 eq) was dissolved in MeOH: water (3: 1, 16 ml). Palladium on carbon (30 mg) was added and reaction mixture was stirred under hydrogen atmosphere overnight. After reaction was complete the solution passed through a 0.45u PTFE filter to remove Pd/C, solvents removed, water added and product freeze dry to provide labelled inositol as white powder (43.3 mg, 86%).

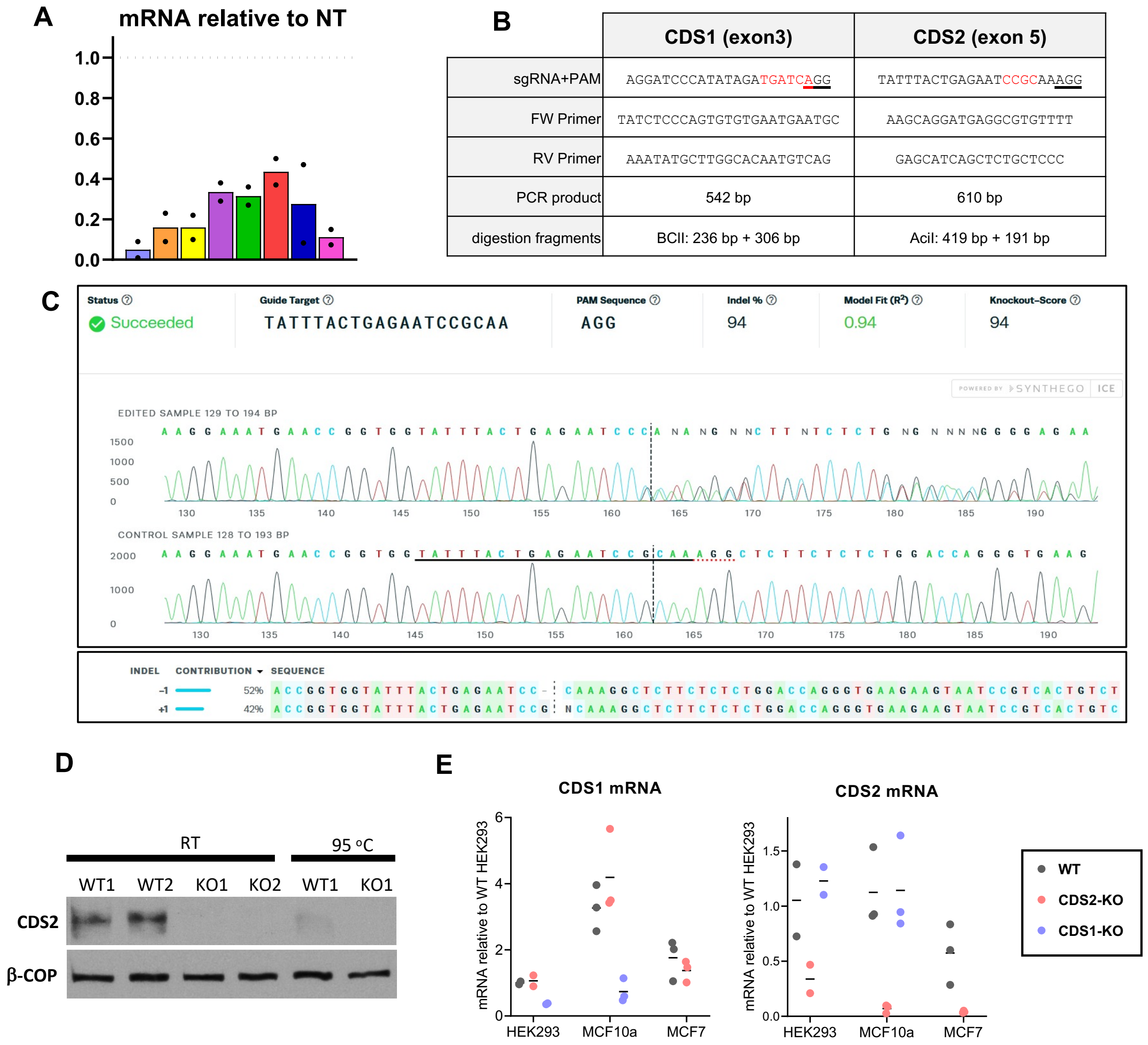


**Figure S4 Validation of CRISPR-mediated mutagenesis and siRNA knock-downs**

**Panel A:** Validation of the indicated siRNA knock-downs in HEK293 cells by qPCR analysis of the target mRNAs 48 hours after transfection with SMARTpool siRNA. Data are represented as individual points with mean bars from 2 independent experiments, where RNA was collected from parallel wells during the labelling experiments (Fig EV3C-E).

**Panel B:** The sgRNA + PAM sequences (underlined nucleotides) used to knock-out CDS1 and CDS2 by CRISPR-Cas9 in human cell lines. The red nucleotides highlight the presence of recognition sequences for restriction enzymes BclI and AciI in the predicted Cas9/sgRNA cleavage sites. To screen the clones generated from single cells transfected with each Cas9/sgRNA, their genomic DNA region flanking the cleavage site was amplified by PCR using the indicated primers and digested with BclI (CDS1 sgRNA) or AciI (CDS2 sgRNA). The undigested PCR products from clones presenting a total loss of the corresponding restriction site were selected for Sanger sequencing to identify the presence of frameshift indel mutations.

**Panel C:** Example of the selection of CRISPR clones by Sanger sequencing followed by ICE analysis. Clones exclusively presenting frameshift indels were assigned as knockouts. Selected clones were further verified by NGS.

**Panel D:** A representative Western blot showing the suppression of CDS2 expression in 2 independent HEK293 clones selected by the presence of frameshift indels in the sgRNA target site (CDS2 exon 5). A specific Western blot protocol was designed to detect endogenous CDS2 (see Methods). CDS2 could not be detected in boiled lysates, and overexpression studies indicated that both CDS1 and CDS2 tended to form aggregates in the lysis buffer (not shown). We could not detect endogenous CDS1 by Western Blot.

**Panel E:** qPCR analysis of the mRNA levels of CDS1 and CDS2, showing a decrease in transcript levels in independent HEK293, MCF10a and MCF7 clones selected by the presence of frameshift indels. The CRISPR/Cas9 targeted gene is indicated in the legend (CDS2, pink; CDS1, blue). We could not isolate MCF7 clones lacking CDS1, as only 6 clones could be expanded from single cells, and all of them presented deletions in the CRISPR site that maintained the reading frame. By contrast, 26 MCF7 clones were isolated from the CDS2 sgRNA preparation, 14 of them identified as knock-outs by ICE analysis.

**Table S1 Analysis of DG species by mass spectrometry**

45uL of the sample in methanol:water (4:1) was injected onto a Waters Acquity UPLC system equipped with a Waters Acquity UPLC BEH300 C4 1.0 x 100mm column at 294K. The sample was then eluted using a 45%-100% acetonitrile in water gradient with 0.1% formic acid added at a flow rate of 100uL/min over 20min (see below).

HPLC gradient table:

| Time | Flow rate uL / min | % water plus 0.1% formic acid | % acetonitrile plus 0.1% formic acid | Curve |
|---|---|---|---|---|
| Initial | 100 | 55 | 45 | 6 |
| 5 | 100 | 55 | 45 | 6 |
| 10 | 100 | 0 | 100 | 6 |
| 15 | 100 | 0 | 100 | 6 |
| 16 | 100 | 55 | 45 | 6 |
| 20 | 100 | 55 | 45 | 6 |

The eluent was then passed into an ABI 4000 QTrap mass spectrometer and the MRM transitions in the table below were monitored. Calibration curves for 18:0-20:4, 16:0-18:1 and 18:0-18:1 DGs against ISD were constructed in the range 0.1ng to 2ng per 100 µL sample. The deuterated internal standard and un-deuterated DGs used for generating calibration curves were made for this study by modification of a procedure previously developed by Prestwich (Chen, J., Profit, A.A., and Prestwich, G.D. (1996). Synthesis of Photoactivatable 1,2-O-Diacyl-sn-glycerol Derivatives of 1-l-Phosphatidyl-d-myo-inositol 4,5-Bisphosphate (PtdInsP2) and 3,4,5-Trisphosphate (PtdInsP3). J Org Chem *61*, 6305–6312. https://doi.org/10.1021/jo960895r ).

| DG | Transition | RT / min |
|---|---|---|
| (d6) 18:0-20:4 | 651.6 > 347.4 | 11.2 |
| 18:0-20:4 | 645.6 > 341.4 | 11.2 |
| 16:0-18:1 | 595.5 > 313.3 | 11.3 |
| 18:0-18:1 | 623.6 > 341.3 | 11.6 |

AB Sciex Instruments 4000 QTrap Mass spectrometer parameters:
Scan Type: MRM, Polarity: Positive, Ion Source: Turbo Spray
Resolution Q1: Unit, Q2: Low, Dwell : 50ms, CUR: 20, IS: 4500, TEM: 300, GS1: 18, GS2: 20, Ihe: ON, CAD: Medium, DP: 100, EP: 10, CE: 35, CXP: 10

**Table S2 MRM table for 13C labelling experiments:**

| analyte | Transition | CE | DP |
|---|---|---|---|
| C32:0 PA | 677.503>551.503 | 35 | 100 |
| C32:0 PA (13C3) | 680.503>554.503 | 35 | 100 |
| C34:1 PA | 703.519>577.519 | 35 | 100 |
| C34:1 PA (13C3) | 706.519>580.519 | 35 | 100 |
| C36:1 PA | 731.55>605.535 | 35 | 100 |
| C36:1 PA (13C3) | 734.55>608.535 | 35 | 100 |
| C36:2 PA | 729.535>603.535 | 35 | 100 |
| C36:2 PA (13C3) | 732.535>606.535 | 35 | 100 |
| C38:4 PA (alternative fragmentation) | 753.535>341.305 | 50 | 120 |
| C38:4 PA (13C3) (alternative fragmentation) | 756.535>344.305 | 50 | 120 |
| C33:0 PI (ISD) | 839.564>565.519 | 35 | 100 |
| C33:0 PIP3 (ISD) | 1163.557>565.519 | 35 | 100 |
| C32:0 PI | 825.548>551.503 | 35 | 100 |
| C32:0 PI (13C3) | 828.548>554.503 | 35 | 100 |
| C34:1 PI | 851.564>577.519 | 35 | 100 |
| C34:1 PI (13C3) | 854.595>580.519 | 35 | 100 |
| C36:1 PI | 879.595>605.55 | 35 | 100 |
| C36:1 PI (13C3) | 882.595>608.55 | 35 | 100 |
| C36:2 PI | 877.58>603.535 | 35 | 100 |
| C36:2 PI (13C3) | 880.58>606.535 | 35 | 100 |
| C38:4 PI (alternative fragmentation) | 901.58>341.31 | 50 | 120 |
| C38:4 PI (13C3) (alternative fragmentation) | 904.58>344.31 | 50 | 120 |
| C34:1 PIP2 | 1067.56>577.519 | 35 | 100 |
| C34:1 PIP2 (13C3) | 1070.56>580.519 | 35 | 100 |
| C38:4 PIP2 | 1117.576>627.535 | 35 | 100 |
| C38:4 PIP2 (13C3) | 1120.576>630.535 | 35 | 100 |

**Table S3 Analysis of CDP-DG species by mass spectrometry**

A Sciex QTRAP4000 mass spectrometer was used for routine analysis. All other parameters as for DG and phosphatidylinositol phosphate analysis. The expected RT for these analytes is between 9 and 10 minutes and usually appear as two peaks. Both peaks were integrated and combined.

High resolution spectra were obtained on an Orbitrap Elite instrument. FTMS + ESI, Full scan MS2, res 240000, NCE 35.0%, IsoW 1.5. Capillary temp 300 C, Source heater temp 350 C, Sheath Gas 35, Aux gas 30. Parent ions were programmed in as required for these fragmentation experiments. HPLC conditions were standard as detailed elsewhere in this paper for the other lipids.

| CDP-DG | Transition programmed into MRM table |
|---|---|
| C35:1 (ISD, Avanti) | 1022.58 > 432.06 |
| C32:2 | 978.52 > 432.06 |
| C32:1 | 980.54 > 432.06 |
| C32:0 | 982.55 > 432.06 |
| C34:2 | 1006.55 > 432.06 |
| C34:1 | 1008.57 > 432.06 |
| C34:0 | 1010.58 > 432.06 |
| C36:4 | 1030.55 > 432.06 |
| C36:3 | 1032.57 > 432.06 |
| C36:2 | 1034.58 > 432.06 |
| C36:1 | 1036.60 > 432.06 |
| C38:4 | 1058.58 > 432.06 |
| C38:3 | 1060.60 > 432.06 |
| C38:2 | 1062.62 > 432.06 |
