# Supplementary material for: Acyl chain selection couples the consumption and synthesis of phosphoinositides
Source: EMBO J. 2022 Jun 30;41(18):e110038. doi: 10.15252/embj.2021110038 (PMC9475507; doi:10.15252/embj.2021110038)
Supplement: Supplementary file 2 — Expanded View Figures PDF [file EMBJ-41-e110038-s003.pdf]

# Expanded View Figures

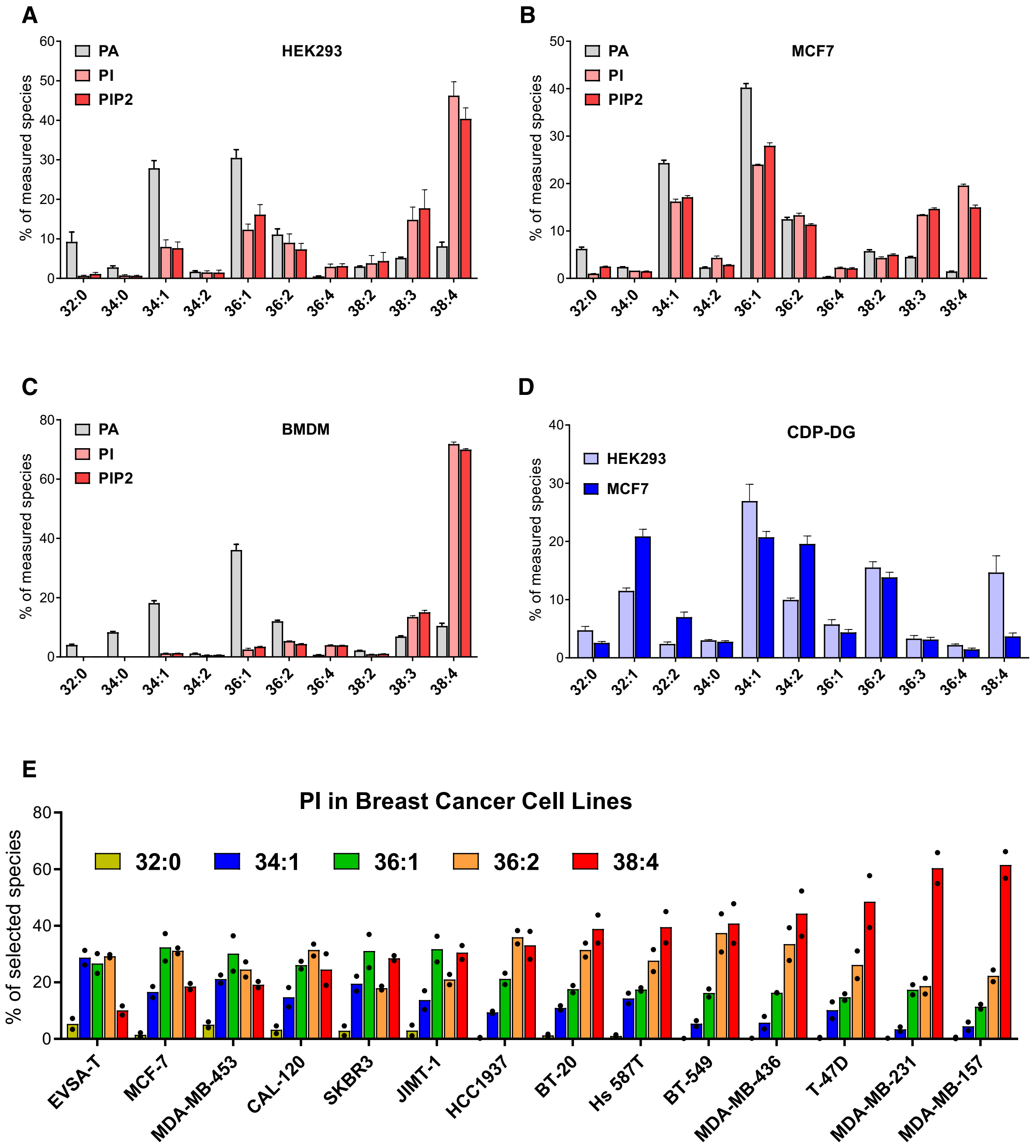


**Figure EV1. Acyl chain composition of phosphoinositides in different cell lines.**

A–C The proportions of all the targeted molecular species of PA, PI and PIP2 measured in BMDMs, HEK293 and MCF7 before applying the correction factors derived from the calibration curves shown in Appendix Fig S1. Data are represented as mean ± SD (*n* = 3) from a single experiment, typical of at least three performed.

D Comparison of all CDP-DG species measured in HEK293 and MCF7. Data are represented as mean ± SEM of 4 biological replicates.

E Distribution of PI species in a panel of breast cancer cell lines cultured in equivalent conditions before lipid extraction (200,000 cells/well seeded in 6-well plates and incubated 24 h in RPMI with 10% FBS and Pen/Strep). Data are represented as individual points with bisecting bars from 2 independent experiments.

© 2022 The Authors

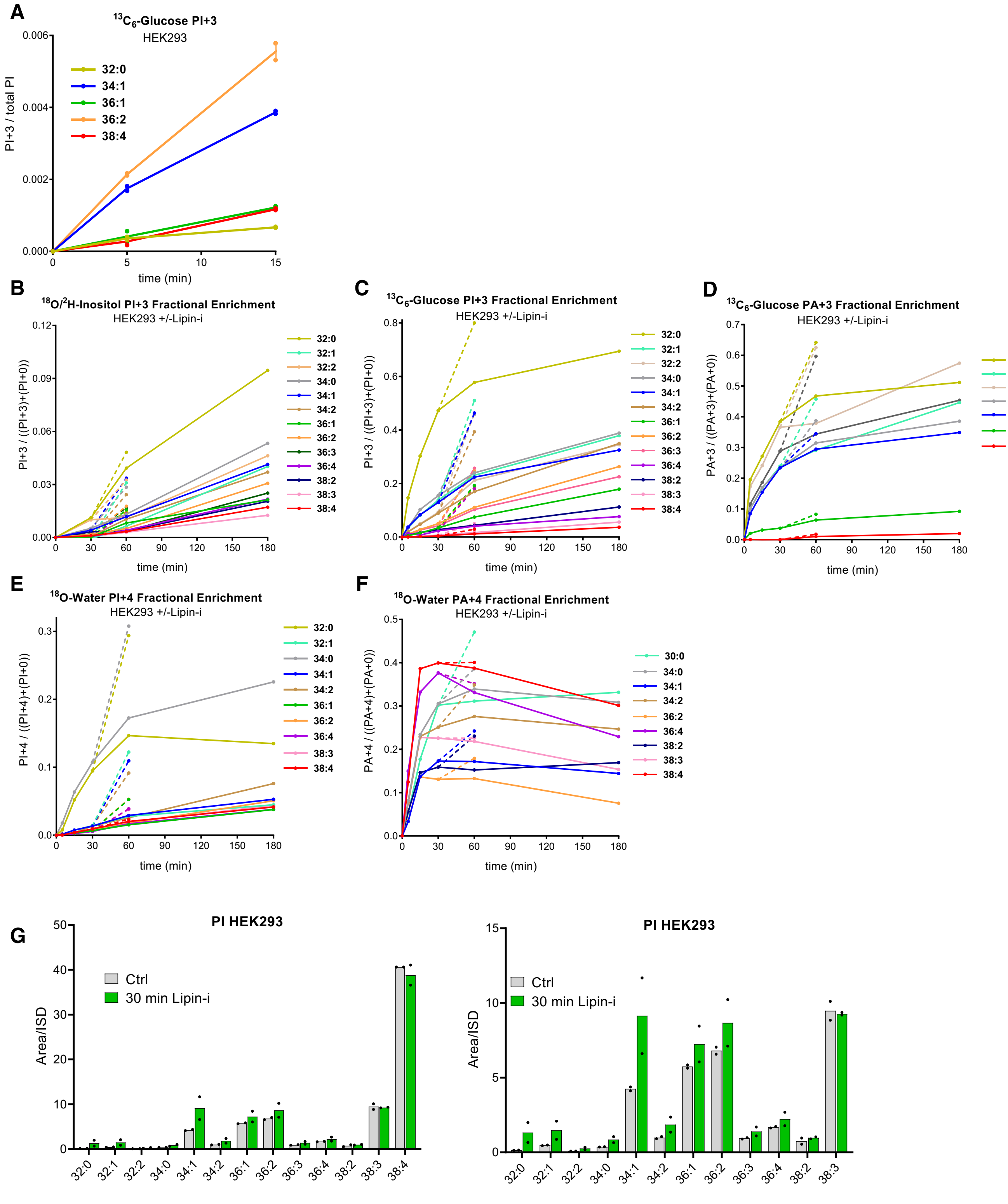


 © 2022 The Authors

**Figure EV2. PI and PA synthesis in HEK293 cells.**

A Re-scaled graph of the early time points only from main Fig 2E, showing the early incorporation of $^{13}C_6$-glucose into the glycerol backbone of PI, highlighting the lagged accumulation of labelled C38:4-PI.

B–F The fractional enrichment of the indicated isotopologues of PA and PI during the incubation of HEK293 cells with $^{18}O/^{2}H$-inositol ("PI+3"), $^{13}C_6$-glucose ("PI+3"; "PA+3") and $^{18}O$-water ("PI+4"; "PA+4"), calculated for each species as the ratio between the labelled isotopologue and the sum of labelled and unlabelled isotopologues. All the targeted molecular species with an adequate signal/background are shown, as this parameter is not affected by differences in the detection efficacy between molecular species. Where indicated, 200 μM propranolol (Lipin-i) was added at 30 min and incubations continued for a further 30 min (broken lines).

G Changes in the levels of unlabelled PI species after 30-min treatment with 200 μM propranolol (Lipin-i). Data represent the response ratio vs. the PI ISD of all targeted species before calibration.

Data information: Data in panels A-F are represented as the means ($n$ = 2 wells/condition) from a single experiment in which all three labelling strategies were performed in parallel, in equivalent media. The results are representative of three similar experiments where each of the labelling strategies were performed in slightly different media, two of which included the addition of propranolol. Data in G are represented as individual points with bisecting bars from 2 independent experiments.

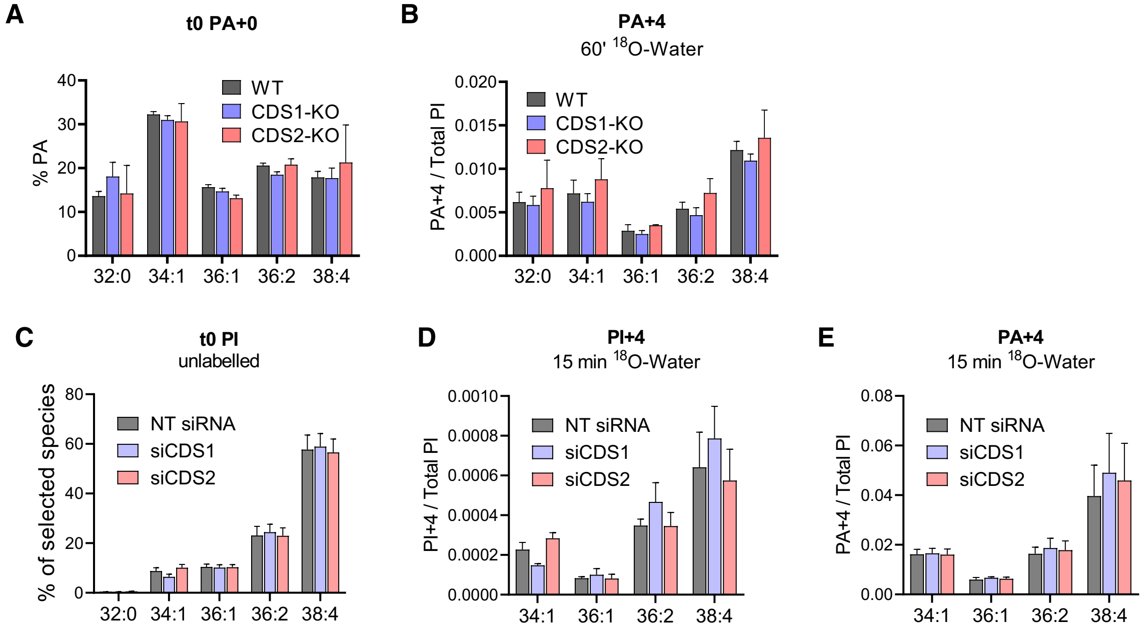


**Figure EV3. The role of CDS isoforms in acyl chain selective synthesis of PI.**

A, B Values for the indicated isotopologues of PA in HEK293 clones in which the genes encoding CDS1 or CDS2 had been deleted (WT; CDS1-KO; CDS2-KO). The proportions of the individual PA acyl chain species at steady-state are shown (t0 PA+0), together with the formation of the PA+4 isotopologue (PA+4) after incubation with $^{18}O$-water for 60 min (normalised to the total level of all PI species to correct for differences in cell mass between clones). Data are represented as mean ± SEM ($n$ = 3 separately derived clones).

C–E Values for the indicated isotopologues of PI and PA in HEK293 cells labelled during 15 min with $^{18}O$-water performed 48 h after transfection with siRNA SMARTpools against CDS1, CDS2 or NT controls. Silencing of the intended target was assessed by mRNA analysis (see Appendix Fig S4A). Data labels are as described in the legend to Fig 3. Data are represented as mean ± SEM of independent experiments ($n$ = 3).

© 2022 The Authors 

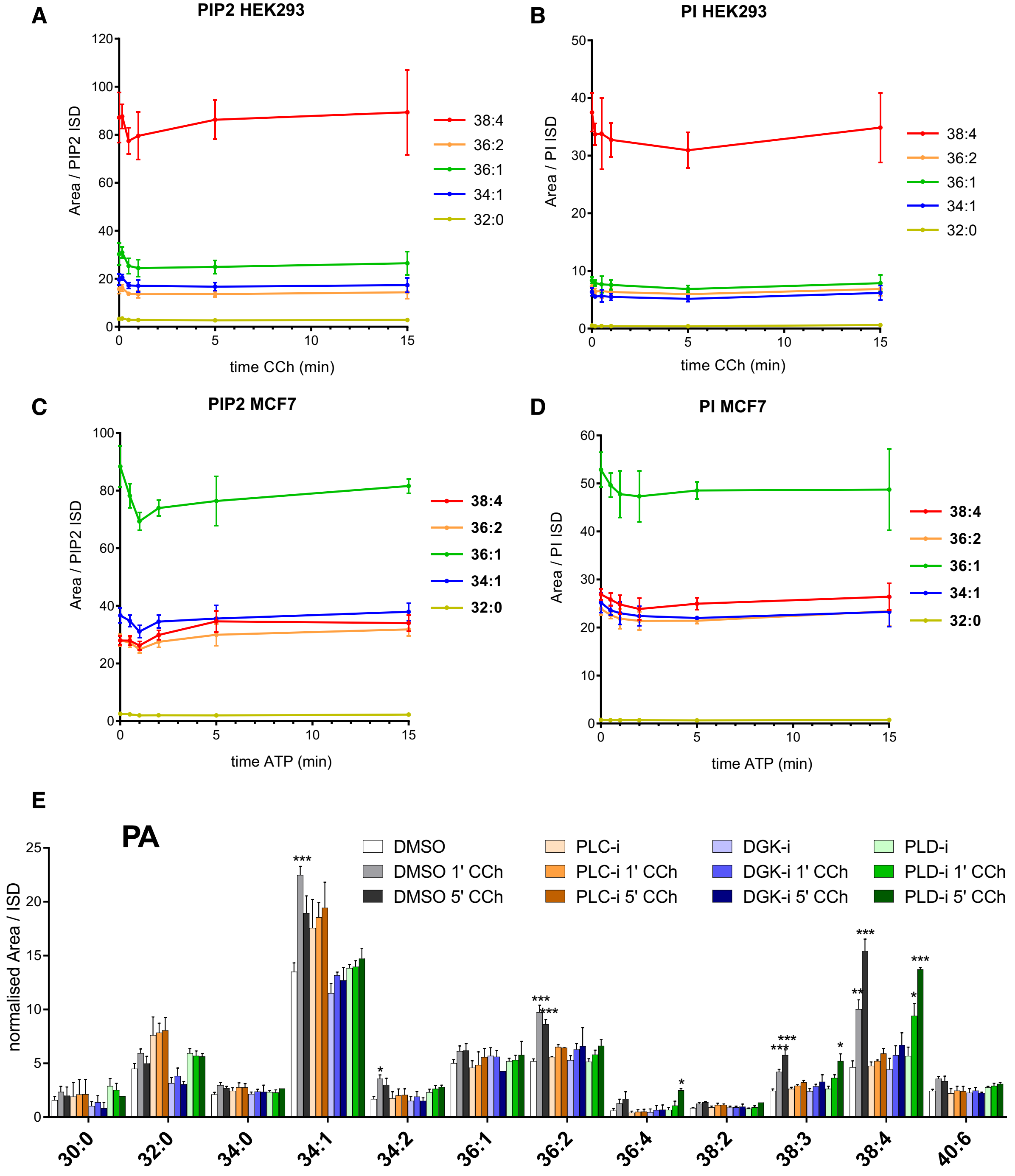


**Figure EV4. GPCR-stimulated changes in phosphoinositide and PA levels.**

A–D Example of a time-course experiment to follow the changes in the selected species of PIP2 and PI after stimulation of HEK293 cells with 100 μM CCh or MCF7 cells with 25 μM ATP. Data are presented as mean ± SD ($n$ = 3 wells/condition) of the uncalibrated response ratios vs. their ISD from a single experiment representative of 2 similar experiments.

E Accumulation of PA species in HEK293 cells stimulated with 100 μM CCh. Prior to stimulation, cells were preincubated for 15 min with vehicle (DMSO) or the indicated inhibitors: 10 μM U73122 (PLC-i), 30 μM R59022 (DGKi), 5 μM ML299 (PLD-i). Data are represented as mean ± SEM, and was generated by pooling seven independent experiments, with each inhibitor tested in at least 3 of them. To compensate for differences between experiments in the response to CCh, the Area/ISD data (uncalibrated) was normalised relative to the total PA levels after 1 min CCh in the DMSO control. Log-transformed data were analysed with a 2-way ANOVA with Geisser–Greenhouse correction, followed by Dunnett's multiple comparisons tests (*$P \leq 0.05$, **$P \leq 0.01$, ***$P \leq 0.001$ vs. the unstimulated DMSO control samples).

 © 2022 The Authors

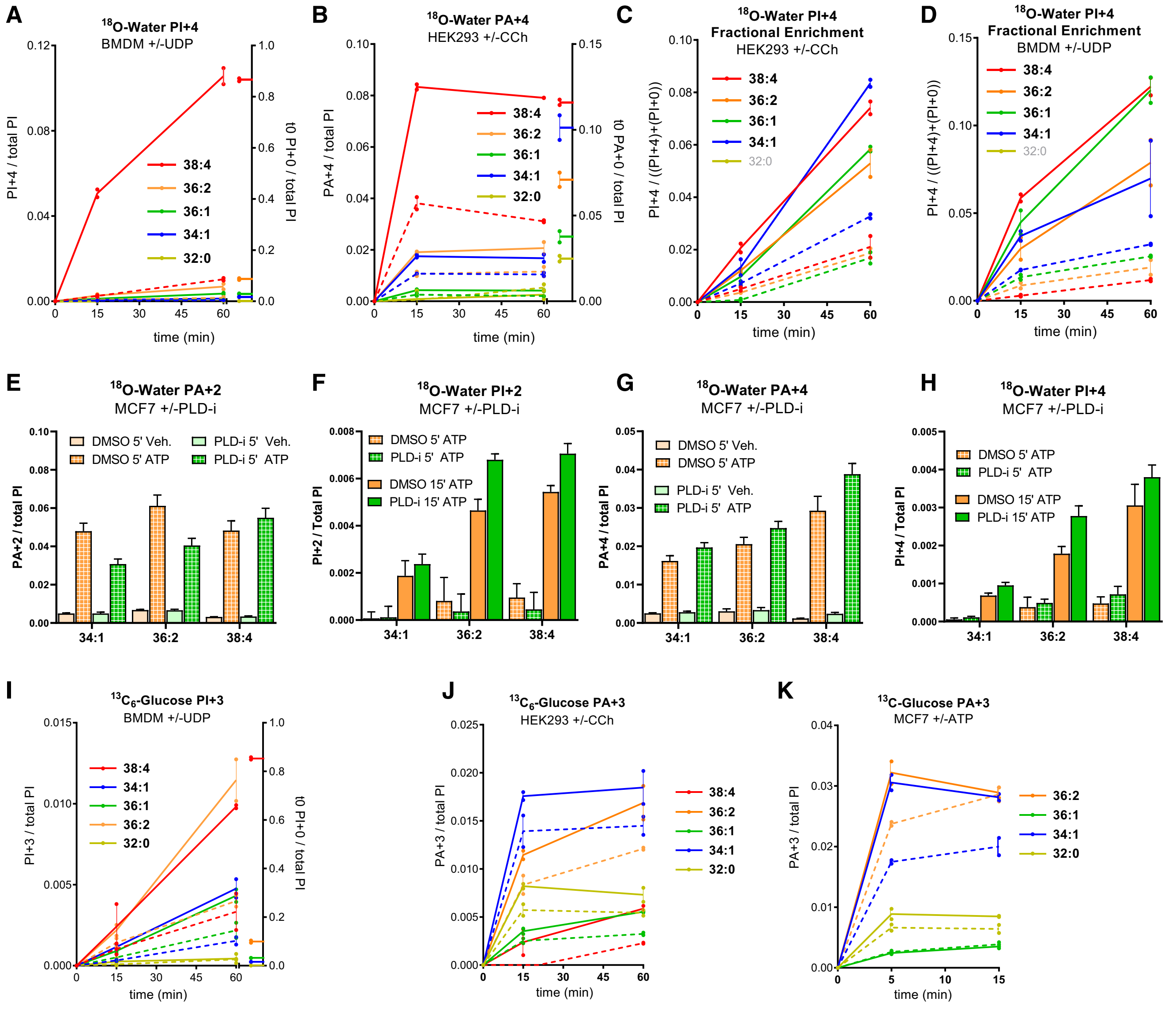


**Figure EV5. GPCRs stimulate acyl chain selective synthesis of PI.**

A–D Incorporation $^{18}$O-water in the indicated PI+4 or PA+4 isotopologues in BMDMs or HEK293 in the presence (solid lines) or absence (broken lines) of 100 μM UDP (BMDMs) or 100 μM carbachol (HEK293). Data labels are as described in the legend to Fig 2. Data are represented as individual points and bisecting lines ($n$ = 2) from a single experiment, representative of three similar experiments.

E–H Incorporation $^{18}$O-water in the indicated isotopologues of PA and PI in stimulated MCF7 cells with or without a PLD inhibitor. Cells were pretreated with 5 μM ML299 (PLDi) or vehicle (DMSO) for 15 min before the incubation with $^{18}$O-water. After a 5 min pre-labelling, cells were treated with 25 μM ATP or vehicle and incubated for a further 5 min or 15 min before quenching with 1 M HCl. Data are represented as mean ± SD (5 min $n$ = 4, 15 min $n$ = 3) from a single experiment, representative of three similar experiments.

I–K The formation of the indicated PI and PA isotopologues during the incubation of BMDMs (I), HEK293 (J) and MCF7 (K) cells with $^{13}$C$_6$-glucose in the presence (solid lines) or absence (broken lines) of 100 μM UDP (BMDMs), 100 μM carbachol (HEK293) or 25 μM ATP (MCF7) added at $t$ = 0. Values of individual isotopologues are normalised to the total level of all PI species, to correct for differences in cell mass (for comparison, in panel (I) the steady-date levels of the unlabelled PI species at $t$ = 0 are shown on the right axis). Data are represented as individual points and bisecting lines ($n$ = 2) from a single experiment, representative of 3 similar experiments.
