# Supplementary material for: Acyl chain selection couples the consumption and synthesis of phosphoinositides
Source: EMBO J. 2022 Jun 30;41(18):e110038. doi: 10.15252/embj.2021110038 (PMC9475507; doi:10.15252/embj.2021110038)
Supplement: Supplementary file 3 — Source Data for Appendix [file EMBJ-41-e110038-s004.zip › embj2021110038-sup-0003-SDataEV/embj2021110038-sup-0003-SDataFigS4D.pdf]

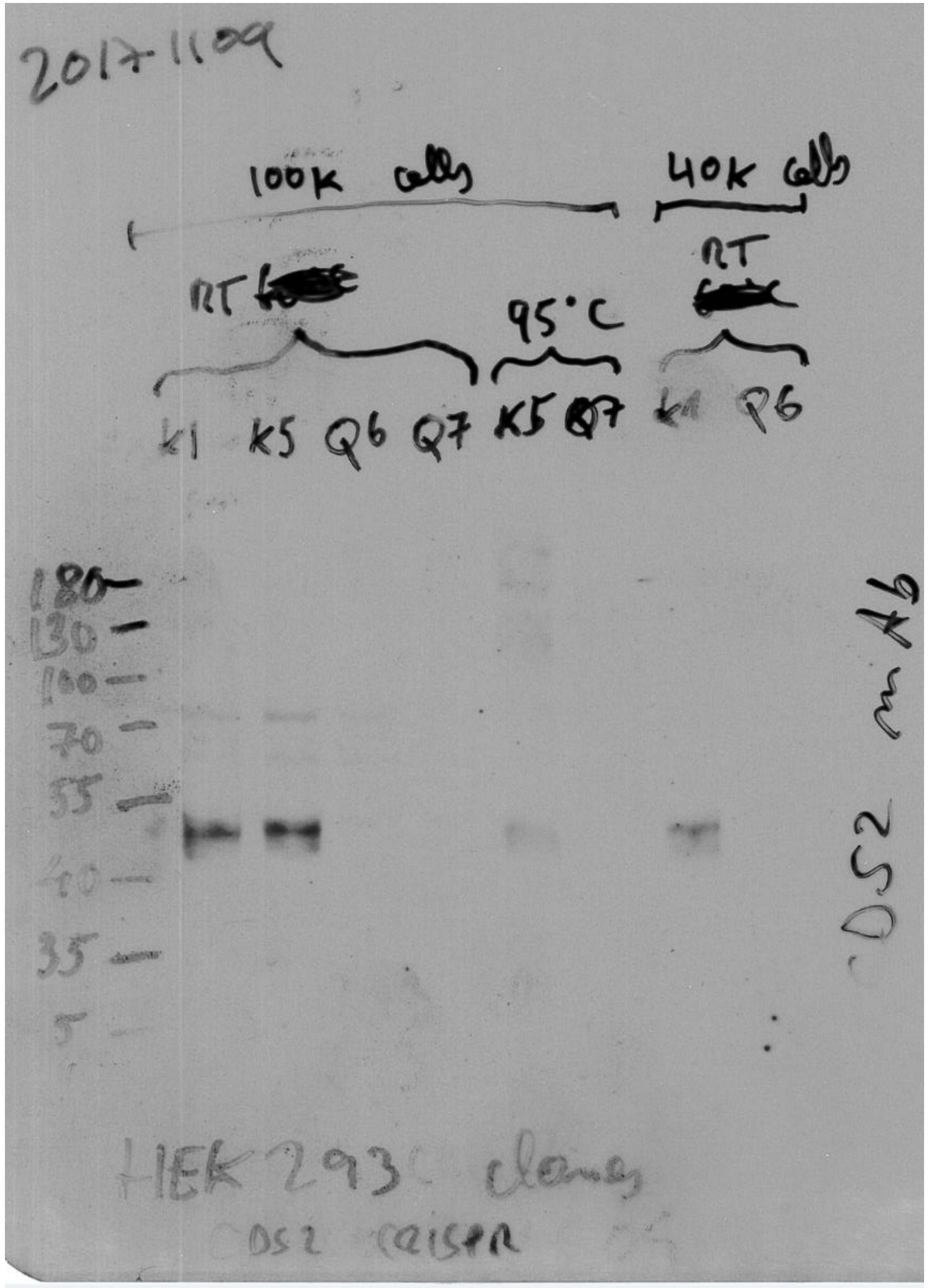

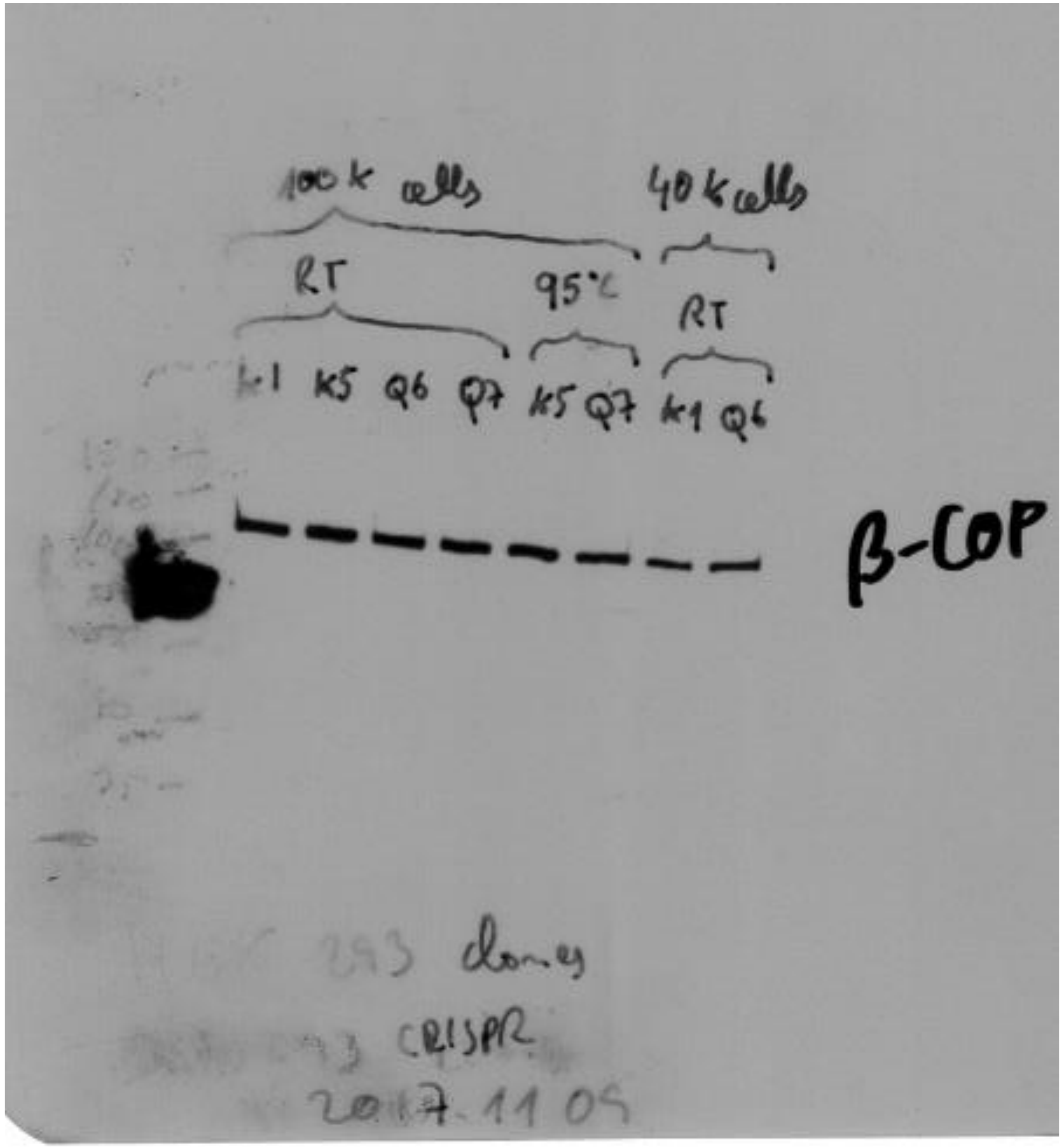

With PageRuler Protein Ladder (Thermo Fisher)

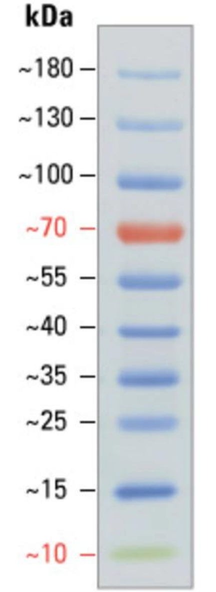

CDS2 (51 kDa) and beta-COP (115 kDa) western blots
HEK293 CRISPR clones: WT (K1 & K5) and CDS2-KO (Q6 & Q7)
Laemmli buffer lysates from 100k or 40k cells (after 5 min incubation at RT or 95 ^{o}C)
