# Supplementary material for: Acyl chain selection couples the consumption and synthesis of phosphoinositides
Source: EMBO J. 2022 Jun 30;41(18):e110038. doi: 10.15252/embj.2021110038 (PMC9475507; doi:10.15252/embj.2021110038)
Supplement: Supplementary file 5 [file EMBJ-41-e110038-s001.pdf]

THE EMBO JOURNAL

Acyl chain selection couples the consumption and synthesis of phosphoinositides

David Barneda, Vishnu Janardan, Izabella Niewczas, Daniel Collins, Sabina Cosulich, Jonathan Clark, Leonard Stephens, and Phillip Hawkins
DOI: 10.15252/embj.2021110038

Corresponding author(s): Phillip Hawkins (phillip.hawkins@babraham.ac.uk) , Leonard Stephens (len.stephens@babraham.ac.uk)

Review Timeline:

Submission Date:	25th Oct 21
Editorial Decision:	29th Nov 21
Revision Received:	14th Apr 22
Editorial Decision:	5th May 22
Revision Received:	11th May 22
Accepted:	12th May 22

Editor: William Teale

Transaction Report:

(Note: With the exception of the correction of typographical or spelling errors that could be a source of ambiguity, letters and reports are not edited. Depending on transfer agreements, referee reports obtained elsewhere may or may not be included in this compilation. Referee reports are anonymous unless the Referee chooses to sign their reports.)

1st Editorial Decision 29th Nov 2021

Dear Dr Hawkins and Dr Stephens,

Thank you again for the submission of your manuscript entitled "Acyl chain selection in the synthesis of phosphoinositides". We have now received the reports from the referees, which I have copied to the bottom of this message.

As you can see from their comments, all referees appreciate the technical accomplishment your work represents and are generally supportive of publication. Therefore, based on this overall interest, I would like to invite you to address the comments of all referees in a revised version of the manuscript. As you will see, certain points are consistently raised across all three reports, meaning some aspects of the study will require a modest degree of further support before we can progress towards publication in The EMBO Journal. After reading the comments carefully, three specific issues presented themselves to me. These are:

Firstly, articles in The EMBO Journal should focus, wherever possible, on the molecular mechanisms which underlie the processes which are studied. Having said this, we very much appreciate interpretations which err on the side of caution. In your case, I wonder whether the balance between these two positions could be pushed a little further towards a mechanistic understanding of selective acyl chain enrichment during the recycling pathway? Perhaps a wider range of hypotheses maybe made accessible by integrating and re-testing datasets as suggested by referee 3 (Major concerns: point 1)?
Secondly, whilst I appreciated the schemes presented in figures 1 and 8, I would have benefitted from an illustration which explains how the incorporation of different isotopes can be used to test fluxes of lipids through different biosynthetic routes. Again a balance must be struck here between keeping the academic heft of your work intact and making at least the rationale behind your experimental design accessible and enlightening to a readers from diverse scientific backgrounds. Maybe a coordinated series of smaller diagrams is a way to achieve this.
Thirdly, I understand that to ask for complementary experiments in interesting genetic backgrounds could set you on a very long path to publication. However, it is also true that, here, this powerful approach is used sparsely. Can you see a way to enrich your conclusions by using additional knock-outs in a way which is achievable within a realistic time-frame?

I should add that it is The EMBO Journal policy to allow only a single major round of revision and that it is therefore important to resolve these concerns at this stage. I believe the concerns of the referees are reasonable and addressable, but we are aware that many laboratories cannot function at full efficiency during the current COVID-19/SARS-CoV-2 pandemic, so please contact me if you have any questions, need further input on the referee comments or if you anticipate any problems in addressing any of their points. Please, follow the instructions below when preparing your manuscript for resubmission.

I would also like to point out that as a matter of policy, competing manuscripts published during this period will not be taken into consideration in our assessment of the novelty presented by your study ("scooping" protection). We have extended this 'scooping protection policy' beyond the usual 3 month revision timeline to cover the period required for a full revision to address the essential experimental issues. Please contact me if you see a paper with related content published elsewhere to discuss the appropriate course of action.

When preparing your letter of response to the referees' comments, please bear in mind that this will form part of the Review Process File, and will therefore be available online to the community. For more details on our Transparent Editorial Process, please visit our website: https://www.embopress.org/page/journal/14602075/authorguide#transparentprocess

Again, please contact me at any time during revision if you need any help or have further questions.

Thank you very much again for the opportunity to consider your work for publication. I look forward to your revision.

Yours sincerely,

William Teale

William Teale, Ph.D.
Editor
The EMBO Journal

When submitting your revised manuscript, please carefully review the instructions below and include the following items:

1) a .docx formatted version of the manuscript text (including legends for main figures, EV figures and tables). Please make sure that the changes are highlighted to be clearly visible.

2) individual production quality figure files as .eps, .tif, .jpg (one file per figure).

3) a .docx formatted letter INCLUDING the reviewers' reports and your detailed point-by-point response to their comments. As part of the EMBO Press transparent editorial process, the point-by-point response is part of the Review Process File (RPF), which will be published alongside your paper.

4) a complete author checklist, which you can download from our author guidelines (https://wol-prod-cdn.literatumonline.com/pb-assets/embo-site/Author Checklist%20-%20EMBO%20J-1561436015657.xlsx). Please insert information in the checklist that is also reflected in the manuscript. The completed author checklist will also be part of the RPF.

5) Please note that all corresponding authors are required to supply an ORCID ID for their name upon submission of a revised manuscript.

6) We require a 'Data Availability' section after the Materials and Methods. Before submitting your revision, primary datasets produced in this study need to be deposited in an appropriate public database, and the accession numbers and database listed under 'Data Availability'. Please remember to provide a reviewer password if the datasets are not yet public (see https://www.embopress.org/page/journal/14602075/authorguide#datadeposition). If no data deposition in external databases is needed for this paper, please then state in this section: This study includes no data deposited in external repositories. Note that the Data Availability Section is restricted to new primary data that are part of this study.

Note - All links should resolve to a page where the data can be accessed.

7) When assembling figures, please refer to our figure preparation guideline in order to ensure proper formatting and readability in print as well as on screen:
http://bit.ly/EMBOPressFigurePreparationGuideline

Please remember: Digital image enhancement is acceptable practice, as long as it accurately represents the original data and conforms to community standards. If a figure has been subjected to significant electronic manipulation, this must be noted in the figure legend or in the 'Materials and Methods' section. The editors reserve the right to request original versions of figures and the original images that were used to assemble the figure.

8) For data quantification: please specify the name of the statistical test used to generate error bars and P values, the number (n) of independent experiments (specify technical or biological replicates) underlying each data point and the test used to calculate p-values in each figure legend. The figure legends should contain a basic description of n, P and the test applied. Graphs must include a description of the bars and the error bars (s.d., s.e.m.).

9) We would also encourage you to include the source data for figure panels that show essential data. Numerical data can be provided as individual .xls or .csv files (including a tab describing the data). For 'blots' or microscopy, uncropped images should be submitted (using a zip archive or a single pdf per main figure if multiple images need to be supplied for one panel). Additional information on source data and instruction on how to label the files are available at .

10) We replaced Supplementary Information with Expanded View (EV) Figures and Tables that are collapsible/expandable online (see examples in https://www.embopress.org/doi/10.15252/embj.201695874). A maximum of 5 EV Figures can be typeset. EV Figures should be cited as 'Figure EV1, Figure EV2" etc. in the text and their respective legends should be included in the main text after the legends of regular figures.

- For the figures that you do NOT wish to display as Expanded View figures, they should be bundled together with their legends in a single PDF file called *Appendix*, which should start with a short Table of Content. Appendix figures should be referred to in the main text as: "Appendix Figure S1, Appendix Figure S2" etc. See detailed instructions regarding expanded view here: .

- Additional Tables/Datasets should be labelled and referred to as Table EV1, Dataset EV1, etc. Legends have to be provided in a separate tab in case of .xls files. Alternatively, the legend can be supplied as a separate text file (README) and zipped together with the Table/Dataset file.

11) Our journal encourages inclusion of *data citations in the reference list* to directly cite datasets that were re-used and obtained from public databases. Data citations in the article text are distinct from normal bibliographical citations and should directly link to the database records from which the data can be accessed. In the main text, data citations are formatted as follows: "Data ref: Smith et al, 2001" or "Data ref: NCBI Sequence Read Archive PRJNA342805, 2017". In the Reference list, data citations must be labeled with "[DATASET]". A data reference must provide the database name, accession number/identifiers and a resolvable link to the landing page from which the data can be accessed at the end of the reference. Further instructions are available at .

Referee #1:

This work aims to understand the molecular mechanisms behind the enrichment of PI and PIP species with C38:4 acyl chains in mammalian cells. The authors developed multiple innovative tracer methods to track the synthesis of PI and PIPs, both through endogenous pathway and also during PLC activation/PI cycle. The authors also examined/compared a few cell lines. Overall, this work addresses a highly important question in lipid research with latest techniques. Most data appear convincing. This work should be accepted for publication after suitable revision.

Concerns and suggestions:

1. As shown in Fig 3, neither CDS1 nor CDS2 is essential for the enrichment of 38:4. This is rather surprising. One possibility is that the genes are not properly knocked out. The knockout needs to be further verified besides western blotting/PCR, especially for CDS1. Absence of CDS1/CDS2 is known to generate supersized lipid droplets (see Qi et al, JLR 2016; Xu et al, JBC 2019), which are easy to detect. The authors should examine the size of LDs in their KO lines.

2. Along the same lines, the authors should also examine knockdowns by siRNA. The CDS enzymes are extremely important to normal cell function. Compensatory pathways may be activated in the KO lines. Therefore, it is worthwhile examining the knockdowns. Again, the knockdown efficacy should also be well controlled.

Referee #2:

The acyl chain profile (that is, the combination of two acyl chains with specific chemistry) of most lipid classes in cells is complex and varies between cell and tissues. It has been long known that the acyl chain profile of phosphoinositides is unique; they display a strong bias towards the C18:0/C20:4 combination. What drives this preferential combination and what advantages it provides to the cell remain mysterious. In this ms, Barneda and coauthors address the first point. This is a very difficult issue. Phospholipids have common precursors, notably phosphatidic acid, at the ER, which suggests that the cells could easily loose the 'memory' of the acyl chain profile of phosphoinositides without a channeling process that would allow, for instance, sorting PA species arising from PI recycling from PA species arising from de novo genesis. Experimentally, distinguishing lipids according to their acyl chains requires complex mass spectrometry analysis, which until know cannot be performed at the subcellular level.

The strength of this ms is two-fold. First, the use of elegant methods for kinetic measurements where the turnover of PI is measured either at the level of the polar head (inositol), of the phosphate or of the glycerol (Fig 2B). Second, the use of three different cell types, with different acyl chain profiles for PI and where the C38 :4 species accounts for 20 to 80% of the total PI pool (Fig 1A). The results are shown in Figures 2 to 4 and offer an unprecedent view of the relative contribution of various pathways of PI synthesis for the formation of the various species. Furthermore, the use of a lipin inhibitor (at time 30 min) nicely complement these kinetics measurements, helping to rank PA and PI species according to the contribution of the de novo pathway vs the DGK pathway. C38:4-PI is clearly formed mostly by the DGK pathway and not by de novo synthesis in HEK cells. MCF7 cells, instead, rely more on the de novo pathway and are not well adapted to recycle the PI pool by DAG regeneration. In the second part of the ms, the authors convincingly address the differences between the three cell types in term of lipid remodeling and recycling. In the third part of the ms, the authors assess the fate of the pool of DAG at the plasma membrane that is formed after PLC-mediated hydrolysis of PIP2. They show that cells successfully retain the acyl chain profile of PIP2 by efficiently channeling the species arising from PLC activation for new PI synthesis. However, the mechanism for this lipid channeling remains unknown.

The discussion summarizes these findings and raises important points for future studies.

Overall, the data are of high quality, compelling and to the best of my knowledge quite unique for a fundamental issue in cell biology. In addition, the authors made a lot of effort at making their experimental strategy understandable despite the fact that the complexity of both lipid chemistry and the pathways that contribute to lipid diversity makes this topic difficult to present. That said, the ms in the present state is mostly descriptive (ranking at the best technical level the contribution of seemingly redundant pathways - neosynthesis, remodeling, recycling), leaving the reader in the mist as regards to the molecular mechanisms that could channel 18:0 38:4 DAG species for faster recycling into phosphoinositides. Is it at the ER level, or rather, which seems the hypothesis of the authors, at membrane contact sites, where PA species arising from steady state or stimulating PIP2 hydrolysis are transferred back from the plasma membrane to the ER? If the authors could provide some first hints as regards to the underlying mechanisms, this paper will be well adapted to the wide audience of the EMBO journal.

Minor points.

In the seventh paragraph of the discussion, the authors speculate on "acyl chain selectivity must occur within the DGK-lipid transfer-CDS steps". It would be helpful if the authors could be more precise here about this chain of events and notably its

localization vs contact sites and lipid exchange reactions.

Referee #3:

Summary

Phosphatidylinositol (PI) and phosphoinositides (PIPn) are known to be enriched in stearoyl-arachidonoyl acyl-chains. This raises two questions. (1) How do these acyl-chains accumulate in PI and PIPn? (2) What is the biological significance of this accumulation? So far, multiple mechanisms leading to their enrichment have been proposed, including a deacylation-reacylation cycle (acyl-chain remodeling) to replace PI acyl-chains, or an accumulation of stearoyl-arachidonoyl species thanks to the backbone selectivity of enzymes implicated in the PI cycle, a metabolic cycle in which PI is subjected to multiple steps of head group modification and synthesized back to PI (Murphy and Folco, 10.1194/jlr.S091371; D'Souza and Epand 10.1016/j.bbamem.2013.10.003). A clear picture of the relative contribution of these two mechanisms of enrichment remains unestablished. In addition, the biological significance of the stearoyl-arachidonoyl enrichment remains suggestive, but it is thought that the backbone of PI enables an efficient recycling of PI through the PI cycle (as has been proposed by the authors in Barneda et al, 10.1042/BST20190205). The authors investigate these issues using state-of-the-art mass spectrometry, isotope labeling, enzyme inhibitors, and genetics. The combination of isotope labeling and lipidomics provide a time-resolved and quantitative view of synthesis and interconversion of lipids.

The authors demonstrate that once synthesized de novo, PI is rapidly converted into the stearoyl-arachidonoyl (in this study illustrated as 38:4) form thanks to acyl-chain remodeling (deacylation-reacylation), at least in bone marrow-derived macrophages (BMDMs). They also demonstrate that the 38:4 form is better recycled back into PI and PIPn in the PI cycle. This efficient acyl-chain-dependent recycling is especially clear during the stimulation of phospholipase C (PLC), and it enables the replenishment of the PIPn pool that has been consumed. While the results presented in this study (the role of remodeling and the selective recycling of 38:4 species in the PI cycle) have already been speculated in the past (see references mentioned above), previous studies were mainly based on enzymology or analysis of steady-state lipid compositions in genetically modified mice. This study provides a quantitative view of metabolic steps in cells with a high temporal resolution. Therefore, the study provides strong evidence at the cellular level about the proposed roles of the 38:4 backbone in PI, which is to promote efficient recycling through the PI cycle, especially upon cell stimulation. On the other hand, the mechanisms leading to the enrichment of a 38:4 backbone in PI remain unclear from the presented evidence. Also, the authors show that a large cell type variation exists in the above observations, and it remains unclear whether common enrichment mechanisms or functions of PI 38:4 exist.

The study confirms the important roles of a lipid with a specific acyl-chain composition, PI 38:4, and this specificity is very remarkable. While the study is relevant for the lipid research field, such a clear distinction of a specific lipid structure might be interesting for readers of other fields too. Indeed, the lack of PI 38:4 has been demonstrate to cause severe brain dysfunctions, and thus this study might have a broader impact.

Strong points of the study

The authors provide detailed time course analyses using various stable isotopes and inhibitors to dissect various metabolic pathways. The ability to measure even minor lipids, such as phosphatidic acid (PA), makes the potential of this approach enormous. The authors provide clear conclusions about the roles of the 38:4 backbone in PI to make the PI cycle efficient during stimulation of PLC. It is fair and very relevant that the authors showed that cell type variability exists in the observations (although this also leads to a weak point of the study, as explained below).

Weak points of the study

Although the study aims in part to understand mechanisms leading to an enrichment of PI 38:4, we are still left without clear conclusion about the relative contribution between acyl-chain remodeling and the PI cycle, and what explains cell type differences in PI 38:4 levels. Even using heavy isotope labelling in relatively short time, the incorporation rates sometimes reflect multiple metabolic steps, which should be better discussed.

Major concerns

1. As stated by the authors, PI is first synthesized de novo and then can have rapid acyl-chain remodeling leading to the enrichment of 38:4 species. It becomes clear in the beginning of Discussion that the authors do not discriminate these two pathways and include both as "de novo synthesis pathway". This view is oversimplified and should be revisited, especially because remodeling has been clearly demonstrated to be important for the synthesis of PI 38:4 (from studies analyzing MBOAT7 or LCLAT1/LYCAT deficiency). In 13C6-Glucose labelling experiments, (PA+3) acyl-chains mostly reflect those acquired de novo, and (PI+3) acyl-chains reflect those inherited from (PA+3) but also those acquired by remodeling. Thus, a difference between (PI+3) and (PA+3) reflect the additive effects of acyl-chain remodeling and the acyl-chain selectivity during (PI+3) synthesis from (PA+3) (from CDS1/2 and PIS). To explain how PI 38:4 is produced, the contribution of de novo synthesis (from G3P to PI) and remodeling pathways (PI to lyso-PI and back to PI) should be separately interpreted and discussed. For example, the initial lag in (PI+3) 38:4 synthesis in Figure 2C and EV2F and its following rapid increase suggest that it is remodeled in part from (PI+3) 34:1 species, which stops increasing at later time points. Lacking this point of view, it is very difficult to understand why the authors can conclude in the discussion that "HEK293 and BMDMs have a recycling that

dominates" and "MCF7 are less active in remodeling". The clear defects in PI 38:4 labelling in Figure 4D as compared to Figure 4C demonstrate that BMDM are very active in remodeling, thus it is hard to understand how the authors conclude that "recycling dominates". Since the authors have a large amount of data analyzing labelling of PI and its precursors at various time points and cell lines, it is very likely that a more detailed dissection of different metabolic pathways are possible, and for this reason I encourage the authors to provide a better view about how de novo synthesis, acyl-chain remodeling, and PI recycling, could contribute to the maintenance of PI 38:4 levels, and how these pathways differ between HEK293, MCF7, and BMDM. A better integration of their repeated experiments with statistics (the authors mostly report representative duplicate experiments) would also help for this aspect. Otherwise, if this is not something doable from the current datasets, discuss why.

2. While the combination of mass spectrometry and isotope labelling used by the authors has an enormous potential, the complexity of lipid metabolism makes it very difficult to interpret the labelling experiments. As stated above, glucose labelling reflects acyl-chains acquired during de novo synthesis of PI, but also the following remodeling steps, and discriminating both is difficult except in Figure 4. As stated by the authors, inositol labelling reflects acyl-chains acquired during de novo synthesis, remodeling, recycling, and possibly the exchange mediated by PI synthase (PIS), and discriminating these possibilities is difficult. Water labelling affects phosphate groups, which are incorporated either in glycerol 3-phosphate (the first substrate of de novo PI synthesis) or in phosphatidic acid (PA) generated by diacylglycerol kinase (DGK) in the PI cycle. Thus, heavy isotope enrichment in this experiment reflects either acyl-chains acquired during de novo synthesis followed by remodeling, or recycling in the PI cycle, and the relative contribution of them might depend on the cell type. Therefore, none of the labelling strategies is perfect for discriminating metabolic pathways, and thus extreme care is required for the interpretation. For this reason, the authors are recommended to provide an illustration about which step the heavy isotopes are supposed to be incorporated, for example in Figure 1A. In addition, a clear discrimination between de novo synthesis and PI recycling is critical for the interpretation of water labelling experiments, thus the authors should repeat the experiments in the presence of a DGK inhibitor. DGK inhibition will remove the contribution of the PI cycle in water labelling experiments, and thus the differences in acyl-chain profiles of labelled PI with or without the inhibitor will reflect what are the species that are recycled in the PI cycle. This will provide a stronger evidence that 38:4 species are more efficiently recycled than other species (if it is indeed the case).

3. The term "specific activity" is used throughout the article, but not clearly defined (only in Figure 2 legend) and its significance is not clear. This term is in general used for radioisotopes, thus it would be beneficial for readers to mention in the main text how authors define specific activity and how this can be interpreted. According to what we read, "specific activity" seems to be the level of an isotope-labelled species normalized with the sum of this species and the corresponding non-labelled species, but what could this reflect? The authors consider it to illustrate how fast a species will turnover, which indeed could be true. However, it could also mean that a species with low "specific activity" have additional synthetic pathways to explain its steady-state levels, and thus concluding something from specific activity sounds very challenging. Then, "relative specific activity" found in Figures 7H and 7J has even more calculations behind, and its interpretation is even more complicated, although no detailed explanations are made. The experiments of Figure 7 are very important to interpret the enzymes implicated in the efficient recycling of PI 38:4, thus a better explanation and interpretation is required. I would thus suggest avoiding discussing too much from these specific activity values, unless the authors can demonstrate the clear benefit of analyzing these parameters.

4. One very difficult aspect of this study is that the acyl-chains of recycled PI are necessarily affected by the pre-existing pool of PI. Indeed, the authors convincingly demonstrate that PLC-derived DAG is efficiently converted back into precursors for PI synthesis (Figures 6J and K and EV6B), thus an enrichment of 38:4 species during recycling would not necessarily demonstrate acyl-chain specific recycling, but rather could be a consequence of 38:4 enrichment in the pre-existing PI pool, especially in HEK293 and BMDMs. Therefore, 38:4 being rich in labelling experiments analyzing PI recycling is not a demonstration of the specificity of this pathway, in the case of HEK293 and BMDMs. For this reason, the authors are completely right to mention that the diversity of PI in MCF7 offers an opportunity to study the selectivity for recycling (page 10). Indeed, the authors take advantage of this diversity in Figure 7, in which they demonstrate that PI recycling upon ATP stimulation can enrich 38:4 species despite the relative paucity of PI 38:4. It will be critical to investigate whether the PI cycle enriches PI 38:4 also in unstimulated conditions, for which water labelling experiments in MCF7 with a better discrimination of de novo synthesis and PI cycle (see above comments) could give answers.

Minor concerns

5. The acyl-chains of PI species that increase upon lipin inhibition are coming from the increased PA pool, thus would be strongly affected by lipin substrate preference. It is therefore difficult to conclude whether they would be identical to PI species synthesized de novo when lipin is not inhibited.

6. In Figure 6, the authors use a phospholipase D inhibitor to investigate the contribution of PLC indirectly. A more direct demonstration of the roles of PLC would be possible if using PLC inhibitors. Is there a reason why this was not done?

7. As mentioned by the authors, culture conditions strongly affect polyunsaturated fatty acid levels, thus have a strong impact on PI 38:4. It is therefore very important to explain how the cells were prepared (how many days of culture before labelling experiments, cell densities) for the experiments. Otherwise, it is difficult to know whether the differences between MCF7 cells and the others are due to cell type differences or due to different culture conditions.

8. Figure 1A, what is released during phospholipase reactions is not acyl-CoA, but a free fatty acid.

9. Mass spectrometry is key for the study, thus it is surprising to see that almost no explanations are made about the methods. What are the mobile phases used, which type of mass spectrometer is used, and whether targeted or untargeted approaches are used, are all missing.

10. In Figure EV3, a reduction in CDS1 mRNA does not demonstrate that it is knocked out. The authors should demonstrate that genome DNA has indeed been fully mutated (for example by TIDE or next generation sequencing).

11. The calculations made by the authors "We estimate that the maximum contribution that the pathway reported by..." at the end of the second part is important for the conclusions of the paper, but there is no explanation about how this was calculated.

12. PI 38:4 is clearly important in vivo, considering the strong phenotypes of Mboat7 knockout mice and the genetic diseases caused by MBOAT7 deficiency. This should be at least mentioned in the text.

Suggestions
13. A more detailed understanding about enzymes implicated in the processes of remodeling and PI cycle would drastically improve the manuscript. The authors use knockout cells of CDS1, CDS2, or MBOAT7 only occasionally. More genetic approaches would be very interesting, especially if done in the cell line. For example, labelling experiments in MBOAT7 knockout HEK293 cells would strongly complement the study. Otherwise, DGK epsilon is also a very important enzyme in the 38:4-selective recycling process, thus its knockout would be also very interesting. It is indeed very surprising to see so little discussion about this enzyme in this manuscript.

Point by point response to first review

We would like to thank the referees for their detailed and insightful comments and we're very grateful for the time they have spent in its careful review. This has led to a much more accessible manuscript and the inclusion of some important new data.

Response to the Editorial overview

I wonder whether the balance between these two positions could be pushed a little further towards a mechanistic understanding of selective acyl chain enrichment during the recycling pathway? Perhaps a wider range of hypotheses maybe made accessible by integrating and re-testing datasets as suggested by referee 3 (Major concerns: point 1)?
A re-examination of our data suggested conversion of DG to PA is the most likely point of acyl chain discrimination in the PI-recycling pathway. We therefore focused on this step by measuring DG levels (Fig 1C; Fig 7C), employing DGK-inhibitors (Fig 3A,C) and knocking down the expression of six of the most highly expressed isoforms of DGK in our cells (Fig 3B,D). This new data clearly identifies DGK as a major point of acyl chain selectivity in the synthesis of PI.

Secondly, whilst I appreciated the schemes presented in figures 1 and 8, I would have benefitted from an illustration which explains how the incorporation of different isotopes can be used to test fluxes of lipids through different biosynthetic routes.
We have introduced new illustrations describing the incorporation of isotopologues into the relevant biosynthetic pathways in Fig 2 (panels A,D,I).

Thirdly, I understand that to ask for complementary experiments in interesting genetic backgrounds could set you on a very long path to publication. However, it is also true that, here, this powerful approach is used sparsely. Can you see a way to enrich your conclusions by using additional knock-outs in a way which is achievable within a realistic time-frame?
Given the new focus on DGK we attempted to use genetic manipulation to support our studies using DGK-inhibitors. There are ten different isoforms of DGK expressed in mammalian cells, so we first tried an RNAi screen against the six isoforms for which we could detect expression in the HEK293 and MCF7 cells used in this study. Despite substantial knock-downs in expression (Appendix Fig S4A) we found only marginal effects on the rate of PA and PI synthesis compared to the use of broad range DGK-inhibitors (Fig 3A-D). This is an interesting result in its own right (specificity is very unlikely to be the sole property of an individual isoform) and suggested any further clarity would require knock-out of multiple isoform combinations, which would not be achievable in the time frame available.

We also respectfully note that we present data investigating CRISPR-mediated knock-out of CDS1 and CDS2 in multiple clones of three different cell lines (HEK293, MCF7 and MCF10) and also the knock-out of LPIAT1 in the myeloid lineage of the mouse.

Response to Referees

Referee #1:

1. As shown in Fig 3, neither CDS1 nor CDS2 is essential for the enrichment of 38:4. This is rather surprising. One possibility is that the genes are not properly knocked out. The knockout needs to be further verified besides western blotting/PCR, especially for CDS1. Absence of CDS1/CDS2 is known to generate supersized lipid droplets (see Qi et al, JLR 2016; Xu et al, JBC 2019), which are easy to detect. The authors should examine the size of LDs in their KO lines.
We apologise that our strategy for verifying knockouts was only mentioned in the methods and that all supporting data was not shown. We confirmed that the genes were 'properly knocked out' using multiple approaches (Western blots, TIDE/ICE analysis and NGS) and now include further results from these analyses in Appendix Fig S4B-E.

For the referee's interest, we have also knocked-out the gene encoding CDS2 in the myeloid lineage of the mouse. We found no difference in the acyl chain distribution of PI species in bone marrow derived macrophages isolated from these mice compared to the WT (see Figure 1 below). As the referee predicts, we also saw a substantial increase in triglyceride in CDS2-KO BMDMs. The characterisation of this mouse will be published separately, since it is aimed at answering a different scientific question.

Figure for referees not shown

2. Along the same lines, the authors should also examine knockdowns by siRNA. The CDS enzymes are extremely important to normal cell function. Compensatory pathways may be activated in the KO lines. Therefore, it is worthwhile examining the knockdowns. Again, the knockdown efficacy should also be well controlled.
The referee makes a good point here about possible compensation in the KO lines (we also note that knock-out of CDS1 in the MCF7 cells was not viable). We have now used RNAi to knock-down expression of CDS1 and 2 in the HEK293 cells and we obtained very similar results to those we observed with the KOs (Fig EV3C-E; text P8). Overall, we are convinced that CDS1 and CDS2 have individual substrate preferences (see the combined data from all of our KO clones in both HEK293 and MCF10a cells; now moved from expanded view into main Fig 3I and J) but that acyl chain remodelling is the major route for basal C38:4 enrichment (as confirmed by comparing WT and LPIAT1-KO BMDMs in Fig 4).

Referee #2:

…….leaving the reader in the mist as regards to the molecular mechanisms that could channel 18:0 38:4 DAG species for faster recycling into phosphoinositides. Is it at the ER level, or rather, which seems the hypothesis of the authors, at membrane contact sites, where PA species arising from steady state or stimulating PIP2 hydrolysis are transferred back from the plasma membrane to the ER? If the authors could provide some first hints as regards to the underlying mechanisms, this paper will be well adapted to the wide audience of the EMBO journal.
Minor points.

In the seventh paragraph of the discussion, the authors speculate on "acyl chain selectivity must occur within the DGK-lipid transfer-CDS steps". It would be helpful if the authors could be more precise here about this chain of events and notably its localization vs contact sites and lipid exchange reactions.
The referee goes straight to the heart of the issue here. We now present new data for DG levels in both basal (Fig 1C) and GPCR-stimulated cells (Fig 7C), which when compared to the analogous rates for ^{18}O-H_2O-incorporation into PA and PI synthesis clearly point to DGK as a major point of acyl chain selectivity in the PI-recycling pathway, a conclusion confirmed by the use of DGK-inhibitors. The text has now been substantially re-written to include a more extensive and precise interpretation of this and previous data to conclude that both DGK and CDS2 support acyl chain selection in the PI recycling pathway (see P11, P14-15).

Unfortunately, we do not have any data that directly addresses the localisation of the re-cycling pathway and its relationship to contact sites and lipid exchange reactions, but given it must use DG/PA species that have previously been shown to be transported across contact sites it must also utilise lipid exchange reactions (as discussed on P14, second paragraph). The involvement of acyl chain-selective transport proteins is something that we are currently trying to pursue.

Referee #3:

Major concerns
1. As stated by the authors, PI is first synthesized de novo and then can have rapid acyl-chain remodeling leading to the enrichment of 38:4 species. It becomes clear in the beginning of Discussion that the authors do not discriminate these two pathways and include both as "de novo synthesis pathway". This view is oversimplified and should be revisited, especially because remodeling has been clearly demonstrated to be important for the synthesis of PI 38:4 (from studies analyzing MBOAT7 or LCLAT1/LYCAT deficiency).
It was certainly not our intention to over simplify these two processes, but to link them in sequence to explain how the basal composition of PI is created. The referee makes a good point however, and we now use the term '*de novo synthesis-remodelling pathway*' (P13, Fig 8) to describe how PI molecules made recently from G3P (ie made *de novo*) are subsequently remodelled towards C38:4. In principle, remodelling could occur on both PIs made *de novo* and on those in the *recycling* pathway, but the rapid, C38:4-selective synthesis of PA+4/PI+4 and the lack of effect

of the knock-out of LPIAT1 in the water-labelling experiments suggests it doesn't have a major impact on the recycling pathway (this argument is now made more explicitly, P13, last paragraph).

We obviously agree that remodelling is important for synthesis of C38:4-PI, since we demonstrate it clearly in this study (eg Fig 4); we also refer to previous studies of LPIAT1/MBOAT and LYCAT deficiency.

In 13C6-Glucose labelling experiments, (PA+3) acyl-chains mostly reflect those acquired de novo, and (PI+3) acyl-chains reflect those inherited from (PA+3) but also those acquired by remodeling. Thus, a difference between (PI+3) and (PA+3) reflect the additive effects of acyl-chain remodeling and the acyl-chain selectivity during (PI+3) synthesis from (PA+3) (from CDS1/2 and PIS). To explain how PI 38:4 is produced, the contribution of de novo synthesis (from G3P to PI) and remodeling pathways (PI to lyso-PI and back to PI) should be separately interpreted and discussed. For example, the initial lag in (PI+3) 38:4 synthesis in Figure 2C and EV2F and its following rapid increase suggest that it is remodeled in part from (PI+3) 34:1 species, which stops increasing at later time points.

We agree in principle but, unfortunately, we think our PA+3 vs PI+3 data do not have the fidelity to delineate the precise sequence of remodelling events ie which species are formed from which (other than that C32:0-PI is likely made first, P6). As the referee points out, the key observation is that in glucose-labelling of HEK293 cells there is a very distinct lag in C38:4-PI+3 synthesis (which is the very reason we included an expanded view of early times in Fig EV2B). This, together with the clear observation that very little C38:4-PA+3 is generated *de novo,* is very strong evidence that C38:4-PI+3 is generated by remodelling (a concept proven in BMDMs by knocking-out LPIAT1). This is really the main point that we were trying to make, not the details of the remodelling pathway itself (which we believe would require an entirely separate study employing acyl chain tracers and systematic deletion of acyl-CoA transferases – an argument we make on P13, start of third paragraph).

Lacking this point of view, it is very difficult to understand why the authors can conclude in the discussion that "HEK293 and BMDMs have a recycling that dominates" and "MCF7 are less active in remodeling". The clear defects in PI 38:4 labelling in Figure 4D as compared to Figure 4C demonstrate that BMDM are very active in remodeling, thus it is hard to understand how the authors conclude that "recycling dominates".

The main evidence that MCF7s are less active in remodelling [towards C38:4-PI] is the glucose labelling data that indicates initial PIs made *de novo* do not undergo the same level of subsequent re-engineering [to enrich for C38:4-PI] as those seen in the HEK293s and BMDMs. However, both HEK293s and BMDMs also have very active PI-recycling pathways, as evidenced by their relatively high rates of incorporation of ^{18}O-H_2O into both PA and PI from pre-existing DG. The term 'dominates' was an attempt to refer to the relatively small effects of lipin-inhibition on both water-labelling and inositol-labelling in the HEK293 cells and the lack of effect of LPIAT1 knock-out on water-labelling in the BMDMs. We agree however that this is perhaps unhelpful and have removed it in favour of a simpler description of the results (P13).

Since the authors have a large amount of data analyzing labelling of PI and its precursors at various time points and cell lines, it is very likely that a more detailed dissection of different metabolic pathways are possible, and for this reason I encourage the authors to provide a better view about how de novo synthesis, acyl-

chain remodeling, and PI recycling, could contribute to the maintenance of PI 38:4 levels, and how these pathways differ between HEK293, MCF7, and BMDM.

We really appreciate the efforts this referee has made in trying to follow our arguments and apologise for the lack of clarity. We have rewritten the relevant parts of the Discussion (P13, last paragraph) and hope that our explanations for how *de novo* synthesis, acyl chain remodelling and PI-recycling differ between HEK, MCF7 and BMDMs are now clearer.

A better integration of their repeated experiments with statistics (the authors mostly report representative duplicate experiments) would also help for this aspect. Otherwise, if this is not something doable from the current datasets, discuss why.

We have only presented representative experiments where we believe comparisons between different labelling strategies in the same large experiment, with the same cells, under precisely the same conditions, is most important (eg Fig 2). In the lead up to these 'set piece' experiments, several experiments were performed where individual labelling strategies were performed in isolation, with the same qualitative pattern of results, but where small differences in conditions make combining data unhelpful. We have tried to be scrupulously clear about the number and type of experiments performed and to use statistics only where appropriate eg where it has been possible to combine true biological replicates.

2. While the combination of mass spectrometry and isotope labelling used by the authors has an enormous potential, the complexity of lipid metabolism makes it very difficult to interpret the labelling experiments. As stated above, glucose labelling reflects acyl-chains acquired during de novo synthesis of PI, but also the following remodeling steps, and discriminating both is difficult except in Figure 4. As stated by the authors, inositol labelling reflects acyl-chains acquired during de novo synthesis, remodeling, recycling, and possibly the exchange mediated by PI synthase (PIS), and discriminating these possibilities is difficult. Water labelling affects phosphate groups, which are incorporated either in glycerol 3-phosphate (the first substrate of de novo PI synthesis) or in phosphatidic acid (PA) generated by diacylglycerol kinase (DGK) in the PI cycle. Thus, heavy isotope enrichment in this experiment reflects either acyl-chains acquired during de novo synthesis followed by remodeling, or recycling in the PI cycle, and the relative contribution of them might depend on the cell type. Therefore, none of the labelling strategies is perfect for discriminating metabolic pathways, and thus extreme care is required for the interpretation. For this reason, the authors are recommended to provide an illustration about which step the heavy isotopes are supposed to be incorporated, for example in Figure 1A.

We completely agree with the referee's analysis here. We have added small illustrations to Figure 2 (panels A,D,I) to make the individual labelling strategies clearer. We thank the referee for this suggestion.

In addition, a clear discrimination between de novo synthesis and PI recycling is critical for the interpretation of water labelling experiments, thus the authors should repeat the experiments in the presence of a DGK inhibitor. DGK inhibition will remove the contribution of the PI cycle in water labelling experiments, and thus the differences in acyl-chain profiles of labelled PI with or without the inhibitor will reflect what are the species that are recycled in the PI cycle. This will provide a stronger evidence that 38:4 species are more efficiently recycled than other species (if it is indeed the case).

We thank the referee for making this excellent suggestion. We now report the extended use of DGK-inhibitors (their effects were only previously reported in

stimulated HEK293 cells; Fig EV6B) on basal PA and PI synthesis in water-labelling experiments (new Figs 3A and C); this new data clearly supports a critical role for DGKs in the C38:4-selective, PI-recycling pathway.

3. The term "specific activity" is used throughout the article, but not clearly defined (only in Figure 2 legend) and its significance is not clear. This term is in general used for radioisotopes, thus it would be beneficial for readers to mention in the main text how authors define specific activity and how this can be interpreted. According to what we read, "specific activity" seems to be the level of an isotope-labelled species normalized with the sum of this species and the corresponding non-labelled species, but what could this reflect? The authors consider it to illustrate how fast a species will turnover, which indeed could be true.
The referee makes some good points here and we have replaced the term ‘specific activity’ with ‘fractional enrichment’, to make this term clearer (P5 and replaced in all Figures). We have also added further explanation for how we present our labelling studies in the Materials and Methods section (P20/21). The referee is correct, under steady-state conditions, fractional enrichment will reflect how fast the pool is turned over.
However, it could also mean that a species with low "specific activity" have additional synthetic pathways to explain its steady-state levels, and thus concluding something from specific activity sounds very challenging.
We think the referee is referring here to the possibility that there may be multiple pools of a particular species with widely different turnover rates; indeed, we acknowledge this difficulty when discussing the shape of some of the labelling curves (P6, third paragraph, last 4 lines). Nonetheless, we believe fractional enrichment can be a very useful measure that allows an estimate of pool turnover that is independent of its relative size. This allows us to conclude that the C32:0-PI pool is turned over very quickly (eg Fig 2C,F,K), despite the fact that it accumulates very little label (eg Fig 2B,E,J); which provides an important argument for the initial synthesis and subsequent remodelling of this species. An appreciation of fractional enrichment is also important when trying to understand precursor-product relationships eg the fraction of the PA pool labelled will determine the incorporation of label into PI. Further, the rate of fractional enrichment in the labelling of PI itself is a robust measure of the synthesis of new PI-species compared to their initial proportions (because total PI levels are effectively constant under both basal and stimulated conditions (eg Fig 7, relabelled panel F). Finally, the use of fractional enrichment also allows us to compare more acyl chain species than those for which we have cross-calibrations for detection efficiency (eg Fig EV2) and also to make more robust comparisons between individual cell clones that may differ in their relative pool sizes and degrees of stimulation.
Then, "relative specific activity" found in Figures 7H and 7J has even more calculations behind, and its interpretation is even more complicated, although no detailed explanations are made.
Comparing the water-labelling of PI/PA on ATP-stimulation of independently-derived clones of WT and MCF7 cells is complicated by small differences in pool sizes and sensitivity to stimulation, as well as the normal variation between experiments. Comparing the fractional enrichment of C38:4-PI/PA versus C34:1-PI/PA allows the merging of data from multiple clones and experiments and helps visualise that C38:4-PI synthesis is not as favoured over other species in CDS2-KO clones as it is in WT clones (this would be difficult to discern from the type of data presentation in

Fig 7, newly labelled panels G and H). We have added a further note of explanation in the legend to Fig 7.
The experiments of Figure 7 are very important to interpret the enzymes implicated in the efficient recycling of PI 38:4, thus a better explanation and interpretation is required.
We have now included new DG data (new Fig 7C) and substantially rewritten the relevant text (P11, P15) to make our interpretation of this collection of evidence pointing to acyl chain specificity at the DGK and CDS steps much clearer.
I would thus suggest avoiding discussing too much from these specific activity values, unless the authors can demonstrate the clear benefit of analyzing these parameters.
We hope we have now convinced the referee of the benefit of using fractional enrichment data.
4. One very difficult aspect of this study is that the acyl-chains of recycled PI are necessarily affected by the pre-existing pool of PI. Indeed, the authors convincingly demonstrate that PLC-derived DAG is efficiently converted back into precursors for PI synthesis (Figures 6J and K and EV6B), thus an enrichment of 38:4 species during recycling would not necessarily demonstrate acyl-chain specific recycling, but rather could be a consequence of 38:4 enrichment in the pre-existing PI pool, especially in HEK293 and BMDMs. Therefore, 38:4 being rich in labelling experiments analyzing PI recycling is not a demonstration of the specificity of this pathway, in the case of HEK293 and BMDMs. For this reason, the authors are completely right to mention that the diversity of PI in MCF7 offers an opportunity to study the selectivity for recycling (page 10). Indeed, the authors take advantage of this diversity in Figure 7, in which they demonstrate that PI recycling upon ATP stimulation can enrich 38:4 species despite the relative paucity of PI 38:4. It will be critical to investigate whether the PI cycle enriches PI 38:4 also in unstimulated conditions, for which water labelling experiments in MCF7 with a better discrimination of de novo synthesis and PI cycle (see above comments) could give answers.
We agree with much of the referee’s analysis here, but would argue that it’s difficult to make a clear distinction between ‘unstimulated’ and stimulated (ie ATP) conditions, because the unstimulated cells were grown in serum ie are we really comparing low levels of chronic stimulation to high levels of acute stimulation and are the same underlying processes are at play (a comment has been added to this effect, P14)? Some evidence that a C38:4-selective recycling pathway is operating [at a modest level] in basal MCF7 cells is the comparison between water-labelling (Fig 5E) versus glucose labelling (Fig 5A) of PI species, but whether this is the same as the C38:4-selective pathway operating on ATP stimulation is something we are unable to answer at present.

Minor concerns
5. The acyl-chains of PI species that increase upon lipin inhibition are coming from the increased PA pool, thus would be strongly affected by lipin substrate preference. It is therefore difficult to conclude whether they would be identical to PI species synthesized de novo when lipin is not inhibited.
In principle, this is a fair point, but lipin-inhibition caused very similar, parallel effects on all the early glucose-labelled species and also their unlabelled counterparts, suggesting lipins act on all the main PA species made de novo.

6. In Figure 6, the authors use a phospholipase D inhibitor to investigate the contribution of PLC indirectly. A more direct demonstration of the roles of PLC would be possible if using PLC inhibitors. Is there a reason why this was not done?
We did indeed use a PLC inhibitor (U73122) to confirm the central role of this enzyme in the production of PA species, this data was presented in Fig EV6B (we now draw attention to this in the text, P9).

7. As mentioned by the authors, culture conditions strongly affect polyunsaturated fatty acid levels, thus have a strong impact on PI 38:4. It is therefore very important to explain how the cells were prepared (how many days of culture before labelling experiments, cell densities) for the experiments. Otherwise, it is difficult to know whether the differences between MCF7 cells and the others are due to cell type differences or due to different culture conditions.
These are very good points. First, we have now added more cell culture details into the methods. Second, while we have routinely cultured the cell lines in their preferred medium (for long term stability), we have also cultured different cells for short periods of time under identical conditions and we still see similar, qualitative differences in their C38:4-PI enrichment, indicating a genetic basis for this diversity. We now include data from a panel of breast epithelial cell lines grown under the same conditions in Fig EV1E. For the referee's interest, we also include below some further data for HEK293 and MCF7 cells grown under different, but parallel conditions (Figure 2).

Figure for referees not shown

8. Figure 1A, what is released during phospholipase reactions is not acyl-CoA, but a free fatty acid.
Thank you for noticing this mistake, this has now been corrected.

9. Mass spectrometry is key for the study, thus it is surprising to see that almost no explanations are made about the methods. What are the mobile phases used, which type of mass spectrometer is used, and whether targeted or untargeted approaches are used, are all missing.
We apologise for this omission and thank the referee for pointing this out. We now include further details of HPLC and mass spectrometer parameters in Appendix

Tables S1, S2 and S3.

10. In Figure EV3, a reduction in CDS1 mRNA does not demonstrate that it is knocked out. The authors should demonstrate that genome DNA has indeed been fully mutated (for example by TIDE or next generation sequencing).
We apologise for omitting some of this data, we did indeed use TIDE and next generation sequencing to confirm mutations and this new information is presented in Appendix Fig S4B-E

11. The calculations made by the authors "We estimate that the maximum contribution that the pathway reported by..." at the end of the second part is important for the conclusions of the paper, but there is no explanation about how this was calculated.
We now include more details of these calculations in the text (P7).

12. PI 38:4 is clearly important in vivo, considering the strong phenotypes of Mboat7 knockout mice and the genetic diseases caused by MBOAT7 deficiency. This should be at least mentioned in the text.
The problem with reading too much into these genetic perturbations is that they cause additional effects beyond C38:4-enrichment eg altered levels of total PI, TG etc Nonetheless, we have now referenced these studies (P15).

Suggestions
13. A more detailed understanding about enzymes implicated in the processes of remodeling and PI cycle would drastically improve the manuscript. The authors use knockout cells of CDS1, CDS2, or MBOAT7 only occasionally.
The use of the term ‘occasionally’ here is harsh. We used CRISPR-directed approaches to knock-out CDS1 or CDS2 in HEK293, MCF7 and MCF10a cells and present data from multiple labelling studies on multiple clones. We also used multiple labelling strategies on WT and LPIAT1-KO BMDMs derived from genetically-modified mice.
More genetic approaches would be very interesting, especially if done in the cell line. For example, labelling experiments in MBOAT7 knockout HEK293 cells would strongly complement the study. Otherwise, DGK epsilon is also a very important enzyme in the 38:4-selective recycling process, thus its knockout would be also very interesting. It is indeed very surprising to see so little discussion about this enzyme in this manuscript.
We present new data from knocking-down the expression of six different isoforms of DGK in HEK293 cells (new Fig 3B,D). Based on the established selectivity of DGK epsilon in vitro we also expected to see a more profound effect of knocking down this specific isoform on C38:4-PI enrichment (which we now note in the text, P15, first paragraph), however, this result is in agreement with a recent study indicating overexpression of this isoform had minimal effects on the C38:4-complement of DG, PA or PI in HEK cells (new reference, Ware et al).

1st Revision - Editorial Decision 5th May 2022

Dear Dr Hawkins,

We have now received re-review reports from the two referees to whom I sent the manuscript back for a second time. You have addressed the concerns of both satisfactorily (see below; referee 3 has spotted a small issue with figure 1).

However, before I can finally accept the manuscript for publication there here are a few small editorial changes which you will need to make. These are listed in the "Data edited MS", which can be found among the manuscript files.

Would you please add a "Data Availability" section.
Regarding figure legends, please re-organise with panels listed in alphabetic order.

Our figure check has found an inconsistency between the use of error bars to indicate standard error and a sample size of 2 in Fig 2; Fig 3B and D; Fig 4A-F; Fig 5; Fig 6J; Fig 7A, E, K, L; Fig EV1E; Fig EV2; EV5A-D, I-K. Please reconcile this discrepancy.

Please reduce the number of keywords to 5.

We updated our journal's competing interests policy in January 2022 and request that authors consider not only actual and perceived competing interests but also other personal, professional, and institutional associations related to the work. Please review the policy https://www.embopress.org/competing-interests and update your competing interest statement if necessary. Also, please rename the 'Conflict of Interests' section 'Disclosure statement and competing interests'.

CRediT has replaced the traditional author contributions section because it offers a systematic machine readable author contributions format that allows for more effective research assessment. You are encouraged to use the free text boxes beneath each contributing author's name to add specific details on the author's contribution. More information is available in our guide to authors.

Please consider adding an ORCID ID: for Dr Stephens

Please include all figures (also EV figures) as individual files.

Please add callouts to figures 2d 5C, 5F, 5H in the text.

Please add legends to the Expanded View figures, refering to them as EV1, EV2 etc.

Please remove DOI links from the reference list.

Please add a table of contents to appendix file 1.

We encourage the publication of source data, particularly for electrophoretic gels and blots, with the aim of making primary data more accessible and transparent to the reader. It would be great if you could provide me with a PDF file per figure that contains the original, uncropped and unprocessed scans of all or key gels used in the figures. The PDF files should be labeled with the appropriate figure/panel number, and should have molecular weight markers; further annotation could be useful but is not essential. The PDF files will be published online with the article as supplementary "Source Data" files. Source Data can also include Excel tables to accompany your graphs. We anticipate that their inclusion will make your work more discoverable and useable to scientists in the future.

On publication, we include a synopsis of the paper (see http://emboj.embopress.org/). Please provide me with a general summary statement and 3-5 bullet points that capture the key findings of the paper.

We also need a summary figure for the synopsis. The size should be 550 pixels wide by [200-400] pixels high. You can also use something from the figures if that is easier.

Best wishes,

William

William Teale
Editor | EMBO Journal

Referee #2:

This is a careful revision of a ms that was even in its first version quite compeling and novel. Through additional experiments, the authors convincingly show the contribution of both DGK and CDS2 in acyl chain selection in the PI recycling pathway. Furthermore, the authors have made a lot of effort at clarifying the rationale of their assays by textual and figure changes. I recommend publication

Referee #3:

The authors have answered all points adequately, and the conclusions of the manuscript have been reinforced with the additional experiments. The difference between pan-DGK inhibition and individual DGK knockdown is interesting. It shows that DGK has critical roles in the 38:4-specific recycling, while this might not be simply the property of a single enzyme (in contrast to what has been suggested in the literature for DGKE). I strongly recommend the publication of the manuscript.

Just one very minor point: In Figure 1D, the product of G3P acyltransferase is 2-LPA (the lyso is at the sn-2 position of the glycerol backbone).

The authors have made all requested editorial changes.

Accepted **12th May 2022**

Dear Dr. Hawkins,

I am pleased to inform you that your manuscript has been accepted for publication in the EMBO Journal.

Congratulations on a very impressive piece of work!

Please note that it is EMBO Journal policy for the transcript of the editorial process (containing referee reports and your response letter) to be published as an online supplement to each paper. If you do NOT want this, you will need to inform the Editorial Office via email immediately. More information is available here: https://www.embopress.org/page/journal/14602075/authorguide#transparentprocess

Your manuscript will be processed for publication in the journal by EMBO Press. Manuscripts in the PDF and electronic editions of The EMBO Journal will be copy edited, and you will be provided with page proofs prior to publication. Please note that supplementary information is not included in the proofs.

I note that a callout to figure 2d is still missing. Please consider adding this at the proof stage.

Please note that you will be contacted by Wiley Author Services to complete licensing and payment information. The required 'Page Charges Authorization Form' is available here: https://www.embopress.org/pb-assets/embo-site/tej_apc.pdf

Should you be planning a Press Release on your article, please get in contact with embojournal@wiley.com as early as possible, in order to coordinate publication and release dates.

If you have any questions, please do not hesitate to call or email the Editorial Office. Thank you for your contribution to The EMBO Journal.

Yours sincerely,

William

William Teale, PhD
Editor
The EMBO Journal
w.teale@embojournal.org

EMBO Press Author Checklist

Corresponding Author Name: Phillip Hawkins	
Journal Submitted to: EMBO J	
Manuscript Number: EMBOJ-2021-110038	

USEFUL LINKS FOR COMPLETING THIS FORM
The EMBO Journal - Author Guidelines
EMBO Reports - Author Guidelines
Molecular Systems Biology - Author Guidelines
EMBO Molecular Medicine - Author Guidelines

Reporting Checklist for Life Science Articles (updated January 2022)
This checklist is adapted from Materials Design Analysis Reporting (MDAR) Checklist for Authors. MDAR establishes a minimum set of requirements in transparent reporting in the life sciences (see Statement of Task: 10.31222/osf.io/9sm4x). Please follow the journal's guidelines in preparing your manuscript.
Please note that a copy of this checklist will be published alongside your article.

Abridged guidelines for figures
1. Data
The data shown in figures should satisfy the following conditions:

- the data were obtained and processed according to the field's best practice and are presented to reflect the results of the experiments in an accurate and unbiased manner.
- ideally, figure panels should include only measurements that are directly comparable to each other and obtained with the same assay.
- plots include clearly labeled error bars for independent experiments and sample sizes. Unless justified, error bars should not be shown for technical replicates.
- if n<5, the individual data points from each experiment should be plotted. Any statistical test employed should be justified.
- Source Data should be included to report the data underlying figures according to the guidelines set out in the authorship guidelines on Data Presentation.

2. Captions
Each figure caption should contain the following information, for each panel where they are relevant:

- a specification of the experimental system investigated (eg cell line, species name).
- the assay(s) and method(s) used to carry out the reported observations and measurements.
- an explicit mention of the biological and chemical entity(ies) that are being measured.
- an explicit mention of the biological and chemical entity(ies) that are altered/varied/perturbed in a controlled manner.
- the exact sample size (n) for each experimental group/condition, given as a number, not a range;
- a description of the sample collection allowing the reader to understand whether the samples represent technical or biological replicates (including how many animals, litters, cultures, etc.).
- a statement of how many times the experiment shown was independently replicated in the laboratory.
- definitions of statistical methods and measures:
  - common tests, such as t-test (please specify whether paired vs. unpaired), simple χ2 tests, Wilcoxon and Mann-Whitney tests, can be unambiguously identified by name only, but more complex techniques should be described in the methods section;
  - are tests one-sided or two-sided?
  - are there adjustments for multiple comparisons?
  - exact statistical test results, e.g., P values = x but not P values < x;
  - definition of 'center values' as median or average;
  - definition of error bars as s.d. or s.e.m.

Please complete ALL of the questions below.
Select "Not Applicable" only when the requested information is not relevant for your study.

Materials

Newly Created Materials	Information included in the manuscript?	In which section is the information available? (Reagents and Tools Table, Materials and Methods, Figures, Data Availability Section)
New materials and reagents need to be available; do any restrictions apply?	Yes	Materials and Methods and Appendix

Antibodies	Information included in the manuscript?	In which section is the information available? (Reagents and Tools Table, Materials and Methods, Figures, Data Availability Section)
For **antibodies** provide the following information:<br>- Commercial antibodies: RRID (if possible) or supplier name, catalogue number and or/clone number<br>- Non-commercial: RRID or citation	Yes	Materials and Methods

DNA and RNA sequences	Information included in the manuscript?	In which section is the information available? (Reagents and Tools Table, Materials and Methods, Figures, Data Availability Section)
**Short novel DNA or RNA including primers, probes**: provide the sequences.	Yes	Materials and Methods, Expanded View Figures and Appendix

Cell materials	Information included in the manuscript?	In which section is the information available? (Reagents and Tools Table, Materials and Methods, Figures, Data Availability Section)
**Cell lines:** Provide species information, strain. Provide accession number in repository **OR** supplier name, catalog number, clone number, and/**OR** RRID.	Yes	Materials and Methods
**Primary cultures:** Provide species, strain, sex of origin, genetic modification status.	Yes	Materials and Methods
Report if the cell lines were recently **authenticated** (e.g., by STR profiling) and tested for mycoplasma contamination.	Not Applicable	

Experimental animals	Information included in the manuscript?	In which section is the information available? (Reagents and Tools Table, Materials and Methods, Figures, Data Availability Section)
**Laboratory animals or Model organisms:** Provide species, strain, sex, age, genetic modification status. Provide accession number in repository **OR** supplier name, catalog number, clone number, **OR** RRID.	Not Applicable	
**Animal observed in or captured from the field:** Provide species, sex, and age where possible.	Not Applicable	
Please detail **housing and husbandry conditions**.	Not Applicable	

Plants and microbes	Information included in the manuscript?	In which section is the information available? (Reagents and Tools Table, Materials and Methods, Figures, Data Availability Section)
**Plants:** provide species and strain, ecotype and cultivar where relevant, unique accession number if available, and source (including location for collected wild specimens).	Not Applicable	
**Microbes:** provide species and strain, unique accession number if available, and source.	Not Applicable	

Human research participants	Information included in the manuscript?	In which section is the information available? (Reagents and Tools Table, Materials and Methods, Figures, Data Availability Section)
If collected and within the bounds of privacy constraints report on age, sex and gender or ethnicity for all study participants.	Not Applicable	

Core facilities	Information included in the manuscript?	In which section is the information available? (Reagents and Tools Table, Materials and Methods, Figures, Data Availability Section)
If your work benefited from core facilities, was their service mentioned in the acknowledgments section?	Yes	Acknowledgements

Design

Study protocol	Information included in the manuscript?	In which section is the information available? (Reagents and Tools Table, Materials and Methods, Figures, Data Availability Section)
If study protocol has been **pre-registered, provide DOI in the manuscript**. For clinical trials, provide the trial registration number **OR** cite DOI.	Not Applicable	
Report the **clinical trial registration number** (at ClinicalTrials.gov or equivalent), where applicable.	Not Applicable	

Laboratory protocol	Information included in the manuscript?	In which section is the information available? (Reagents and Tools Table, Materials and Methods, Figures, Data Availability Section)
Provide DOI OR other citation details if **external detailed step-by-step protocols** are available.	Not Applicable	

Experimental study design and statistics	Information included in the manuscript?	In which section is the information available? (Reagents and Tools Table, Materials and Methods, Figures, Data Availability Section)

Include a statement about **sample size** estimate even if no statistical methods were used.	Not Applicable	
Were any steps taken to minimize the effects of subjective bias when allocating animals/samples to treatment (e.g. **randomization procedure**)? If yes, have they been described?	Not Applicable	
Include a statement about **blinding** even if no blinding was done.	Not Applicable	
Describe **inclusion/exclusion criteria** if samples or animals were excluded from the analysis. Were the criteria pre-established?<br><br>If sample or data points were omitted from analysis, report if this was due to attrition or intentional exclusion and provide justification.	Not Applicable	
For every figure, are **statistical tests** justified as appropriate? Do the data meet the assumptions of the tests (e.g., normal distribution)? Describe any methods used to assess it. Is there an estimate of variation within each group of data? Is the variance similar between the groups that are being statistically compared?	Yes	Methods and Figure Legends

Sample definition and in-laboratory replication	Information included in the manuscript?	In which section is the information available?<br>(Reagents and Tools Table, Materials and Methods, Figures, Data Availability Section)
In the figure legends: state number of times the experiment was **replicated** in laboratory.	Yes	Figure Legends
In the figure legends: define whether data describe **technical or biological replicates**.	Yes	Figure Legends

Ethics

Ethics	Information included in the manuscript?	In which section is the information available?<br>(Reagents and Tools Table, Materials and Methods, Figures, Data Availability Section)
Studies involving **human participants**: State details of **authority granting ethics approval** (IRB or equivalent committee(s), provide reference number for approval.	Not Applicable	
Studies involving **human participants**: Include a statement confirming that **informed consent** was obtained from all subjects and that the experiments conformed to the principles set out in the WMA Declaration of Helsinki and the Department of Health and Human Services Belmont Report.	Not Applicable	
Studies involving **human participants:** For publication of **patient photos**, include a statement confirming that consent to publish was obtained.	Not Applicable	
Studies involving experimental **animals**: State details of **authority granting ethics approval** (IRB or equivalent committee(s), provide reference number for approval. Include a statement of compliance with ethical regulations.	Not Applicable	
Studies involving **specimen and field samples:** State if relevant **permits** obtained, provide details of authority approving study; if none were required, explain why.	Not Applicable	

Dual Use Research of Concern (DURC)	Information included in the manuscript?	In which section is the information available?<br>(Reagents and Tools Table, Materials and Methods, Figures, Data Availability Section)
Could your study fall under dual use research restrictions? Please check biosecurity documents and list of **select agents and toxins** (CDC): https://www.selectagents.gov/sat/list.htm	Not Applicable	
If you used a select agent, is the security level of the lab appropriate and reported in the manuscript?	Not Applicable	
If a study is subject to dual use research of concern regulations, is the name of the **authority granting approval and reference number** for the regulatory approval provided in the manuscript?	Not Applicable	

Reporting

The MDAR framework recommends adoption of discipline-specific guidelines, established and endorsed through community initiatives. Journals have their own policy about requiring specific guidelines and recommendations to complement MDAR.

Adherence to community standards	Information included in the manuscript?	In which section is the information available?<br>(Reagents and Tools Table, Materials and Methods, Figures, Data Availability Section)
State if relevant guidelines or checklists (e.g., **ICMJE, MIBBI, ARRIVE, PRISMA**) have been followed or provided.	Not Applicable	
For **tumor marker prognostic studies,** we recommend that you follow the **REMARK** reporting guidelines (see link list at top right). See author guidelines, under 'Reporting Guidelines'. Please confirm you have followed these guidelines.	Not Applicable	
For **phase II and III randomized controlled trials**, please refer to the **CONSORT** flow diagram (see link list at top right) and submit the CONSORT checklist (see link list at top right) with your submission. See author guidelines, under 'Reporting Guidelines'. Please confirm you have submitted this list.	Not Applicable	

Data Availability

Data availability	Information included in the manuscript?	In which section is the information available?<br>(Reagents and Tools Table, Materials and Methods, Figures, Data Availability Section)
Have **primary datasets** been deposited according to the journal's guidelines (see 'Data Deposition' section) and the respective accession numbers provided in the Data Availability Section?	Not Applicable	
Were **human clinical and genomic datasets** deposited in a public access-controlled repository in accordance to ethical obligations to the patients and to the applicable consent agreement?	Not Applicable	
Are **computational models** that are central and integral to a study available without restrictions in a machine-readable form? Were the relevant accession numbers or links provided?	Not Applicable	
If publicly available data were reused, provide the respective **data citations in the reference list**.	Not Applicable	
